# RNA G-Quadruplex Structures Mediate Gene Regulation in Bacteria

**DOI:** 10.1128/mBio.02926-19

**Published:** 2020-01-21

**Authors:** Xiaolong Shao, Weitong Zhang, Mubarak Ishaq Umar, Hei Yuen Wong, Zijing Seng, Yingpeng Xie, Yingchao Zhang, Liang Yang, Chun Kit Kwok, Xin Deng

**Affiliations:** a Department of Biomedical Sciences, City University of Hong Kong, Kowloon Tong, Hong Kong SAR, China; b Department of Chemistry, City University of Hong Kong, Kowloon Tong, Hong Kong SAR, China; c Singapore Centre for Environmental Life Sciences Engineering (SCELSE), Nanyang Technological University, Singapore; d School of Medicine, Southern University of Science and Technology (SUSTech), Shenzhen, Guangdong, China; e Shenzhen Research Institute of City University of Hong Kong, Shenzhen, People’s Republic of China; College of Veterinary Medicine, Cornell University

**Keywords:** RNA G-quadruplexes (rG4), bacteria, gene regulation, nucleic acid structures, prokaryotes, transcriptome-wide

## Abstract

Guanine (G)-rich sequences in RNA can fold into diverse RNA G-quadruplex (rG4) structures to mediate various biological functions and cellular processes in eukaryotic organisms. However, the presence, locations, and functions of rG4s in prokaryotes are still elusive. We used QUMA-1, an rG4-specific fluorescent probe, to detect rG4 structures in a wide range of bacterial species both *in vitro* and in live cells and found rG4 to be an abundant RNA secondary structure across those species. Subsequently, to identify bacterial rG4 sites in the transcriptome, the model Escherichia coli strain and a major human pathogen, Pseudomonas aeruginosa, were subjected to recently developed high-throughput rG4 structure sequencing (rG4-seq). In total, 168 and 161 *in vitro* rG4 sites were found in E. coli and P. aeruginosa, respectively. Genes carrying these rG4 sites were found to be involved in virulence, gene regulation, cell envelope synthesis, and metabolism. More importantly, biophysical assays revealed the formation of a group of rG4 sites in mRNAs (such as *hemL* and *bswR*), and they were functionally validated in cells by genetic (point mutation and *lux* reporter assays) and phenotypic experiments, providing substantial evidence for the formation and function of rG4s in bacteria. Overall, our study uncovers important regulatory functions of rG4s in bacterial pathogenicity and metabolic pathways and strongly suggests that rG4s exist and can be detected in a wide range of bacterial species.

## Abstract (precis)

G-quadruplex in RNA (rG4) mediates various biological functions and cellular processes in eukaryotic organisms. However, the presence, locations, and functions of rG4 are still elusive in prokaryotes. Here, we found that rG4 is an abundant RNA secondary structure across a wide range of bacterial species. Subsequently, the transcriptome-wide rG4 structure sequencing (rG4-seq) revealed that the model E. coli strain and a major human pathogen, P. aeruginosa, have 168 and 161 *in vitro* rG4 sites, respectively, involved in virulence, gene regulation, cell envelope, and metabolism. We further verified the regulatory functions of two rG4 sites in bacteria (*hemL* and *bswR*). Overall, this finding strongly suggests that rG4s play key regulatory roles in a wide range of bacterial species.

## INTRODUCTION

G-quadruplexes (G4s), secondary structures with stacked G-quartets containing four guanines (G’s) connected by H-bonding, are widely found in both eukaryotic DNA and mRNA (1–6). Compared with G4s in DNA, those in RNA, called rG4s, exhibit greater stability (7, 8). G-rich RNA sequences can fold into G-quartets, which are further stabilized by the presence of potassium ions (K^+^), but not lithium ions (Li^+^) (9), in the center. G-quartets stacked together with connecting nucleic acid loops form the rG4 structure. Previous studies have shown that rG4s play important roles in transcription and translation processes (10, 11) and are associated with many human diseases (12–14). When present in coding sequence (CDS), rG4s can impede translation (15), facilitate ribosomal frameshift (16, 17), and stimulate cotranscription (18, 19). rG4s present in the 5′ untranslated region (UTR) and 3′ UTR of mRNA also play significant roles in suppressing translation (20–22). In particular, rG4s in the 3′ UTR influence microRNA targeting (23, 24), RNA localization (25), and alternative polyadenylation (26). These findings indicate that rG4s perform crucial regulatory functions during posttranscriptional events and RNA metabolism in eukaryotes.

In one study, transcriptome-wide profiling of rG4 distribution in humans revealed that the density of rG4s in the UTR is 4- to 5-fold higher than that in CDS (27), but the densities are not significantly different between the 5′ UTR and 3′ UTR (27). Although sequence data analysis revealed an rG4 motif of G_3+_N_1–7_G_3+_N_1–7_G_3+_N_1–7_ (9), other motifs such as two G-quartets and G-rich sequences (G% > 40%), bulges, and longer loops are also reported (4, 28, 29).

In bacteria, G4s of DNA are widely distributed, conserved, and enriched in regulatory regions that perform critical functions in replication (30), transcription (31–34), and translation (32). A DNA-RNA hybrid G-quadruplex formed in bacterial cells mediates transcription termination (35). The stabilization of G-quadruplex in the gene promoter region affects gene transcription (36). In particular, G4s regulate bacterial virulence and antigenic variation (37). Recently, G4 sequencing (G4-seq) revealed the prevalence and enrichment of putative G4 sequences (PQS) in Escherichia coli genomic DNA (6).

A recent study showed that few rG4s are present in bacteria; in particular, only one rG4 site was found in Pseudomonas putida (38). In the present study, cell imaging, rG4 structure sequencing (rG4-seq), and subsequent biophysical, functional, and phenotypic validations revealed the formation and regulatory role of rG4 in bacterial mRNA. Notably, the analyses of several biologically important bacterial species demonstrated that rG4s perform important regulatory functions in bacterial pathogenicity and metabolic pathways, which strongly suggests that rG4s play key regulatory roles in a wide range of bacterial species.

## RESULTS

### rG4s are ubiquitous in a wide spectrum of bacterial species.

Although eukaryotic rG4s have been extensively studied in recent years (4, 11), their presence in bacteria has remained poorly understood. To investigate this issue, 10 diverse model bacterial species including seven Gram-negative strains (Pseudomonas aeruginosa, Pseudomonas syringae, E. coli, Klebsiella pneumoniae ATCC 13883, Vibrio parahaemolyticus vp001, Acinetobacter strain ATCC 25922, and Salmonella enterica serovar Typhimurium PY1) and three Gram-positive strains (Staphylococcus aureus, Enterococcus faecalis, and Bacillus cereus) were selected to investigate the presence of rG4 using QUMA-1, an rG4-specifc fluorescent probe that emits red fluorescence *in vitro* and in live cells (39).

**FIG 1:**
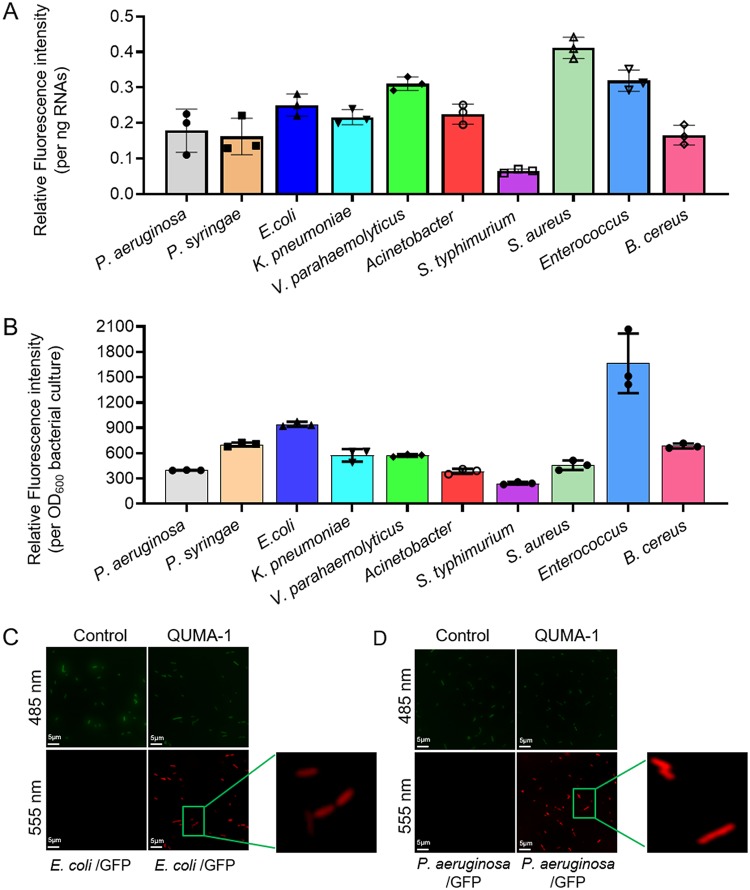
rG4 can be detected and visualized *in vitro* and in live bacterial cells. (A) rG4 was detected by the rG4-specific dye QUMA-1 in total RNAs. Tris-HCl buffer (10 mM, pH 7.4) with 100 mM KCl without QUMA-1 or with LiCl was used as the negative control. The fluorescence intensity at 670 nm was detected when the sample was excited at 555 nm in a Synergy 2 plate reader (BioTek). The relative fluorescence intensity value was normalized by using the ratio between fluorescence intensity value and the concentration of RNAs (per ng). (B) rG4 was detected by the rG4-specific dye QUMA-1 in live bacterial strains. The fluorescence intensity at 670 nm was detected when the sample was excited at 555 nm in a Synergy 2 plate reader (BioTek). The fluorescence intensity value was normalized by using the ratio between fluorescence intensity value and OD_600_ (per OD_600_). (C) rG4 structures were visualized in E. coli. (D) rG4 structures were visualized in P. aeruginosa. All the experiments were performed in at least three repetitions. Digital images were recorded by using an Eclipse Ni-E microscope (Nikon) with a 100× lens objective.

The proportion of rG4s in total RNA samples of these strains was first detected using QUMA-1 in Tris-HCl buffer (10 mM, pH 7.4) with 100 mM KCl and with 100 mM LiCl as a control. All strains were grown under common laboratory conditions for RNA extraction, and all total RNA samples showed significant fluorescence signals of rG4s (Fig. 1A). These rG4 signals were higher in K. pneumoniae and three Gram-positive strains than in other strains (Fig. 1A), indicating species-specific diversity of rG4 distribution. To validate the specificity of QUMA-1 for RNA, all total RNA samples were treated with RNase A, which led to complete loss of the fluorescent signal. The negative control (DNase I-treated RNA samples) showed no loss of signal upon DNase I treatment (see Fig. S1 in the supplemental material). Besides, both rRNAs and 23S rRNA oligonucleotides led to complete loss of the fluorescent signal induced by QUMA-1 ligand. The total RNAs showed significantly higher fluorescent signal than total rRNA (Fig. S2A to G). To further validate the presence of rG4 in live bacterial cells, the strains were cultured in nutrient-rich liquid LB medium to mid-log phase (OD_600_ = 0.6), followed by the addition of QUMA-1. Notably, all live bacterial cells emitted a strong fluorescence signal compared with the control groups (without QUMA-1 treatment), indicating the presence of rG4 in live cells (Fig. 1B). In particular, when QUMA-1-treated E. coli and P. aeruginosa carrying a green fluorescent protein (GFP)-reporter plasmid were visualized under a laser scanning confocal microscope, a strong red fluorescence signal was observed, whereas the control samples without QUMA-1 exhibited no red fluorescence signal under the same conditions (Fig. 1C and D). These results strongly suggest that rG4s are widespread across several bacterial species.

10.1128/mBio.02926-19.2 FIG S1 RNase A-treated RNA samples led to complete loss of the fluorescent signal. As a negative control, there was no loss of signal upon DNase I treatment. (A) P. aeruginosa. (B) P. syringae. (C) E. coli. (D) Acinetobacter. (E) K. pneumoniae. (F) V. parahaemolyticus. (G) *S.* Typhimurium. (H) S. aureus. (I) *Enterococcus*. (J) Bacillus cereus. All the RNA samples were divided into 6 groups (RNA + QUMA-1, RNA + RNase A + QUMA-1, RNA + DNase I + QUMA-1, RNA, Tris-KCl buffer + QUMA-1, and Tris LiCl buffer + QUMA-1), respectively. Each group had three repetitions, and results represent means ± SD. The RNA samples were first treated by RNase A or DNase I in a Tris-KCl buffer, and the control samples also had the equal volume of Tris-KCl buffer added in a total of 100 μl of reaction mixture. After the treatment, 0.5 μM (final concentration) QUMA-1 was added, mixed well, and then incubated at room temperature for 2 min. The fluorescence intensity at 670 nm was detected when the sample was excited at 555 nm in a Synergy 2 plate reader (BioTek). Download FIG S1, TIF file, 0.8 MB. Copyright © 2020 Shao et al. 2020 Shao et al. This content is distributed under the terms of the Creative Commons Attribution 4.0 International license.

10.1128/mBio.02926-19.3 FIG S2 The rRNA samples showed no fluorescent signal. (A and B) rRNA of P. aeruginosa and E. coli was detected by QUMA-1 ligand-enhanced fluorescence assay. (C) The relative fluorescence intensity (per ng) of total RNA was higher than the total rRNA in both P. aeruginosa and E. coli. (D to G) The four synthetic E. coli 23S rRNA oligonucleotides (RlmF, 5′-CCCCAAACCGACAC-3′ and 5′-AGGUGCUCAGGU-3′; RlmJ, 5′-AACUCGCUGUG-3′ and 5′-AAGAUGCAGUGUACC-3′) showed no significant difference between KCl and LiCl conditions in the QUMA-1 ligand-enhanced fluorescence assay. Each group had three repetitions. Download FIG S2, TIF file, 0.7 MB. Copyright © 2020 Shao et al. 2020 Shao et al. This content is distributed under the terms of the Creative Commons Attribution 4.0 International license.

### Transcriptome-wide rG4-seq reveals a distinct distribution of rG4s in E. coli.

**FIG 2:**
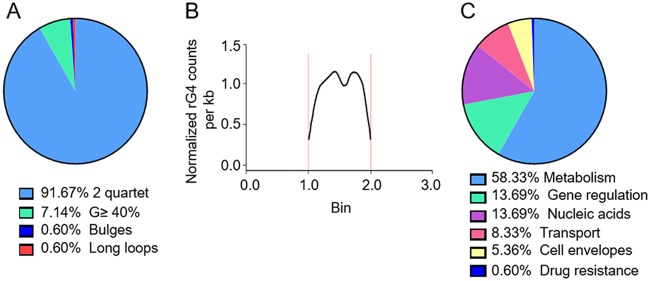
rG4-seq revealed the rG4 functions in E. coli. (A) The percentage of rG4s in different rG4 subtypes. (B) rG4 density per kilobase in different regions of mRNA. Each gene was divided into three regions, 5′ UTR, CDS, and 3′ UTR. We normalized the length of each part of each gene to the corresponding bin. The size of each bin is 1; 0 to 1 means 5′ UTR, 1 to 2 means CDS region, and 2 to 3 means 3′ UTR. The region between two vertical red lines is the CDS region. The curve indicates the density of the RTS site at each relative position. As this graph shows, the density curve has two peaks and one valley in the middle, indicating that most of the RTS sites are located on two sides of the midline of the CDS region. (C) The functional classification of rG4 sites in E. coli.

rG4-seq was performed to profile specific *in vitro* rG4 sites in the E. coli transcriptome. The mRNA samples for rG4-seq were purified by removing >90% of rRNAs, as described in Materials and Methods. The rG4-seq analysis revealed 168 rG4 sites in the E. coli transcriptome (*P* < 0.05) (Table S1A). Using a hierarchical assignment, reverse transcription (RT) stalling (RTS) sites were identified under K^+^ conditions and classified into five structural types, G_3_L_1–7_, long loops, bulges, G ≥ 40%, and 2-quartet (27). Of the identified RTS sites, 91.67% were 2-quartet type, 7.14% were G ≥ 40%, 0.6% were long loops, and 0.6% were bulges (Fig. 2A). None of the identified RTS sites corresponded to the G_3_L_1–7_ type (a canonical rG4 type in eukaryotes), indicating a unique feature of bacterial rG4s. All of the rG4 sites were located in the CDS region (Fig. 2B), with two rG4 density peaks observed near the start and end of the CDS (Fig. 2B).

10.1128/mBio.02926-19.9 TABLE S1 (A) Whole-genome location analysis of rG4 sites from rG4-seq *in* E. coli. (B) Whole-genome location analysis of rG4 sites from rG4-seq in P. aeruginosa. (C) Comparisons between this study and that by Guo and Bartel (Science 353:aaf5371, 2016, https://doi.org/10.1126/science.aaf5371). Download Table S1, PDF file, 1.7 MB. Copyright © 2020 Shao et al. 2020 Shao et al. This content is distributed under the terms of the Creative Commons Attribution 4.0 International license.

Further, the rG4-enriched mRNAs of E. coli were found to be associated with diverse biological processes, including metabolic processes (58.33%), gene regulatory processes (13.69%), nucleic acid synthesis (13.69%), transport (8.33%), cell envelope synthesis (2.36%), and drug resistance (0.6%) (Fig. 2C). Some of these mRNAs include *hemL* (encoding glutamate-1-semialdehyde aminotransferase), *bamA* (encoding the outer membrane protein assembly factor BamA, which plays a role in contact-dependent growth inhibition), *mreB* (encoding dynamic cytoskeletal protein MreB), *yqjH* (encoding a putative siderophore-interacting protein), *osmY* (encoding salt-inducible putative ABC transporter periplasmic binding protein), *uspA* (encoding a universal stress global response regulator), *fabR* (encoding transcriptional repressor of *fabA* and *fabB*), *arcB* (encoding aerobic respiration control sensor histidine protein kinase), and *acrB* (encoding multidrug efflux pump RND permease AcrB) (Table S1A). Taken together, the rG4 functional distribution pattern suggests that rG4s play important roles in these biological processes.

### rG4s in *hemL* CDS regulate *hemL* expression in E. coli.

**FIG 3:**
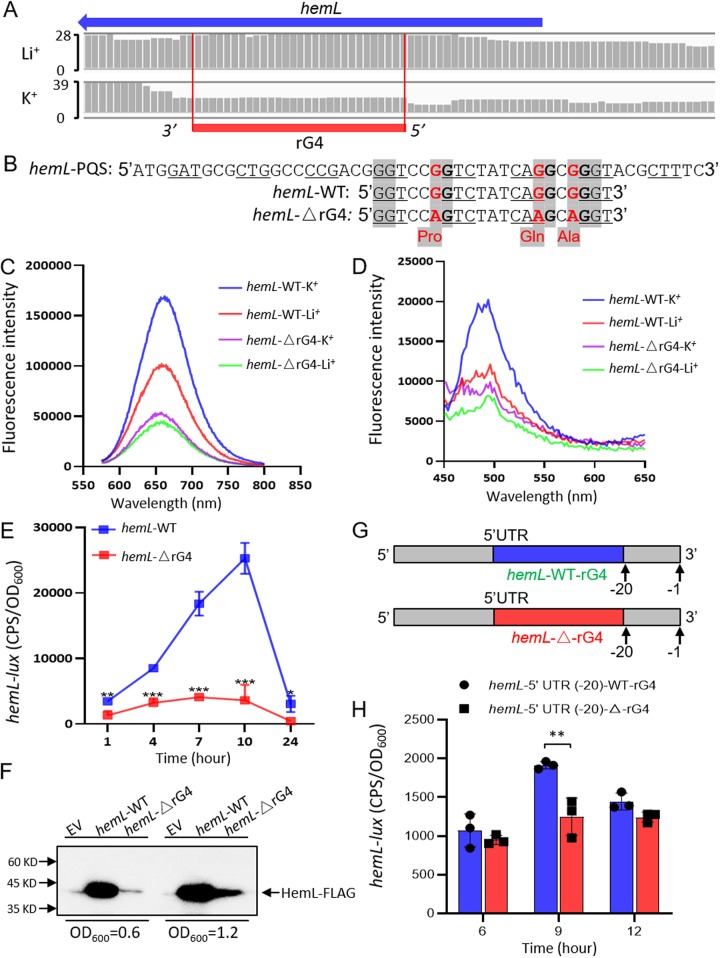
rG4 positively regulated the expression of HemL in E. coli. (A) mRNA transcripts harboring rG4 in *hemL*. (B) The mutation of rG4 in *hemL* CDS. (C) QUMA-1 specifically bound to the rG4 formed in *hemL* mRNA; the ligand-enhanced fluorescence spectra showed an enhanced fluorescence for the wild type under K^+^ compared to Li^+^ conditions. The rG4 mutant was used as a control. (D) ThT specifically bound to the rG4 in the *hemL* mRNA, producing an enhanced fluorescence signal for the wild type under the K^+^ conditions compared to the Li^+^ conditions. The rG4 mutant was used as a control. (E) The folded rG4 structure positively regulated the expression of *hemL.* All the experiments were performed in at least three repetitions. Significance is indicated as follows: *, *P* < 0.5; **, *P* < 0.01; ***, *P* < 0.001. Results represent means ± SD. (F) The folded rG4 structure positively regulated the protein expression of HemL. All the experiments were performed in at least three repetitions. EV, pAK1900 empty vector. (G) The schematic diagram of inserting *hemL*-rG4-WT into the 5′ UTR (−20 from ATG) of *hemL*-rG4-WT and *hemL*-ΔrG4-WT. (H) Immunoblot probed for the translation products of the HemL-5′ UTR (−20)-WT-rG4 and HemL-5′ UTR (−20)-Δ-rG4 constructs with rG4 inserted into the 5′ UTR of HemL. All the experiments were performed in at least three repetitions. **, *P* < 0.01. Results represent means ± SD.

Among the 168 rG4 sites located in CDS, 58.33% of all identified sites were found to be involved in metabolism. To test the effect of rG4 on posttranscriptional regulation of metabolic pathways, we first viewed the reads of the rG4 sites present in CDS. The RTS sites were defined as the positions where sequencing coverage decreased sharply. As shown in Fig. 3A, the reads of the *hemL* CDS considered to harbor rG4 sharply decreased under K^+^ conditions compared with those under Li^+^ conditions, confirming the presence of rG4 in *hemL*. Therefore, we selected a 21-bp rG4 region from *hemL* CDS (**GG**TCC**GG**TCTATCAG*GC**GGG**T, named *hemL*-WT; each three underlined bases represent a codon, and the letters in bold represent the rG4 sites that can be mutated without changing protein sequence) (Fig. 3B). The guanine (G) bases present in the third position in codons were then replaced with adenine (A) (**GG**TCCA*GTCTATCA**AG**CA*GGT), to generate *hemL*-ΔrG4 (Fig. 3B; Fig. S3A and B). The mutated codons (CCG, CAG, and GCG) did not change the protein expression level compared with the wild-type *hemL*-WT *lux* reporter, respectively (Fig. S4A to C).

10.1128/mBio.02926-19.4 FIG S3 The guanine (G) bases present in the third position in codons were replaced with adenine in the *hemL* and *bswR* rG4 region. Sequencing peaks of *hemL*-WT (A), *hemL*-ΔrG4 (B), *bswR*-WT (C), and *bswR*-ΔrG4 (D). Download FIG S3, TIF file, 0.4 MB. Copyright © 2020 Shao et al. 2020 Shao et al. This content is distributed under the terms of the Creative Commons Attribution 4.0 International license.

10.1128/mBio.02926-19.5 FIG S4 The mutated codons did not change the protein expression level compared with the wild-type *hemL*-WT and *bswR*-WT *lux* reporters. (A) Three mutations in the alanine codon (CCG) in *hemL*-WT (non-rG4 region) by replacing the G with A (A is highlighted in yellow in the table). (B) Three mutations in the glutamine codon (CAG) in *hemL*-WT (non-rG4 region) by replacing the G with A (A is highlighted in yellow in the table). (C) Three mutations in the proline codon (GCG) in *hemL*-WT (non-rG4 region) by replacing the G with A (A is highlighted in yellow in the table). (D) Three mutations in the glutamine codon (CAG) in *bswR*-WT (non-rG4 region) by replacing the last G with A (A is highlighted in yellow in the table). (E) Three mutations in the leucine codon (CTG) in *bswR*-WT (non-rG4 region) by replacing the G with A (A is highlighted in yellow in the table). All the experiments were performed in at least three repetitions, ***, *P* < 0.01. Results represent means ± SD. Download FIG S4, TIF file, 0.7 MB. Copyright © 2020 Shao et al. 2020 Shao et al. This content is distributed under the terms of the Creative Commons Attribution 4.0 International license.

The fluorescence spectroscopy revealed that the mRNA oligonucleotides of *hemL*-WT exhibited an enhanced QUMA-1 fluorescence intensity under the K^+^ conditions compared with that under Li^+^ conditions, whereas those of *hemL*-ΔrG4 showed little to no difference in the intensity between K^+^ and Li^+^ conditions (Fig. 3C). This finding was confirmed using thioflavin T (ThT), another fluorescent dye specific for G4s (40). As shown in Fig. 3D, *hemL*-WT oligonucleotides yielded a significant ThT fluorescence signal at ∼495 nm under K^+^ conditions compared with that under Li^+^ conditions. In contrast, the *hemL*-ΔrG4 oligonucleotides showed little to no difference in the ThT intensity between K^+^ and Li^+^ conditions. In addition, because rG4 topology displays a characteristic circular dichroism (CD) spectrum (41, 42), CD assays were performed using a Jasco J-1500 circular dichroism spectrophotometer to detect the presence of the rG4 structure in *hemL* CDS. The *hemL*-WT RNA sequence exhibited a negative peak at ∼240 nm and a positive peak at ∼263 nm, suggesting the presence of parallel topology (Fig. S5A), but *hemL*-ΔrG4 RNA sequence showed a low rG4 signal in both K^+^ and Li^+^, with a positive peak at 270 nm and a negative peak at 244 nm (Fig. S5B).

10.1128/mBio.02926-19.6 FIG S5 CD assay showed presence of rG4 in *hemL* and *bswR* mRNA. (A and C) The CD spectrum of the wild-type oligonucleotide (from *hemL* or *bswR*) was monovalent ion dependent and showed a negative peak at ∼240 nm and positive peak at ∼263 nm under K^+^ conditions but not under Li^+^ conditions, suggesting the formation of parallel topology. (B and D) The CD spectrum of rG4-mutant oligonucleotide (from *hemL* or *bswR*) was monovalent ion independent and showed no sign of rG4 formation under both lithium and potassium ion conditions, due to the positive peak at 270 nm and a negative peak at 244 nm. Download FIG S5, TIF file, 0.3 MB. Copyright © 2020 Shao et al. 2020 Shao et al. This content is distributed under the terms of the Creative Commons Attribution 4.0 International license.

Both *hemL*-WT and *hemL*-ΔrG4 sequences were cloned into the luciferase reporter plasmid pMS402, and their luminescence (*lux*) values were measured after transformation into the wild-type E. coli strain. The expression of *hemL*-ΔrG4 was found to be significantly lower than that of *hemL*-WT, indicating that rG4 positively regulated the *hemL* expression (Fig. 3E). The real-time quantitative PCR (RT-qPCR) result also determined that rG4 positively regulated the transcription of *hemL* (Fig. S6A). To verify the *lux* reporter assay, a Western blot analysis showed that the expression of HemL-FLAG in the E. coli/pAK1900-*hemL*-WT strain was significantly higher than that in the E. coli/pAK1900-*hemL*-ΔrG4 strain (Fig. 3F), which demonstrated that rG4 positively regulates gene regulation of HemL. Taken together, our results revealed rG4-dependent regulation of the coding regions in E. coli.

10.1128/mBio.02926-19.7 FIG S6 The folded rG4 structure affected the transcriptional level of *hemL* while it did not affect the *bswR* mRNA level. (A) The folded rG4 structure positively regulated the transcriptional level of *hemL*. All the strains were cultured at 37°C and 220 rpm overnight in 2 ml LB supplemented with 60 μg/ml carbenicillin and then inoculated into 2 ml fresh LB (1:100 dilution) until OD_600_ reached 0.6. All the experiments were performed in at least three repetitions. **, *P* < 0.01. The results represent means ± SD. (B) The folded rG4 structure did not affect the mRNA level of *bswR*. All the strains were cultured at 37°C and 220 rpm overnight in 2 ml LB supplemented with 150 μg/ml carbenicillin and inoculated into 2 ml fresh LB (1:100 dilution) until OD_600_ reached 0.6. All the experiments were performed in at least three repetitions. The results represent means ± SD. NS, no significant difference. Download FIG S6, TIF file, 0.2 MB. Copyright © 2020 Shao et al. 2020 Shao et al. This content is distributed under the terms of the Creative Commons Attribution 4.0 International license.

To test whether the rG4 formed in HemL CDS affects gene regulation at the UTR, we first inserted the wild-type HemL-rG4-WT and the mutated HemL-Δ-rG4 sequences into the 5′ UTR (−20 from ATG) of HemL (Fig. 3G). As the results, the wild-type HemL-rG4 construct showed higher activity than the mutated HemL-Δ-rG4 at 9 h (Fig. 3H), indicating the positive regulation of HemL-rG4 in the 5′ UTR. In addition, we also inserted the HemL-rG4-WT sequence into its 3′ UTR (after TAA) (Fig. S7A). The Western blotting results showed that the inserted HemL-rG4-WT at the 3′ UTR did not affect the expression of either the wild-type HemL-WT-rG4 or the mutated HemL-Δ-rG4 (Fig. S7B). In sum, our results indicated that rG4 regulates gene expression in E. coli.

10.1128/mBio.02926-19.8 FIG S7 The rG4 formed in the 3′ UTR of *hemL* and *bswR* did not affect their protein expression level. (A) The wild-type HemL-rG4 sequence was moved to the 3′ UTR of *hemL*-WT-FLAG or *hemL*-ΔrG4-FLAG. (B) Western blotting detected the protein expression of *hemL*-WT-FLAG and *hemL*-ΔrG4-FLAG containing the wild-type *hemL*-rG4. The total lysate of each strain was loaded with equal quantity. All the experiments were performed in at least three repetitions. (C) The wild-type BswR-rG4 sequence was moved to the 3′ UTR of *bswR*-WT-FLAG or *bswR*-ΔrG4-FLAG. (D) Western blotting detected the protein expression of *bswR*-WT-FLAG and *bswR*-ΔrG4-FLAG containing the wild-type *bswR*-rG4. The total lysate of each strain was loaded with an equal quantity. All the experiments were performed in at least three repetitions. Download FIG S7, TIF file, 0.3 MB. Copyright © 2020 Shao et al. 2020 Shao et al. This content is distributed under the terms of the Creative Commons Attribution 4.0 International license.

### rG4-seq reveals 161 rG4 sites in P. aeruginosa.

**FIG 4:**
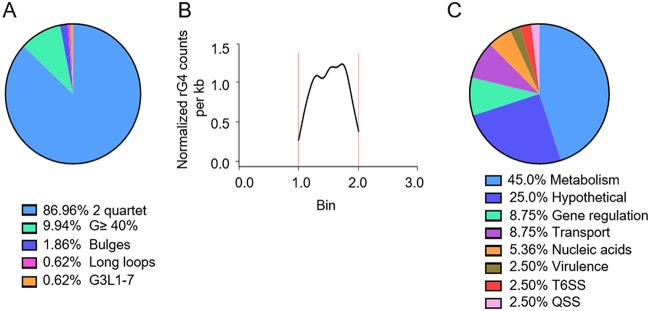
rG4-seq revealed the rG4 functions in P. aeruginosa. (A) The percentage of rG4s in different rG4 subtypes. (B) rG4 density per kilobase in different regions of mRNA. We plotted this graph by the same method described for Fig. 2B. The region between two vertical red lines is the CDS region. As this graph shown, RTS sites are mainly located in the middle part of the CDS region, especially in the position near the 3′ UTR. (C) The functional classification of rG4 sites in P. aeruginosa.

To uncover rG4 sites in P. aeruginosa transcriptome, rG4-seq was used to profile rG4 sites. As a result, rG4-seq revealed 161 *in vitro* rG4 sites in the P. aeruginosa transcriptome (*P* < 0.05) (Table S1B). All the identified RTS sites under the K^+^ conditions were classified into 5 structural types, including 86.96% as 2 quartet, 9.94% as G ≥ 40%, 1.86% as bulges, 0.62% as long loops, and 0.62% as G_3_L_1–7_ (Fig. 4A). Similarly to E. coli, all rG4 structures identified in P. aeruginosa were located at the CDS region (Table S1B), with the highest peak observed at the end of the CDS near the 3′ UTR (Fig. 4B). The rG4-associated genes in P. aeruginosa were involved in metabolic processes (45%), hypothetical protein (25%), gene regulation processes (8.75%), transport (8.75%), and nucleic acid synthesis (5.63%). Besides, rG4 sites in P. aeruginosa were also involved in virulence (2.5%), the type VI secretion system (T6SS) (2.5%), and the quorum sensing system (QSS) (1.88%) (Fig. 4C). For instance, rG4s were located in genes involved in virulence, including a regulator gene of bacterial swarming and biofilm formation (*bswR*), a type III secretion system effector gene (*exoS*), two genes associated with twitching motility (*pilU* and *pilJ*), four type VI secretion system (T4SS) genes (*clpV1*, *hisC2*, *tssA1*, and *tssG1*), three quorum sensing genes (*pqsD*, *mucP*, and *secY*), and a gene involved in multidrug efflux (*mexZ*) (Table S1B). Thus, the rG4 distribution pattern in P. aeruginosa suggests that rG4s play roles in important biological processes.

### rG4 affects swarming and biofilm formation of P. aeruginosa by enhancing *bswR* expression.

**FIG 5:**
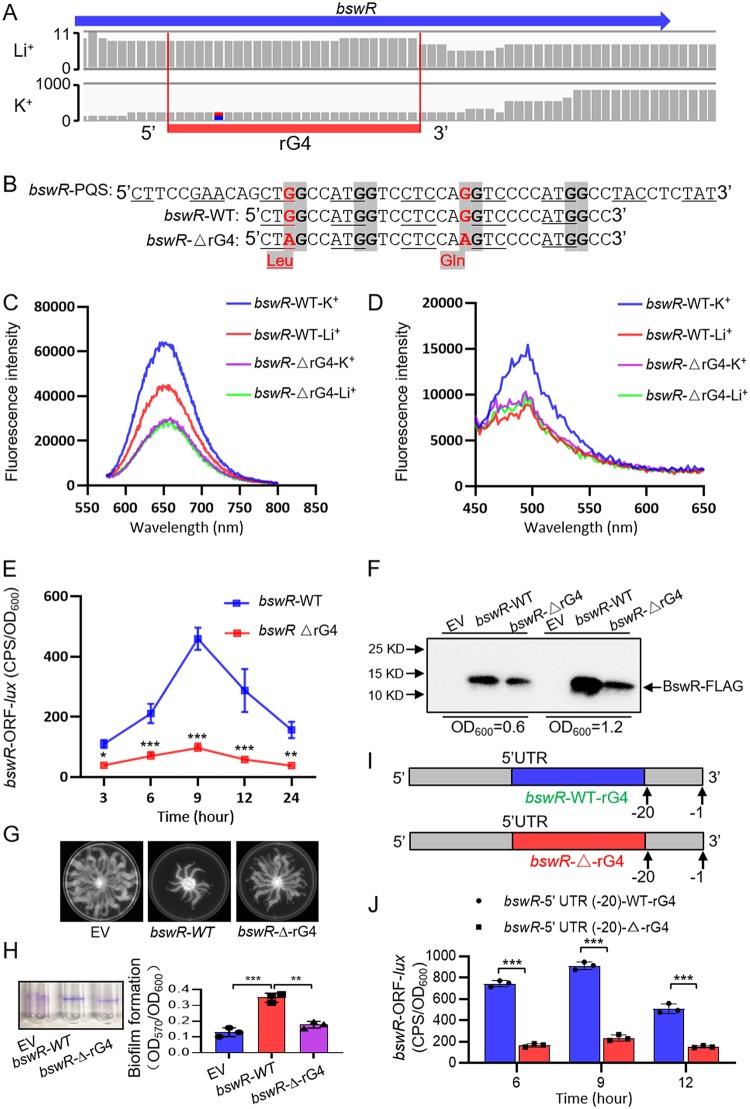
rG4 positively regulated the functions of BswR in motility and biofilm formation in P. aeruginosa. (A) mRNA transcripts harboring rG4 in the *bswR* gene. (B) The mutation of rG4 in BswR CDS. (C) QUMA-1 specifically bound to the rG4 formed in *bswR* mRNA; the ligand-enhanced fluorescence spectra showed an enhanced fluorescence for the wild type under K^+^ compared to Li^+^ conditions. The rG4 mutant was used as a control. (D) ThT specifically bound to rG4 in the *bswR* mRNA, producing an enhanced fluorescence signal for the wild type under K^+^ conditions compared to Li^+^ conditions. The rG4 mutant was used as a control. (E) The folded rG4 structure positively regulated the expression of *bswR in vivo.* (F) rG4 positively regulated the protein expression of BswR. (G) Swarming motility measurement between overexpressed *bswR*-WT and *bswR*-ΔrG4 strains. (H) Biofilm production detection between overexpressed *bswR*-WT and *bswR*-ΔrG4 strains. All the experiments were performed in at least three repetitions. *, *P* < 0.5; **, *P* < 0.01; ***, *P* < 0.001. Results represent means ± SD. (I) The schematic diagram of inserting *bswR*-rG4-WT into the 5′ UTR (−20 from ATG) of *bswR*-rG4-WT and *bswR*-ΔrG4-WT, respectively. (J) Immunoblot probed for the translation products of the *bswR*-5′-UTR (−20)-WT-rG4 and *bswR*-5′-UTR (−20)-Δ-rG4 constructs with wild-type *bswR*-rG4 inserted into the 5′ UTR of BswR. All the experiments were performed in at least three repetitions. ***, *P* < 0.01. Results represent means ± SD.

rG4-seq revealed an rG4 site in the *bswR* CDS (CT**GG**CCATG*GTCCTCCA**GG**TCCCCATG*GCC, named *bswR*-WT) (Fig. 5A and B). To verify the function of rG4 in the *bswR* CDS, the guanine (G) bases present in the third position in codons were replaced with adenine (A) to avoid amino acid mutation, resulting in CT**AG**CCATG*GTCCTCCA**AG**TCCCCATG*GCC, named *bswR*-ΔrG4 (Fig. 5B; Fig. S3A and B). The mutated codons (CAG and CTG) did not change the protein expression level (Fig. S4D and E). Notably, the QUMA-1 fluorescence intensity of the *bswR*-WT oligonucleotides was significantly higher under the K^+^ conditions than unde the Li^+^ conditions, whereas the *bswR*-ΔrG4 oligonucleotides showed no significant difference in intensity between the two conditions (Fig. 5C). The ThT assay of *bswR*-WT RNA oligonucleotides also showed a significantly enhanced fluorescence intensity under K^+^ conditions compared with that under the Li^+^ conditions (Fig. 5D). However, the *bswR*-ΔrG4 oligonucleotides showed no difference in intensity between the two conditions. Additionally, CD studies were further performed to detect the presence of rG4 structure in the *bswR* CDS region. The *bswR*-WT RNA sequence showed a negative peak at ∼240 nm and a positive peak at ∼264 nm, suggesting the presence of parallel topology (Fig. S5C), but the *bswR*-ΔrG4 RNA sequence showed no significant difference between K^+^ and Li^+^ conditions (Fig. S5D), with a positive peak at ∼270 nm.

To explore whether the presence of rG4 regulates BswR function, the native promoter of *bswR* and its CDS without the termination codon (TAA) were fused into the promoterless pMS402 plasmid carrying the luciferase gene *luxCDABE* to generate the translational *lux* reporters (*bswR*-WT and *bswR*-ΔrG4). As expected, the results showed that the activity of *bswR*-WT *lux* reporter was higher than that of *bswR*-ΔrG4 in the wild-type strain PAO1 (Fig. 5E), indicating that rG4 positively regulated BswR expression. To detect whether rG4 regulates the transcriptional level of *bswR*, RT-qPCR was performed in PAO1/pAK1900, PAO1/pAK1900-*bswR*-WT, and PAO1/pAK1900-*bswR*-ΔrG4 strains. The result showed no significant difference between the last two strains (Fig. S6B), which suggests that rG4 regulates the translation of BswR. To this end, we performed Western blotting to compare the protein expression levels of BswR-FLAG in these strains. As shown in Fig. 5F, the level of BswR-FLAG in the PAO1/pAK1900-*bswR*-ΔrG4 strain was significantly lower than that in the PAO1/pAK1900-*bswR-*WT strain, indicating the positive regulation of rG4 on the translation of BswR.

Given those findings, BswR regulates the biogenesis of bacterial flagella and biofilm formation in P. aeruginosa (43). Subsequently, the effect of overexpression of *bswR*-WT and *bswR*-ΔrG4 sequences on the swarming motility of these PAO1 strains was tested on semisolid agar plates. The overexpression of *bswR* (*bswR*-WT) repressed the swarming motility of PAO1, but that of the mutant (*bswR*-ΔrG4) showed no such inhibition (Fig. 5G). In contrast, the strain with the *bswR*-WT sequence, but not the *bswR*-ΔrG4 sequence, showed enhanced biofilm production compared with the wild-type strain PAO1 carrying an empty vector (Fig. 5H). Overall, the presence of the rG4 structure in the *bswR* RNA sequence positively regulated the functions of BswR in repressing swarming motility and enhancing biofilm formation, suggesting the important regulation of BswR-rG4 structures in P. aeruginosa virulence.

Furthermore, to explore the function of BswR-rG4 outside the CDS, we moved the BswR-rG4 to the 5′ UTR and 3′ UTR. Similarly to the construction strategies for HemL, we first inserted the wild-type BswR-WT-rG4 and the mutated BswR-Δ-rG4 sequence into the 5′ UTR (−20 from ATG) of BswR (Fig. 5I) and measured their transcriptional activities using the *lux* reporter assay. Interestingly, the resulting construct (BswR-5′ UTR-Δ-rG4) containing mutated rG4 showed lower activity than the one with the wild-type rG4 (BswR-5′ UTR-WT-rG4) (4.5-fold) (Fig. 5J). To further test the function of BswR-rG4 outside the CDS, we subsequently inserted the wild-type BswR-rG4 into the 3′ UTR after the stop codon (Fig. S7C). As result, the insertion of wild-type BswR-rG4 did not affect the translation level of either BswR-WT-rG4 or BswR-Δ-rG4 (Fig. S7D). Taken together, our results showed that rG4 structures play important roles in BswR-dependent regulation in P. aeruginosa.

## DISCUSSION

Although eukaryotic rG4s play important roles in transcription, processing, localization, and translation of mRNAs as well as noncoding RNAs (4, 11, 13), the presence and functions of rG4s in prokaryotic mRNAs are still poorly understood. Using the rG4-specific fluorescent probe QUMA-1, we demonstrated the widespread distribution of rG4s in 10 bacterial species with strong fluorescence signals in live bacterial cells. Among these strains, Gram-negative K. pneumoniae and Gram-positive S. aureus, Enterococcus faecium, and B. cereus showed higher fluorescence intensity than other tested strains, suggesting the species-specific distribution patterns of rG4s in different strains. The transcriptome-wide rG4-seq analysis previously developed by us (27) revealed 168 and 161 *in vitro* rG4 sites in E. coli and P. aeruginosa, respectively. In contrast to eukaryotic rG4s, the newly found prokaryotic rG4s were less diverse in terms of transcriptome distribution and were predominantly found in the CDS. While most newly identified and verified rG4s were located in the CDS in our work, we also showed that by relocating the rG4s to the UTRs (especially to the 5′ UTRs), they can also regulate gene expression. Although bacterial rG4s (mostly G_2_ rG4s) are less stable than rG4 with three stacks and shorter loops, we used 3 independent assays (ThT fluorescent turn-on assay, QUMA-1 fluorescent turn-on assay, and CD titration) to strongly demonstrate rG4 formation in mRNA of *hemL* and *bswR* (Fig. 3C to E and Fig. 5C to E; see also Fig. S5A to D in the supplemental material).

A previous study reports that very few rG4s are present in bacteria (38), which is less than that in our present study. This may result from the differences in experimental procedures and data analyses between two studies (Table S1C). (i) In the present study, we chose the rG4 regions that were mapped into 5 patterns (G_3_L_1–7_, long loops, bulges, G ≥ 40%, and 2-quartet). On the other hand, the previous study considers rG4 sites that stop at G and finds the rG4 similar to the canonical rG4 (G_3_L_1–7_), which was identified in only one site in P. aeruginosa in our present study. Our method may capture some rG4 sites with weak signals that were filtered out in the previous study. (ii) In data analysis, the present study cut off data by RTS scores, which were calculated by a 10-order filter indicating the drops of reads. The previous study identifies the RTS sites by using fold enrichment, which is calculated as the ratio between the number of reads of current RT stops and the average reads of all the RT stops observed. (iii) In experimental verification of rG4, the present study used multiple approaches, including thioflavin T (ThT) and QUMA-1 ligand-enhanced fluorescence assay, circular dichroism, genetic experiments (point mutation and *lux* reporter assays), and phenotypic experiments (swarming motility and biofilm formation), while the previous study used ectopic expression of G3A2 quadruplex.

Some similar and specific rG4 features were observed in P. aeruginosa and E. coli. In both strains, most of the potential rG4 RTS sites (91.07% and 86.88%, respectively) belonged to the 2-tetrad group and were widely distributed in the CDS region with high density. The biggest functional group of rG4-carrying genes was metabolic pathways (58.33% in E. coli and 45% in P. aeruginosa). We noticed that 5.36% of all rG4 sites were associated with cell envelope formation, suggesting their potential roles in cell membrane and cell wall biosynthesis in E. coli. Compared with E. coli, P. aeruginosa exhibited a higher percentage of rG4 sites involved in motility, T6SS, and QSS.

Subsequent experimental validations revealed important regulatory functions of the tested rG4 sites in *hemL* and *bswR*. All tested rG4s positively regulated the expression of rG4-carrying genes in both E. coli and P. aeruginosa (Fig. 3 and 5). Eukaryotic rG4s are involved in positive and negative regulation (22), suggesting that rG4s play crucial roles across the prokaryote and eukaryote kingdoms.

Taken together, the present study showed that rG4 structures are widespread in bacteria and perform important functions in various metabolic pathways and virulence regulation. Given their roles in virulence and pathogenesis, rG4s hold great potential to serve as therapeutic targets. The detailed regulatory mechanisms of bacterial rG4s need to be investigated in the future.

## MATERIALS AND METHODS

### Bacterial strains and mRNA purification.

The strains used in this study are listed in Table S2 in the supplemental material. Pseudomonas aeruginosa, Escherichia coli, and their derived strains were all cultured at 37°C in LB (Luria-Bertani) broth with shaking at 220 rpm or on LB solid agar plates. The concentration of antibiotics used was as follows: for Pseudomonas aeruginosa, trimethoprim at 300 μg/ml in LB and carbenicillin at 150 μg/ml in LB; for Escherichia coli, kanamycin at 50 μg/ml and ampicillin at 100 μg/ml. All experiments were performed in a biosafety level 2 (BSL-2) lab at City University of Hong Kong.

10.1128/mBio.02926-19.10 TABLE S2 Strains, plasmids, and primers used in this study. Download Table S2, PDF file, 0.7 MB. Copyright © 2020 Shao et al. 2020 Shao et al. This content is distributed under the terms of the Creative Commons Attribution 4.0 International license.

The overnight cultures of Pseudomonas aeruginosa and Escherichia coli were inoculated into 5 ml LB and grown to mid-log phase (OD_600_ of ∼0.6) at 37°C with shaking at 220 rpm. The 1-ml bacterial cultures were collected by centrifugation at 5,000 rpm for 5 min, and the pellets were stored at −80°C for total RNA extraction. The total RNAs were extracted by using the RNeasy bacterial minikit (Qiagen), and the rRNAs were further removed by using the Ribo-Zero rRNA removal kit (bacteria) (Epicentre). The total RNAs or mRNAs were quantified with a Qubit 4 fluorometer (ThermoFisher).

### QUMA-1 staining for total RNAs and live bacteria.

The methods of QUMA-1 staining for live bacteria and RNAs were performed as described in a previous study described with minor modification (39). An aliquot of total RNAs was added into Tris-HCl buffer (10 mM, pH 7.4) with 100 mM KCl containing QUMA-1 at a final concentration of 1 μM. The reaction mixture was mixed and incubated at room temperature for 5 min. The Tris-HCl buffer (10 mM, pH 7.4) with 100 mM KCl without QUMA-1 or with LiCl was used as the negative control. The fluorescence intensity at 670 nm was detected when the sample was excited at 555 nm in a Synergy 2 plate reader (BioTek). The fluorescence intensity value was normalized by using the ratio between fluorescence intensity value and the concentration of RNAs.

The overnight bacterial cells were transferred into 2 ml fresh LB and grown to mid-log phase (OD_600_ of ∼0.6) at 37°C with shaking at 220 rpm. Cells were washed two times with fresh LB and stained with 0.5 μM QUMA-1 for 3 h by directly adding QUMA-1 into the resuspended bacterial cultures in a black 96-well plate with a transparent bottom. Then, the fluorescence intensity at 670 nm was detected when the sample was excited at 555 nm, and bacterial growth was measured by OD_600_ in a Synergy 2 plate reader (BioTek) at the same time. The fluorescence intensity value was normalized by using the ratio of fluorescence intensity value to OD_600_. Digital images were recorded by using an Eclipse Ni-E microscope (Nikon) with a 100× lens objective.

### High-throughput rG4 structure sequencing and analysis.

The rG4-seq and analysis were performed as our previous study described with minor modification (27). Approximately 100 ng of bacterial mRNA was obtained after rRNA depletion. RNA fragmentation was carried out in fragmentation buffer (final concentrations: 40 mM Tris-HCl, pH 8.0, 100 mM LiCl, 30 mM MgCl_2_) at 95°C for 45 s to produce an RNA fragment size of ∼250 nucleotides (nt), followed by RNA Clean & Concentrator (Zymo Research). Next, 3′ dephosphorylation was performed by using 8 μl RNA sample, 1 μl 10× T4 polynucleotide kinase (PNK) buffer, 1 μl T4 PNK enzyme (New England Biolabs [NEB]) at 37°C for 30 min. Then, 3′ adapter ligation was conducted by adding 10 μl sample from above, 1 μl of 10 μM 3′ rApp adapter (5′-/5rApp/AGATCGGAAGAGCACACGTCTG/3SpC3/-3′), 1 μl 10× T4 RNA ligase buffer, 7 μl polyethylene glycol (PEG) 8000, and 1 μl T4 RNA ligase 2 K227Q (NEB) at 25°C for 1 h, followed by RNA Clean & Concentrator. The sample was then broken down into two parts for 150 mM Li^+^ and 150 mM K^+^ for reverse transcription (∼12 μl each), with 1 μl of 5 μM reverse primer (5′-CAGACGTGTGCTCTTCCGATCT-3′) and 6 μl of 5× reverse transcription buffer (final concentrations: 20 mM Tris, pH 7.5, 4 mM MgCl_2_, 1 mM dithiothreitol [DTT], 0.5 mM deoxynucleoside triphosphates [dNTPs], 150 mM LiCl, or 150 mM KCl). The mixture was heated at 95°C for 1.5 min and cooled at 4°C for 1.5 min, followed by 37°C for 15 min before 1 μl of Superscript III (200 U/μl) was added. The reverse transcription was carried out at 37°C for 40 min, followed by treatment with 1 μl of 2 M NaOH at 95°C for 10 min. Five microliters of 1 M Tris-HCl (pH 7.5) was added to neutralize the solution, before the sample was cleaned up by RNA Clean & Concentrator. To the purified cDNA sample (8 μl), 1 μl of 40 μM 5′ adapter (5′/5Phos/AGATCGGAAGAGCGTCGTGTAGCTCTTCCGATCTNNNNNN/3SpC3/3′) was added. The sample was heated at 95°C for 3 min and cooled to room temperature, and 10 μl of 2× Quick T4 ligase buffer and 1 μl Quick T4 DNA ligase (NEB) were added and incubated at room temperature overnight. The ligated cDNAs were purified with a precast 10% urea denaturing Tris-buffered EDTA (TBE) gel, and the 90- to 450-nt size was cut, followed by the gel extraction step using crushing and soaking methods. Next, PCR (20 μl) was performed using 95°C for 3 min, 18 cycles of each temperature step (98°C for 20 s, 65°C for 15 s, and 72°C for 40 s), 72°C for 1 min, 1 μl 10 μM forward primer (5′-AATGATACGGCGACCACCGAGATCTACACTCTTTCCCTACACGACGCTCTTCCGATCT-3′) and 1 μl 10 μM reverse primer (e.g., index 2) (5′-CAAGCAGAAGACGGCATACGAGATACATCGGTGACTGGAGTTCAGACGTGTGCTCTTCCGATCT-3′), 8 μl DNA template, and 10 μl 2× KAPA HiFi ready mix. The amplified libraries were purified with a 1.8% agarose gel for 50 min at 120 V, and the 150- to 400-bp size was sliced and extracted with the Zymoclean gel DNA recovery kit. The purified libraries underwent qPCR with the KAPA Universal Quant kit and were subjected to next-generation sequencing on a MiSeq V3 sequencer (Illumina) at the Genomics Core Facility at Nanyang Technological University, Singapore. The data analyses followed the previous pipeline (23), by replacing the genomes of E. coli K-12 strain MG1655 (ASM584v2, NC_000913.3) and *P. aeruginosa* PAO1 (ASM676v1, NC_002516.2).

### Data analysis.

First, data alignment and coverage calculation were performed. Sequencing data (150 bp/read, single-end) were trimmed by Trim Galore (http://www.bioinformatics.babraham.ac.uk/projects/trim_galore/) for removal of adapters and low-quality read sequences (parameter). The trimmed data were then aligned with the E. coli (NC_000913.3) and P. aeruginosa (NC_002516.2) reference genome by the TopHat 2 software (https://ccb.jhu.edu/software/tophat/index.shtml). The genome sequence and gene annotation files of both E. coli and P. aeruginosa for the alignment were downloaded from the NCBI database. Aligned reads with mapping quality below 30 were removed using SAMtools (http://samtools.sourceforge.net). The coverage bedGraph files for single bases were calculated using bedtools (http://bedtools.readthedocs.org/).

Then, reverse transcription (RT) stalling sites were scored. The effect of rG4 structure was characterized by the RT stalling degree measured using the RT score. The decrease of coverage, indicating stalling during the reverse transcription process, reflected an RT score close to 1. To identify the RT stalling sites, we calculated RT scores by the following analysis. The mapping results were reassembled by cufflinks (http://cole-trapnell-lab.github.io/cufflinks/), and the transcript units (TUs) were obtained. For TUs on the forward strand, the coverage signals were convolved with a 10-order filter (1 1 1 1 1 1 1 1 1 1 0 −1 −1 −1 −1 −1 −1 −1 −1 −1 −1). To normalize for the total coverage upstream of each base, the coverage signals were convolved with a 10-order filter (1 1 1 1 1 1 1 1 1 1 0 0 0 0 0 0 0 0 0 0 0), and then the ratio of the two convolved signals for each base was calculated. For TUs on the reverse strand, the coverage signals were convolved with a 10-order filter (−1 −1 −1 −1 −1 −1 −1 −1 −1 −1 0 1 1 1 1 1 1 1 1 1 1). To normalize for the total coverage upstream of each base, the coverage signals were convolved with a 10-order filter (0 0 0 0 0 0 0 0 0 0 0 1 1 1 1 1 1 1 1 1 1), and then the ratio of the two convolved signals for each base was calculated. The range of the normalized convolved signal is between −∞ and 1, where, at a given base, the ratio close to 1 represents a full drop while −∞ represents a full increase.

The local maximum of the normalized convolved signal, the RTS score, was calculated, indicating the locations of the sharp drop in coverage. For filtering some low-quality signals, for each base with local maximal signal in each replicate, the locations where the coverage of bases was below 6 and RTS scores were below 0.2 were removed.

After that, the significant RTS sites were identified. For both samples under K^+^ conditions and those under Li^+^ conditions, the calculations described above were performed. Then, the RTS scores for both K^+^ and Li^+^ conditions were used to fit a linear model (function lm in R) and estimate the *P* value of the fitting through analysis of variance (ANOVA) testing.

Finally, the sequences of the potential rG4 sites were mapped into the known rG4 patterns. The sequences able to form rG4 structure have certain motifs or patterns in eukaryotes (23). So, the sequences with this pattern might likely form rG4 structures. The RTS sites obtained were extended to 50 bases upstream according to their TUs, and then the extending sequences were used for pattern matching and categorized into 5 subclasses. The first class is G3L1–7, canonical rG4 with loop length between 1 and 7 nt [“(G_3+_N_1–7_)(G_3+_N_1__−__7_)(G_3+_N_1__−__7_) G_3+_” with N = A, U, C, or G]. The second class is long loop, rG4 with any loop of >7-nt length, up to 12 nt for lateral loop and 21 for the central loop (e.g., “G_3+_N_8–12_G_3+_N_1–7_G_3+_N_1–7_G_3+_” or “G_3+_N_1–7_G_3+_N_13–21_G_3+_N_1–7_G_3+_”). The third class is bulge, rG4 with a bulge of 1 to 7 nt in one G-tract or multiple 1-nt bulges [e.g., “G_3+_N_1–9_G_3+_N_1–9_(GGH_1–7_G|GH_1–7_GG) N_1–9_G_3+_” or “(GGHG|GHGG)N_1–9_ (GGHG|GHGG)N_1–9_G_3+_N_1–9_G_3+_” with H = A, U, or C]. The fourth class is 2-quartet, rG4 with 4 tracts of two consecutive G’s [“(G_2+_N_1–9_)(G_2+_N_1−9_)(G_2+_N_1−9_) G_2+_”]. The fifth class is rG4 sequences that are ≥40% G and do not fall into the four previous categories. The sequences not in any previous categories were put into the class “Others.” When matching multiple categories, the sequences are included in the class with higher predicted stability; the stability rank from high to low is canonical rG4s, long loops, bulges, and 2-quartets.

### Plasmid construction.

All the plasmids used in this study are listed in Table S2. The pMS402 plasmid was used to generate promoter-*lux* fusions or promoter-open reading frame (ORF)-*lux* fusions as described in a previous study (44). To verify the function of rG4 in the coding region, the G located in the third base of a codon was replaced with A to avoid amino acid mutation by overlap PCR. The rG4 sequences of *hemL* and *bswR* are shown in Fig. 3 and 5 and Fig. S3. The rG4 sites and their flanking sequences of *hemL* and *bswR* are listed in Table S2. The promoter region or promoter-ORF of rG4-located genes was generated by using the respective primers (Table S2). These promoters or promoter-ORF regions were cloned into the XhoI/BamHI sites upstream of the *luxCDABE* gene in pMS402. For the 5′ UTR insertion in the pMS402 plasmid, the wild-type rG4 and rG4 mutated sequences of *hemL* and *bswR* were inserted into its 5′ UTR DNA sequences. The synthetic nucleotide fragments (337 bp for *hemL* and 300 bp for *bswR*) were inserted between the XhoI/BamHI sites upstream of the *luxCDABE* gene in pMS402 accordingly. The 5′ UTR synthetic nucleotide fragments are listed in Table S2. All the plasmids were sequenced.

The overexpressed plasmid pAK1900 was constructed by amplifying the *hemL*/*bswR* ORF (or FLAG-tagged HemL/BswR) fragments with the primers pAK1900-*bswR*-F/R (Table S2) by PCR. The PCR fragments were cloned into pAK1900 (HindIII/BamHI) (45). For the 3′ UTR insertion plasmids, the rG4 sequences of *hemL* and *bswR* were inserted by pAK1900*-hemL*/*bswR*-F and pAK1900-*hemL*/*bswR*-(to 3′ UTR)-R primers, respectively (Table S2). All constructs were verified by sequencing, and the resultant vector was electroporated into Pseudomonas aeruginosa or Escherichia coli for the corresponding experiments.

### The *lux* reporter assay.

The procedures for the *lux* reporter assay are described in a previous study (46). Briefly, Pseudomonas aeruginosa and Escherichia coli containing pKD-WT-*lux* or pKD-ΔrG4-*lux* of the CDS or UTR were cultured overnight, and the overnight cultures were diluted to an OD_600_ of 0.6 for 2 h at 37°C with shaking at 220 rpm. The 5-μl cultures were transferred into 95 μl fresh LB broth in a black 96-well plate with a transparent bottom. The *lux* activity and bacterial growth were measured by OD_600_ in a Synergy 2 plate reader (BioTek) at the same time.

### Real-time quantitative PCR (RT-qPCR).

All the strains were cultured at 37°C and 220 rpm overnight in 2 ml LB supplemented with corresponding concentrations of carbenicillin (150 μg/ml for PAO1 and 60 μg/ml for K-12 MG1655) and inoculated into 2 ml fresh LB (1:100 dilution) until the OD_600_ reached 0.6. The cultures were centrifuged as pallets at 8,000 rpm for 1 min to harvest the bacteria. Total RNA was extracted by using a bacterial total RNA isolation kit (Sangon Biotech). RNA concentration was measured at 260 nm with a NanoDrop 2000 spectrophotometer (ThermoFisher). The total of 600 ng total RNAs was added into 20-μl reaction volumes to synthesize cDNA by using a FastKing RT kit (Tiangen Biotech). RT-qPCR was performed with a SuperReal Premix Plus (SYBR green) kit (Tiangen Biotech). Each reaction was performed in triplicate in 20-μl reaction volumes with 600 ng cDNA and *rspL* as an internal control. For each reaction, 200 nM primers (Table S2) were used for RT-qPCR. The reactions were run at 42°C for 15 min and 95°C for 3 min and kept at 4°C until used. The fold change represents relative expression level of mRNA, which can be estimated by the threshold cycle [2^−(ΔΔ^*^CT^*^)^] values. All the reactions were conducted with at least three repeats.

### Western blotting.

For Western blotting, the FLAG-tagged HemL and BswR were expressed using pAK1900*-hemL/bswR* (WT/ΔrG4) and pAK1900-*hemL/bswR* (WT/ΔrG4) (to the 3′ UTR) in wild-type strains with empty pAK1900 as the negative control. All the strains were cultured at 37°C and 220 rpm overnight in 2 ml LB supplemented with corresponding concentrations of carbenicillin (150 μg/ml for PAO1 and 60 μg/ml for K-12 MG1655). All tested *E. coli* and P. aeruginosa cultures were inoculated into 2 ml fresh LB (1:100 dilution) and continued in culture at 37°C and 220 rpm until OD_600_s reached 0.6 and 1.2, respectively. One-hundred-microliter cultures were centrifuged as pallets at 8,000 rpm for 1 min, and the supernatant was discarded to harvest the bacteria. The pellets were washed in 10 μl PBS, lysed by using sonication at 6-s intervals, and centrifuged at 4°C (12,000 rpm, 10 s). The total proteins were quantified using the Bradford protein assay kit (Tiangen Biotech). The same amounts of protein (50 μg for HemL and 100 μg for BswR) were loaded and separated by 12% SDS-PAGE. The proteins were transferred onto a polyvinylidene difluoride (PVDF) membrane (GE Healthcare Life Science) and hybridized with a mouse monoclonal FLAG antibody (1:10,000 dilutions; Sigma) and peroxidase-conjugated AffiniPure goat anti-mouse IgG(H+L) (Proteintech), respectively. The signal was detected with a high-sensitivity enhanced chemiluminescence (ECL) detection kit (Vazyme). Photographs were taken by using the Bio-Rad imaging system.

### Swarming motility assay.

The swarming motility assay was performed as described in a previous study with minor modification (47). The swarming motility assay consisted of 0.4% agar (MP Biomedical, USA), 8 g/liter nutrient broth mix (Beijing Land Bridge Technology, China), and 5 g/liter glucose (Sigma). Overnight LB cultures were inoculated on swarming plates as 2.5-μl aliquots, and the plates were incubated for 12 h at 37°C and then incubated at room temperature for an additional 12 h. Finally, photographs were taken by using the Bio-Rad imaging system and the diameter of motility trace was measured, representing the swarming motility of different bacterial strains.

### Measurement of biofilm production.

Biofilm production was detected as previously reported with minor modification (47). Briefly, overnight cultures were transferred into 2 ml LB broth (1:100 dilutions) supplemented with 100 μg/ml carbenicillin and cultured statically at 30°C for 16 h in in 10-ml borosilicate tubes, and the OD_600_ was measured. Crystal violet (0.1%) was used to stain biofilm adhering to the tubes, and unbound dye was washed with distilled water. The tubes were washed three times gently with sterilized water, and the residual crystal violet was dissolved in 1 ml of 95% ethanol with shaking. A 100-μl portion of this eluate was transferred to a transparent 96-well plate to measure the absorbance at 590 nm. The OD_590_/OD_600_ ratio represents the final biofilm production.

### CD assay.

Circular dichroism (CD) spectroscopy was performed using a Jasco J-1500 CD spectrophotometer, and a 1-cm-path-length quartz cuvette (Hellma Analytics) was employed in a volume of 2 ml. RNAs with 5 μM final concentrations were prepared in 10 mM lithium cacodylate (LiCac) (pH 7.0) and 150 mM KCl or LiCl (48). The mixtures were then mixed and heated at 95°C for 5 min (for denaturation) and cooled naturally to room temperature over 15 min (for renaturation). The RNAs were scanned from 220 to 310 nm at 25°C, and spectra were acquired every 1 nm. All spectra reported were averages from 2 scans with a response time of 2 s/nm (49, 50). They were then normalized to molar residue ellipticity and smoothed over 5 nm (51). All data were analyzed with Spectra Manager Suite (Jasco Software).

### QUMA-1 ligand-enhanced fluorescence assay.

A final concentration of 1 μM RNAs was prepared in 10 mM LiCac (pH 7.0) and 150 mM KCl or LiCl in a total reaction volume of 100 μl. The mixture was then denatured at 95°C for 5 min and cooled to room temperature for 15 min for renaturation to occur. A 1 μM final concentration of QUMA-1 ligand was added in a 1:1 ratio (RNA/QUMA-1). The fluorescence measurement was performed using a Horiba FluoroMax-4 spectrometer and a 1-cm-path-length quartz cuvette with a reaction volume of 100 μl. The sample was excited at 555 nm, and the emission spectrum was collected from 575 to 800 nm. Spectra were acquired every 2 nm at 25°C for both the wild-type and mutant rG4s. The entrance and exit slits were 5 and 2 nm, respectively.

### ThT ligand-enhanced fluorescence assay.

A final concentration of 1 μM RNAs was prepared in 10 mM LiCac buffer (pH 7.0) and 150 mM KCl or LiCl in a total reaction volume of 100 μl. The mixture was then denatured at 95°C for 5 min and cooled to room temperature for 15 min for renaturation to occur. A 1 μM final concentration of ThT ligand was added in a 1:1 ratio (RNA/ThT). The fluorescence measurement was performed using a Horiba FluoroMax-4 spectrometer and a 1-cm-path-length quartz cuvette with a reaction volume of 100 μl. The sample was excited at 425 nm, and the emission spectrum was collected from 440 to 700 nm. Spectra were acquired every 2 nm at 25°C for both the wild-type and mutant rG4s. The entrance and exit slits were 5 and 2 nm, respectively (52).

### Experimental data analyses.

Two-tailed Student’s *t* tests were performed using Microsoft Office Excel 2016. Significance was indicated as follows: *, *P* < 0.5; **, *P* < 0.01; ***, *P* < 0.001. Results represent means ± SD. All experiments were repeated at least three times.

### Data availability.

The rG4-seq data are available in the National Center for Biotechnology Information Gene Expression Omnibus under accession no. GSE129281.

10.1128/mBio.02926-19.1 TEXT S1 Supplemental references. Download Text S1, PDF file, 0.2 MB. Copyright © 2020 Shao et al. 2020 Shao et al. This content is distributed under the terms of the Creative Commons Attribution 4.0 International license.
