# Supplementary material for: RNA G-Quadruplex Structures Mediate Gene Regulation in Bacteria
Source: mBio. 2020 Jan 21;11(1):e02926-19. doi: 10.1128/mBio.02926-19 (PMC6974567; doi:10.1128/mBio.02926-19)
Supplement: TABLE S1 [file mBio.02926-19-st001.pdf]

Supplemental Table

Table S1A. Whole-genome location analysis of rG4 sites from rG4-seq *in E. coli.*

Position	Sequence_50bp	Sequence_90bp	Length	Class	POS	RTS	P value	Region	Start	End	Strand	Locus_tag	Name
269	GTGCGCAAAATTGA GGCACTGGCGGATG GCATTATGGATGCC GGGCTGGT	GTCGTTGCGGGTGCGCAAAATT GAGGCACTGGCGGATGGCATTA TGGATGCCGGGCTGGTATCGGT GCGTGAACAGGCGCGTCCAGCG GC	50	2 quartet	3761639	0.447917	0.00013	CDS	3761347	3761955	-	b3592	*yibF*
1718	CCTGGTCGCAGGCA ATGGTGACTCTGGC GCTGGTGTTAACCG CCCTGCTG	GCAATCGGTGCCTGGTCGCAGG CAATGGTGACTCTGGCGCTGGT GTTAACCGCCCTGCTGTTCTGTA TCGTCATCGGTTTGCCGTTGGGG	50	2 quartet	2806479	0.276786	0.000133	CDS	2806010	2807074	+	b2678	*proW*
608	AAAGCGGCGCGTGG CTATCATCTGGCGC AGGGCAATCCGGCG CGTGAAAT	CAGCCTGAGCAAAGCGGCGCGT GGCTATCATCTGGCGCAGGGCA ATCCGGCGCGTGAAATCAAACC GACCACCATTTTGCATGTTGCGG C	50	2 quartet	3200346	0.482143	0.000615	CDS	3199664	3200965	-	b3054	*ygiF*
78	GCTGGAACGCATTA ATCTCGATATCCCC GGCGCGGTGGCCCA GGCGCTGC	GTGACGAGCTGCTGGAACGCAT TAATCTCGATATCCCCGGCGCG GTGGCCCAGGCGCTGCGGGAAG ATTTAGGCGGAACAGTCGATGC CA	50	2 quartet	118552	0.473684	0.001067	CDS	117752	118645	-	b0109	*nadC*
616	CTTCGAAAAATCGG ATAAGGAACAGTGG	TGAAACAGCACTTCGAAAAATC GGATAAGGAACAGTGGGGATGG	50	2 quartet	3308309	0.318602	0.00109	CDS	3308040	3308924	-	b3163	*nlpI*

	GGATGGAACATTGTCGAGTTCT	AACATTGTCGAGTTCTACCTGGGCAACATTAGCGAACAAACGTTAA											
1791	TTCCTCTTCGGCGCGAAAGCGGCACCGGGCTACTACCTGGCGAAGAATAT	ACCGCGCGTCTTCCTCTTCGGCGCGAAAGCGGCACCGGGCTACTACCTGGCGAAGAATATTATCTTTGCGATCAACAAAGTGGCTGACGT	50	2 quartet	3550720	0.394767	0.001144	CDS	3550080	3552473	-	b3417	*malP*
551	AAATGAAAACTGACTATCAAGCGGTGGTCAGCGGGATAGCAGAAGGTTAC	CCGCCGTATGAAATGAAAACTGACTATCAAGCGGTGGTCAGCGGGATAGCAGAAGGTTACAAACGTTTCGCCACTGGTATTTACGCACTG	50	2 quartet	3645935	0.673077	0.001181	CDS	3645385	3646227	+	b3499	*rlmJ*
726	CGCATCCCCGGAATTTTGGACCGCAGTCGGTTCTGCACCGGAAAATTTTT	TCCGCGACTTCGCATCCCCGGAATTTTGGACCGCAGTCGGTTCTGCACCGGAAAATTTTTCTCACCTGACCGTGATGAATTTCATCACTG	50	2 quartet	953740	0.679965	0.001972	CDS	953609	954466	-	b0904	*focA*
578	CTTCCAGTCAGGGATCCCGGTGGTGATGGCCGGTCTGGATGTTACTCATA	CAGAAATTGTCTTCCAGTCAGGGATCCCGGTGGTGATGGCCGGTCTGGATGTTACTCATAAAGCACAAATCCACGTTGAAGACACCGAGC	50	2 quartet	683833	0.789366	0.00205	CDS	683477	684412	-	b0651	*rihA*
717	TGGAAGGTGGCGAGCTGCCGGATACTCATCTTGGCCGTTTACTGCAATTT	GTTGGTGTTCTGGAAGGTGGCGAGCTGCCGGATACTCATCTTGGCCGTTTACTGCAATTTAAAAAGTGGGGGTTGCCGGTCAGCGATCGG	50	2 quartet	2529439	0.475149	0.00207	CDS	2528161	2530176	-	b2411	*ligA*
2960	CATTCTTGTTGGTCA	TTAAGTGGCACATTCTTGTTGGT	50	2 quartet	4021814	0.307684	0.002411	CDS	4020226	4021866	+	b3835	*ubiB*

	GCCGACCTGAATGGGGGCTGATGCCCGGCTGGTTA	CAGCCGACCTGAATGGGGGCTGATGCCCGGCTGGTTAATGGCAGGTGGTCTGATCGCCTGGTTTGTC											
1283	GACGAGATGTCCGTGGTTGACGGCGAAGGCCGCGTACACGGGTTAGAAGG	AATGGGTTACGACGAGATGTCCGTGGTTGACGGCGAAGGCCGCGTACACGGGTTAGAAGGCCTGCGTGTGGTGGATGCGTCGATTATGCC	50	G ≥ 40 %	325752	0.604895	0.002459	CDS	325577	327247	-	b0311	*betA*
421	GCGCCGACTGCTACCTCGATATTCAGGCGGGGTCTGGCGGTACGGAAGCA	GAATATGACAGCGCCGACTGCTACCTCGATATTCAGGCGGGGTCTGGCGGTACGGAAGCACAGGACTGGGCGAGCATGCTTGAGCGTATG	50	2 quartet	3035859	0.675159	0.002639	CDS	3035184	3036282	-	b2891	*prfB*
1726	CAGGCAATGGTGACTCTGGCGCTGGTGTTAACCGCCCTGCTGTTCTGTAT	TGCCTGGTCGCAGGCAATGGTGACTCTGGCGCTGGTGTTAACCGCCCTGCTGTTCTGTATCGTCATCGGTTTGCCGTTGGGGATATGGCT	50	2 quartet	2806487	0.266208	0.002725	CDS	2806010	2807074	+	b2678	*proW*
469	GCTGACCATGACCGAAGAAGCCGGTATGGACGGTGCGTTCGGCTTACAGG	TGGAAGTGCTGCTGACCATGACCGAAGAAGCCGGTATGGACGGTGCGTTCGGCTTACAGGGCAACTGGTTGCAGGCTGATATTCTGATTA	50	2 quartet	255248	0.975543	0.002778	CDS	254259	255716	-	b0237	*pepD*
260	TCGTGGCGGCGGTAAGCTGGCCCCGGAAAACACCCTGGCGTCAATCGACG	TCGTCGCTCATCGTGGCGGCGGTAAGCTGGCCCCGGAAAACACCCTGGCGTCAATCGACGTCGGGGCAAAATACGGTCATAAGATGATC	50	2 quartet	3588029	0.466667	0.002919	CDS	3587370	3588113	-	b3449	*ugpQ*

		G											
698	GGCTAACCGTGCTGGCGCTCAGGAGTTGCGGGTTGTGGTTGAGCACGATC	TGCAAAGTATGGCTAACCGTGCTGGCGCTCAGGAGTTGCGGGTTGTGGTTGAGCACGATCCGGTTTTCGGGCCGTTGATCATGCTGGGTG	50	2 quartet	2721728	0.362669	0.00294	CDS	2719953	2722613	+	b2584	*pka*
105	GCCCCATGGATGTTGCGCTGGACATTGGTCCAGGTCTGGCGAAAGCCTGT	CACGCTGTAAGCCCCATGGATGTTGCGCTGGACATTGGTCCAGGTCTGGCGAAAGCCTGTATCGCAGGGCGCGTTAATGGCGAACTGGTT	50	2 quartet	1802466	0.482257	0.003347	CDS	1800642	1802570	-	b1719	*thrS*
274	AACATGTGCTGATTATCGGCGGCGGCGACGGTGCCATGCTGCGTGAAGTA	GGTCACGCGAAACATGTGCTGATTATCGGCGGCGGCGACGGTGCCATGCTGCGTGAAGTAACCCGACATAAAAACGTTGAGTCAATCACG	50	2 quartet	136180	0.571429	0.003411	CDS	135598	136464	-	b0121	*speE*
1722	GTCGCAGGCAATGGTGACTCTGGCGCTGGTGTTAACCGCCCTGCTGTTCT	TCGGTGCCTGGTCGCAGGCAATGGTGACTCTGGCGCTGGTGTTAACCGCCCTGCTGTTCTGTATCGTCATCGGTTTGCCGTTGGGGATAT	50	2 quartet	2806483	0.271117	0.003488	CDS	2806010	2807074	+	b2678	*proW*
3103	AACCGGTATTCTGGGGCCTCTTCGGGGCCGGTGGTATGTGGAGCGCCATC	CGTTCTGACGAACCGGTATTCTGGGGCCTCTTCGGGGCCGGTGGTATGTGGAGCGCCATCATTGCGCCGGTGATGATCCTGCTGGTGGGT	50	bulges	4379286	0.847368	0.003535	CDS	4379007	4379366	-	b4151	*frdD*

39	ATCCATAAGAAAGGTCAGGCACACTGGGAAGGCGATATC	ATCCATAAGAAAGGTCAGGCACACTGGGAAGGCGATATCAAACGCGGGAAGGGAACAGTATCCACCGAG	39	2 quartet	1556668	0.847537	0.003571	CDS	1556625	1557056	+	b1482	*osmC*
1623	CTGCCTTCTTCGGGCAGAACGGTGCGCTGGCGGTCTTCTCGCTGTATATG	GTATTCGCGGCTGCCTTCTTCGGGCAGAACGGTGCGCTGGCGGTCTTCTCGCTGTATATGCTGGGTATTGTGATGGCGGTGCTGACTGGC	50	2 quartet	3541785	0.514718	0.003695	CDS	3540407	3542728	+	b3409	*feoB*
1308	AGGGCAGGGTGCCAGGTAACGCCTGGGGGGGAAACCCACGACCAGTGCAA	CGGGCTCCATAGGGCAGGGTGCCAGGTAACGCCTGGGGGGGAAACCCACGACCAGTGCAACAGAGAGCAAACCGCCGATGGCCCGCGCAA	50	long loops	3270463	0.465957	0.003955	ncRNA_CDS	3270216	3270592	-	b3123	*rnpB*
388	TGGCGGGACGTACGGGCTGTGGCGTATGCGGCGTGGAGCAACTTAATGAC	CGCCGGGCGCTGGCGGGACGTACGGGCTGTGGCGTATGCGGCGTGGAGCAACTTAATGACATCGGAAAACCGGTGCAGCCGCTACCGTTC	50	2 quartet	4086407	0.35	0.004119	CDS	4086016	4086849	+	b3895	*fdhD*
2336	GCTTCACCCTGGCGGCGCTGGCGTCTACCGGTCGTGACATCAACTGGGAT	GACGCGCTGCGCTTCACCCTGGCGGCGCTGGCGTCTACCGGTCGTGACATCAACTGGGATATGAAGCGTCTGGAAGGTTACCGTAACTTC	50	2 quartet	4481945	0.651524	0.004542	CDS	4480982	4483837	-	b4258	*valS*
2699	GGCTCCGGTGGGTTTGTGTGGCATGTATGCGCGTCGTGGCGA	CGGTGGCACTGGCTCCGGTGGGTTTGTGTGGCATGTATGCGCGTCGTGGCGAAGTTCAGGCAGCCAA	50	2 quartet	3780099	0.264559	0.004637	CDS	3779827	3781017	+	b3605	*lldD*

	AGTTCAGG	AGCGGCGGACGCGCATGGTATTC											
39	TAGGCGCAGGCCAGGTGGCGGATAAGGTTCATGCTTCGT	TAGGCGCAGGCCAGGTGGCGGATAAGGTTCATGCTTCGTACTACTGCACCCGCAACGATCTGGAACTGG	39	2 quartet	1376912	0.868421	0.004747	CDS	1376832	1377887	+	b1315	*ycjS*
837	TGGTAAGCGGTATTCTGCCGGTTGTAGGGGTTCCGCTCCCACTGGTCAGT	AATATTGGTATGGTAAGCGGTATTCTGCCGGTTGTAGGGGTTCCGCTCCCACTGGTCAGTTATGGAGGATCGGCGCTAATTGTGCTGATG	50	2 quartet	665294	0.654605	0.004948	CDS	665201	666313	-	b0634	*mrdB*
1084	TGGCAGGCCTGGAGGGCATACGCCCGCTGGCCGCGAAACTTTCACCTGCA	AATCTGGAGCTGGCAGGCCTGGAGGGCATACGCCCGCTGGCCGCGAAACTTTCACCTGCACTGGGTGATGTTTGGCGCTCCACACAACCG	50	2 quartet	3395173	0.44	0.004995	CDS	3392458	3396258	-	b4472	*yhdP*
1387	TCGATGCCGAATTTGCTGTACTGGTTCGCTCGGATCTCAAAGGGTTAGGC	CCTGATAACATCGATGCCGAATTTGCTGTACTGGTTCGCTCGGATCTCAAAGGGTTAGGCTTAGGTCGACGCTTAATGGAAAAGTTGATT	50	2 quartet	2722417	0.496154	0.005018	CDS	2719953	2722613	+	b2584	*pka*
1724	TTGAGCCGCTCACTGCGCTGGTTGCGGCAGACAGTGGAATGGCAGACATC	GATGTCCGCTTTGAGCCGCTCACTGCGCTGGTTGCGGCAGACAGTGGAATGGCAGACATCGTGCATATCATCGAACAGTCGCGTAACGCG	50	2 quartet	1266743	0.52381	0.005217	CDS	1266094	1266927	+	b1212	*prmC*

657	AGGTCAGCGGCCTGAGTGAACCGGTGGTTGGCACAGGTTCAAGCCGTCGT	ATCCACTGCCAGGTCAGCGGCCTGAGTGAACCGGTGGTTGGCACAGGTTCAAGCCGTCGTAAGGCTGAGCAGGCTGCCGCCGAACAGGCG	50	2 quartet	2703437	0.496835	0.005292	CDS	2703383	2704063	-	b2567	*rnc*
1010	CCGAAAAACGGGCGCGTGCTGGCGGTTATCGGTATGCTACATGATAAAGA	GAAAGCGCTACCGAAAAACGGGCGCGTGCTGGCGGTTATCGGTATGCTACATGATAAAGATATTGCCGGAACTCTGGCCTGGTTGAAAAG	50	2 quartet	2431933	0.81625	0.005608	CDS	2431674	2432942	-	b2315	*folC*
633	GGGATACTGGATAAGGGTATTAGGCATGCAGAAAGAACAACTTTCCGCTT	TGACGATAGCGGGATACTGGATAAAGGGTATTAGGCATGCAGAAAGAACAACTTTCCGCTTTAATGGATGGCGAAACGCTGGATAGTGAGC	50	2 quartet	2709380	0.964286	0.006199	CDS	2708754	2709404	-	b2572	*rseA*
1288	ATGACCCGGCAAGCTATGTGGAGGTGGAAAAAGGCCAGTTGACCTTCCGT	GGTCGCAAAAATGACCCGGCAAGCTATGTGGAGGTGGAAAAAGGCCAGTTGACCTTCCGTAATGCCGCCGATCTTTATCTCTATCCCAAT	50	2 quartet	4435270	0.304545	0.006244	CDS	4434622	4436565	-	b4213	*cpdB*
1592	GTCACGCTGTTTACCAAAGGCGGCGGACAGTGGCTGGAAGCCATGGCAGA	CCGCGTACCGGTCACGCTGTTTACCAAAGGCGGCGGACAGTGGCTGGAAGCCATGGCAGAAACCGGTTGCGATGCGCTGGGCCTCGACTG	50	2 quartet	4198496	0.61383	0.006316	CDS	4197716	4198780	+	b3997	*hemE*
1354	CGTGGGGATCTGGGGCGGATCTCTGGTC	TTTTCGTCGCCGTGGGGATCTGGGGCGGATCTCTGGTCGGCGTCA	50	2 quartet	3338708	0.641667	0.007123	CDS	3338466	3339248	-	b3194	*mlaE*

	GGCGTCAGTTGGAAAGGCATTG	GTTGGAAAGGCATTGATAGCGGGTTCTTCTGGTCGGCAATGCAAA												
651	CGGCAGGGGCTTACTGGCGTGGCGACAGCAACAACAAAATGTTGCAACGT	CTAATGAAAACGGCAGGGGCTTACTGGCGTGGCGACAGCAACAACAAAATGTTGCAACGTATTTACGGTACGGCGTGGGCAGACAAAAAA	50	2 quartet	1801920	0.404234	0.007152	CDS	1800642	1802570	-	b1719	*thrS*
1419	CCGTGCAGGTGGCGCGCCGACGCTGGCGGTAGGTATCGCGCACGTGTTCC	CTGTCCTGAACCGTGCAGGTGGCGCGCCGACGCTGGCGGTAGGTATCGCGCACGTGTTCCACAAAGTGCTGCCGATGGCTGACATGGGCT	50	G ≥ 40 %	4589848	0.484973	0.007488	CDS	4589129	4591279	-	b4354	*yjiY*
1868	GGCGGCGACGGCAACTACGGTTACAACGCAGCAACCGAAGAATACGGCAA	CACCGTTAAAGGCGGCGACGGCAACTACGGTTACAACGCAGCAACCGAAGAATACGGCAACATGATCGACATGGGTATCCTGGATCCAAC	50	2 quartet	4372483	0.26906	0.008399	CDS	4371025	4372671	+	b4143	*groL*
2645	ATGAATCCGGTATCGTTTACATTGGTGCGGAAGTGACCGGTGGCGACATT	TCCAAACTGGATGAATCCGGTATCGTTTACATTGGTGCGGAAGTGACCGGTGGCGACATTCTGGTTGGTAAGGTAACGCCGAAAGGTGAA	50	2 quartet	4183889	0.261309	0.008469	CDS	4181245	4185273	+	b3987	*rpoB*
899	TCCGACATCTCCGAGCGCGGCATGGTGCTCACCGGTGGTGGCGCACTGCT	GGAACTGGCTTCCGACATCTCCGAGCGCGGCATGGTGCTCACCGGTGGTGGCGCACTGCTGCGTAACCTTGACCGTTTGTTAATGGAAG	50	2 quartet	3400189	0.280972	0.009027	CDS	3400044	3401087	-	b3251	*mreB*

		A											
499	AAACCGGCGAAAGTTGCGGCATTGATGGCGCAGTGGCTGGTTAATGGCTG	TATGGTTGAAAAACCGGCGAAAGTTGCGGCATTGATGGCGCAGTGGCTGGTTAATGGCTGGTGCCGTGAAACCATTTTCAACCTCAAACT	50	2 quartet	2940354	0.426643	0.009184	CDS	2940143	2941243	-	b2806	*rlmM*
188	GATTATTTTGGTGTGGAATTAGTGGTGCGGAAAAATACCGGTGTAACATT	CGATATTGAAGATTATTTTGGTGTGGAATTAGTGGTGCGGAAAAATACCGGTGTAACATTAACACCTGCCGGTCAATTGTTACTCTCCCG	50	2 quartet	3266878	0.379085	0.009373	CDS	3266127	3267065	-	b3118	*tdcA*
694	TGGCTTACGGTCTGGACAAAGGCACTGGCAACCGTACTATCGCGGTTTAT	GCAGCTGCGCTGGCTTACGGTCTGGACAAAGGCACTGGCAACCGTACTATCGCGGTTTATGACCTGGGTGGTGGTACTTTCGATATTTCT	50	2 quartet	12740	0.32258	0.009691	CDS	12163	14079	+	b0014	*dnaK*
5328	GGCCTGGCGGTGGACTTTGAAATCGACGGTGAATATCCGCAGTACGGCAA	TGACGAAAACGGCCTGGCGGTGGACTTTGAAATCGACGGTGAATATCCGCAGTACGGCAACAACGACGAGCGCGTAGACAGCATTGCCTG	50	2 quartet	3260701	0.65	0.00976	CDS	3260124	3262418	-	b3114	*tdcE*
308	GGGGCTGCTGGCTGGACAACTCTCGGCAGCCGAGGTGGCAATATGGTCGA	GATATCTATCGGGGCTGCTGGCTGGACAACTCTCGGCAGCCGAGGTGGCAATATGGTCGATCAGGACTGGATGGATTCCAGTAACCCCGG	50	2 quartet	585326	0.70902	0.00977	CDS	584680	585633	-	b0565	*ompT*
1100	GGACTGGTCTGCGCCCGATGACGCCAGA	GCGACTTTCTGGACTGGTCTGCGCCCGATGACGCCAGACGGCACG	50	G ≥ 40 %	1238670	0.513158	0.009803	CDS	1237571	1238869	+	b1189	*dadA*

	CGGCACGCCGGTTG TCGGGCGT	CCGGTTGTCGGGCGTACACGCTT TAAAAATCTGTGGCTGAATACC											
135	CAAAAACCACGTTG ATATTGCTCGCACT GGGCGAAGGTGGCG GAATTGGT	CCCCTCGCACCAAAAACCACGT TGATATTGCTCGCACTGGGCGA AGGTGGCGGAATTGGTAGACGC GCTAGCTTCAGGTGTTAGTGTTC T	50	2 quartet	4606267	0.332837	0.01022	tRNA_ CDS	4606200	4606286	-	b4369	*leuP*
2435	GTAACCAGGCGCGT GTGGCGGACGGTGC AACGGTGGTTTCCA CCTCTACC	CTGTGTATGGGTAACCAGGCGC GTGTGGCGGACGGTGCAACGGT GGTTTCCACCTCTACCCGTAACT TCCCGAACCGTCTGGGTACTGG C	50	2 quartet	133983	0.636075	0.010647	CDS	131615	134212	+	b0118	*acnB*
2035	CGGTGCTCATGCGG CAAGCTGGTTTATC AATGGCGGCAAAAC ACCACTCA	GGATGATGGCCGGTGCTCATGC GGCAAGCTGGTTTATCAATGGC GGCAAAACACCACTCAAATTTG GCGCGATTAGCGACTGGATGGA AG	50	2 quartet	323651	0.347682	0.010978	CDS	322338	323765	+	b0307	*ykgF*
1398	CTTGTCGGCCTGGC GGTGGCACTTTATT CCGGCATCAACTGG ATGGGTAA	GACTGTAGGGCTTGTCGGCCTG GCGGTGGCACTTTATTCCGGCAT CAACTGGATGGGTAACCTGCGT GAAGCGATTCGTGCCCAGTCGC G	50	2 quartet	3673824	0.525207	0.011591	CDS	3673362	3674375	+	b3522	*yhjD*
833	CAGGCATGTTGGAC GGGGGGCCGAAAAT TACTCTACCTGGCG ATGACACG	TTGCAACGCACAGGCATGTTGG ACGGGGGGCCGAAAATTACTCT ACCTGGCGATGACACGCCAACT GACGCGGTAGTCAGCCCATCCG	50	2 quartet	981879	0.390977	0.011641	CDS	981047	982894	+	b0925	*ldtD*

		CT											
329	GAAAGCGGCTGGGTAGGCTTTGTGGAAGCTGCAATTTTTATTTTTGTGTT	CTCTATCCGCGAAAGCGGCTGGGTAGGCTTTGTGGAAGCTGCAATTTTTATTTTTGTGTTACTGGCAGGTCTGGTTTATCTGGTGCGTAT	50	2 quartet	2404744	0.516525	0.011735	CDS	2404629	2405072	-	b2288	*nuoA*
818	CCAGAACTGGCTTACGGCAAAGCGGGTGTTCCGGGGATCCCACCGAATTC	GGTTATTCCACCAGAACTGGCTTACGGCAAAGCGGGTGTTCCGGGGATCCCACCGAATTCTACCCTGGTGTTTGACGTAGAGCTGCTGGA	50	2 quartet	3476710	0.303453	0.011743	CDS	3476607	3477419	-	b3347	*fkpA*
897	GCCGCCTGCGTATGGAAGGCAAGCGCGTGGCGCTGGTGCCTACCATGGGT	CAGCAAATTCGCCGCCTGCGTATGGAAGGCAAGCGCGTGGCGCTGGTGCCTACCATGGGTAACCTGCACGATGGCCATATGAAGCTGGTC	50	2 quartet	148703	0.304253	0.012155	CDS	147944	148795	-	b0133	*panC*
2397	GTCGGAACTGGATATGATGGTCGGGAAAATCCTCTGTTATCTCTATCTCA	GTTCCGTCTTGTCGGAACTGGATATGATGGTCGGGAAAATCCTCTGTTATCTCTATCTCAGCCCGGAACGGCTGGCGAATGAGGGGATTT	50	2 quartet	975933	0.533333	0.012283	CDS	975622	976326	+	b0923	*mukE*
1471	GAGCAAGGCCAAATAGGGGTTCATAAGGTACGGCCCGTACTGAACCCGGG	ACTCCACCCGGAGCAAGGCCAAATAGGGGTTCATAAGGTACGGCCCGTACTGAACCCGGGTAGGCTGCTTGAGCCAGTGAGCGATTGCTG	50	2 quartet	3270300	0.507499	0.012508	ncRNA_CDS	3270216	3270592	-	b3123	*rnpB*
147	GCGGGCTGCTGGCAAAAGTGCGCGACGGGGACATCATTCGTG	GCCTACGATGGCGGGCTGCTGGCAAAAGTGCGCGACGGGGACATCATTCGTGTGAATGGACAGACA	50	G ≥ 40 %	1932961	0.302151	0.012719	CDS	1932793	1934604	-	b1851	*edd*

	TGAATGGA	GGCGAACTGACGCTGCTGGTAGAC												
717	CTGGATGCTGGCACGGATGTCCGGCGCATGTCTGGTGCCCTTCGTTCCAC	CGACCGGAACCTGGATGCTGGCACGGATGTCCGGCGCATGTCTGGTGCCCTTCGTTCCACGCCGTAAGCCAGATGGCAAAGGGTATCAAT	50	2 quartet	1115859	0.909091	0.013285	CDS	1115662	1116582	-	b1054	*lpxL*
430	AGGTCCGCGCGGTTCGCTGGTGGTGCCGGAAGATTACGCGTATCAGCTGT	TTACGGTGGCAGGTCCGCGCGGTTCGCTGGTGGTGCCGGAAGATTACGCGTATCAGCTGTATGTCTGCGATGAATCCGGAATGCCTGCAT	50	2 quartet	3216062	0.371816	0.01409	CDS	3215727	3216491	-	b3070	*yqjH*
2612	ACCGTCTGCGTCGTTTAGGCATGGTGTGGTTTATGGGCCACGACAGCAGC	TCTTCGCTCAACCGTCTGCGTCGTTTAGGCATGGTGTGGTTTATGGGCCACGACAGCAGCAAGTTTCGCATTACCGAATCGGTGTTCCGC	50	2 quartet	976148	0.425805	0.014194	CDS	975622	976326	+	b0923	*mukE*
366	TCGCTATGGCACTGGGCGGAACGGCGTTAGTGTTCTTCTGCTGCTCTGCA	GGTGACGTAATCGCTATGGCACTGGGCGGAACGGCGTTAGTGTTCTTCTGCTGCTCTGCATATGTGCTGACCACCCGCAAAGATATGTCG	50	2 quartet	1031053	0.448046	0.014304	CDS	1030759	1031418	-	b0970	*yccA*
1425	GAAACGTGCATTTATTATGGTGCTGGACTCATTCGGCATCGGCGCTACAG	GAGAACATATGAAACGTGCATTTATTATGGTGCTGGACTCATTCGGCATCGGCGCTACAGAAGATGCAGAACGCTTTGGTGACGTCGGGG	50	2 quartet	4619653	0.343485	0.014467	CDS	4619603	4620826	+	b4383	*deoB*

609	CTGCACGCCGTTCTAACGGCGACGGTGTTGGCGGTTCTATCAGCTACGAA	GAGCGTGACACTGCACGCCGTTCTAACGGCGACGGTGTTGGCGGTTCTATCAGCTACGAATACGAAGGCTTTGGTATCGTTGGTGCTTAT	50	2 quartet	986374	0.374124	0.014468	CDS	985894	986982	-	b0929	*ompF*
80	CGGTGATTAAAGTCATCGGCGTCGGCGGCGGCGGTAATGCTGTTGAA	ACCAATGACGCGGTGATTAAAGTCATCGGCGTCGGCGGCGGCGGTAATGCTGTTGAACACATGGTGCGCGAGCGCATTGAAGGTGTT	50	2 quartet	105384	0.307769	0.014641	CDS	105305	106456	+	b0095	*ftsZ*
125	AAGAGATCGCGCAGCTGGAAGTCACCGGCGAATCTGGCGCAGGTCTGGTA	AAAATGCAGGAAGAGATCGCGCAGCTGGAAGTCACCGGCGAATCTGGCGCAGGTCTGGTAAAAGTGACCATCAACGGTGCACACAACTGC	50	2 quartet	494200	0.508284	0.01512	CDS	494076	494405	+	b0471	*ybaB*
427	GGCTCGGTGAAGGGCTACGCGGGTGACACCGCCACCACCAGTGAAATCAA	CGCTAAAGAAGGCTCGGTGAAGGGCTACGCGGGTGACACCGCCACCACCAGTGAAATCAAAGCCAAACTGCTGGCGGACGATATCGTCCC	50	2 quartet	4611822	0.273116	0.015171	CDS	4611396	4612001	+	b4376	*osmY*
103	GGCACTGGTAGGCTGCGGTCAGGATGAAAAAGATCCAAACCACATTAAAG	TCGGATCACTGGCACTGGTAGGCTGCGGTCAGGATGAAAAAGATCCAAACCACATTAAAGTCGGCGTGATTGTTGGTGCCGAACAGCAGG	50	2 quartet	220826	0.534444	0.015599	CDS	220113	220928	-	b0197	*metQ*
1995	CGGCGTTGGCGACA	GGTGCCGCTACGGCGTTGGCGA	50	2 quartet	3496005	0.592439	0.016032	CDS	3494011	3496554	+	b3365	*nirB*

	GCGTCGGCCTCGGC GTGGAACTGGAAAA CCGCTACA	CAGCGTCGGCCTCGGCGTGGAA CTGGAAAACCGCTACAAAGGCA TCCGTACGCCGCACAAAATGAA GT											
1512	GGCTGGCAAAACTG GCACTGGAAGAGCT GCCGCCGGGCTGGT TCTATAGC	GAAGCGGAACGGCTGGCAAAAC TGGCACTGGAAGAGCTGCCGCC GGGCTGGTTCTATAGCCGCATTG TGGCAACCTCGGTGCTGGGTGA A	50	2 quartet	3554541	0.588819	0.016359	CDS	3553085	3555790	+	b3418	*malT*
221	TGTTCGGTGCGGCA GTCGGTGCGGTGGG CAGCGGCTGGCTCT CCTTTAAA	AGCTCCATGATGTTCGGTGCGG CAGTCGGTGCGGTGGGCAGCGG CTGGCTCTCCTTTAAACTCGGGC GCAAAAAGAGCCTGATGATCGG C	50	2 quartet	3088516	0.786111	0.016459	CDS	3088284	3089678	+	b2943	*galP*
196	TATCGCGCTTGGGG CAGGTGGTCTGCCG ATGGGCCGTATCGT CGAAATCT	TTTCACTGGATATCGCGCTTGGG GCAGGTGGTCTGCCGATGGGCC GTATCGTCGAAATCTACGGACC GGAATCTTCCGGTAAAACCACG C	50	2 quartet	2823574	0.59688	0.0165	CDS	2822708	2823769	-	b2699	*recA*
2776	TACGAACGCTTTAC CTCTATCGGCCCGC TGATGGAGAAAATC GGTAATGG	TCCGGCGACTTACGAACGCTTTA CCTCTATCGGCCCGCTGATGGA GAAAATCGGTAATGGCGGTAAA GGGATTGCCTGGAACACCCAGA G	50	2 quartet	1282600	0.298856	0.016558	CDS	1279864	1283607	+	b1224	*narG*
407	ACTAACGCAATCAA AGAGCGCGTGCTGG	TCCGCACATCACTAACGCAATC AAAGAGCGCGTGCTGGAAGGTG	50	2 quartet	2909260	0.744584	0.016665	CDS	2908029	2909666	-	b2780	*pyrG*

	AAGGTGGCGAAGGTCATGACGT	GCGAAGGTCATGACGTAGTACTGGTAGAAATCGGCGGTACAGTAGG											
153	CCTGGTGGCTGGTTGCTGGACCGTTTTGGTTCAAAACGCGTCTACTTCTG	CGGGCAGATCCCTGGTGGCTGGTTGCTGGACCGTTTTGGTTCAAAACGCGTCTACTTCTGGTCGATCTTTATCTGGTCGATGTTTACCTT	50	2 quartet	2921832	0.844444	0.017028	CDS	2920748	2922100	-	b2789	*gudP*
710	CTGGATAGCGGCCTGGTGGTGATCCCGAAATCGGTCACACCTTCACGTAT	CCGCTGGCATCTGGATAGCGGCCTGGTGGTGATCCCGAAATCGGTCACACCTTCACGTATTGCCGAAAACTTTGATGTCTGGGATTTCCG	50	2 quartet	3157334	0.659259	0.017158	CDS	3156623	3157450	+	b3012	*dkgA*
845	ATGGATGCGCTGGCCCCGACGGGTCCGGTCTATCAGGCGGGTACGCTTTC	CCTCGGCAAAATCATCGGCGGTGGAATGCCGGTAGGCGCATTCGGTGGTCGTCGTGATGTAATGGATGCGCTGGCCCCGACGGGTCCGGT	50	2 quartet	174038	0.355081	0.017548	CDS	173602	174882	-	b0154	*hemL*
509	GTACCGGTGATCCTCGGCGGTATTGAGGCTAGTCTGCGCCGTACCGCGCA	GTGGAAAGATGTACCGGTGATCCTCGGCGGTATTGAGGCTAGTCTGCGCCGTACCGCGCATTATGATTACTGGTCCGATACCGTGCGCCG	50	2 quartet	3160635	0.687087	0.017659	CDS	3158927	3161146	-	b4469	*ygiQ*
1683	TTTGCGGTGGAAGATACGCAGAAATACGGCCAGGCGATTGGTCACATCGG	TAACTTCTCCTTTGCGGTGGAAGATACGCAGAAATACGGCCAGGCGATTGGTCACATCGGTAAACTTGCTGCGGGTTCTCTGAAAGTGGG	50	2 quartet	2820408	0.435993	0.017795	CDS	2819381	2822011	-	b2697	*alaS*
809	ATGACTTGTGGCTG	AAATTAGCGGATGACTTGTGGC	50	2 quartet	226567	0.738571	0.018787	rRNA_	225759	228662	+	b0204	*rrlH*

	GGGGTGAAAGGCCAATCAAACCGGGAGATAGCTGGT	TGGGGGTGAAAGGCCAATCAAACCGGGAGATAGCTGGTTCTCCCCGAAAGCTATTTAGGTAGCGCCTC						CDS					
1878	GCATTGCTGTGGGCTCAACCGGAAATCTGGGGTTATCAATCGGCATTATG	AGCCAATACAGCATTGCTGTGGGCTCAACCGGAAATCTGGGGTTATCAATCGGCATTATGAGCGCCCGCATTGGCTTTAAGGTGACAGTT	50	2 quartet	2479734	0.311111	0.019971	CDS	2479202	2480530	+	b2366	*dsdA*
1281	GCGTTTACCGCTGGCCGAACAAGGTCTGGCGTGGTTAATGCCAACAGTGG	CCTGGGCGCAGCGTTTACCGCTGGCCGAACAAGGTCTGGCGTGGTTAATGCCAACAGTGGTGATGGTGGTTCTGGCCATTATCTGGGATC	50	2 quartet	420841	0.410112	0.020148	CDS	419591	420910	+	b0401	*brnQ*
1569	GTGCGGCGGTAGGCCTGGTGGGCAAAGAGTCTGATTTGTTCCGCTTTACT	GCTATCGCCTGTGCGGCGGTAGGCCTGGTGGGCAAAGAGTCTGATTTGTTCCGCTTTACTGTCAAACACAGCCTGATCTTCACCTGTATA	50	2 quartet	3778969	0.339226	0.020169	CDS	3777399	3779054	+	b3603	*lldP*
783	CGGAATTCTGGATGCTGGAGCCGGAAGTGGCGTTTGCTAACCTGAACGAT	CGTCACCTGGCGGAATTCTGGATGCTGGAGCCGGAAGTGGCGTTTGCTAACCTGAACGATATTGCGGGTCTGGCTGAAGCCATGCTGAAA	50	2 quartet	988203	0.459683	0.020229	CDS	987585	988985	-	b0930	*asnS*
1664	GCCGCCACGGTTATCAGGAAGGTATCGCGCTGGATGTGAACG	AGCGAAGCGCGCCGCCACGGTTATCAGGAAGGTATCGCGCTGGATGTGAACGGTTATATCTCTGAAG	50	2 quartet	3953058	0.404672	0.020482	CDS	3952484	3953413	+	b3770	*ilvE*

	GTTATATC	GCGCAGGCGAAAACCTGTTTGAA											
3262	CCAGTCTGGTGGTCGGTCTGGCGATGATCTACCTGATCGGTAAACCAGTT	CCGCTAATTTCCAGTCTGGTGGTCGGTCTGGCGATGATCTACCTGATCGGTAAACCAGTTGCTGGCATTCTCGAAGGGCTGACTCACTGG	50	2 quartet	2260295	0.78	0.021233	CDS	2259719	2261410	-	b2167	*fruA*
58	TGCGAGTTGTCATACTGGGAAGTGGTGTGGTAGGCGTTGCCAGCGCCTGG	TGCGAGTTGTCATACTGGGAAGTGGTGTGGTAGGCGTTGCCAGCGCCTGGTACTTAAATCAGGCAGGACATGAGGTCACCGTCATTGA	50	2 quartet	1237628	0.47202	0.021381	CDS	1237571	1238869	+	b1189	*dadA*
466	GCTGCTGACCATGACCGAAGAAGCCGGTATGGACGGTGCGTTCGGCTTAC	CGCTGGAAGTGCTGCTGACCATGACCGAAGAAGCCGGTATGGACGGTGCGTTCGGCTTACAGGGCAACTGGTTGCAGGCTGATATTCTGA	50	2 quartet	255251	0.971698	0.021551	CDS	254259	255716	-	b0237	*pepD*
1418	GTCGGCGGTGGCGGTCAGATGGGACGCCTGTTCGAGAAGATGCTGACCCT	GGTGGTTATCGTCGGCGGTGGCGGTCAGATGGGACGCCTGTTCGAGAAGATGCTGACCCTCTCGGGTTATCAGGTGCGGATTCTGGAGCA	50	2 quartet	2739711	0.924242	0.021749	CDS	2738948	2740069	-	b2600	*tyrA*
407	CACAAGAATTTGCCACTATTTATGTTTCCGGCGGCAAGCGCGGACTGGAT	GACGCCCCCGCACAAGAATTTGCCACTATTTATGTTTCCGGCGGCAAGCGCGGACTGGATATCGAACTGGCGGCAGGCGATCTGGCAAAG	50	2 quartet	506675	0.956897	0.021839	CDS	506603	507082	-	b0481	*ybaK*

1157	GCTATATTTGGTGGTTCGGCGGAGTACGTAGCGTTGTCGCTGAAATCAAT	GGTCGCTAATGCTATATTTGGTGGTTCGGCGGAGTACGTAGCGTTGTCGCTGAAATCAATAGGAATGGAAACAGCCTTCTTCTGGTATGT	50	2 quartet	2724551	0.46329	0.021846	CDS	2724448	2725746	-	b2587	*kgtP*
332	AGCGGCGACCTGGGCCAGGTTCTGGTCGATGCAATCAAGAAATACGATAT	CCTGAGCGGCAGCGGCGACCTGGGCCAGGTTCTGGTCGATGCAATCAAGAAATACGATATGGATTTGGTGGTTTGTGGTCACCACCAGGA	50	2 quartet	3640420	0.46717	0.022848	CDS	3640111	3640545	+	b3495	*uspA*
1662	ACGTGATTATCGCGCTGGGTGGTGGTTCCCCGATGGACGCCGCGAAGATC	TTCAAACCAGACGTGATTATCGCGCTGGGTGGTGGTTCCCCGATGGACGCCGCGAAGATCATGTGGGTTATGTACGAACATCCGGAAACT	50	2 quartet	1296460	0.383354	0.023669	CDS	1295446	1298121	-	b1241	*adhE*
2631	GGTGCGCGATTTGCTGGCGTTACGGGGCCGTATGGAAGCGGCCCAGATAA	CACTTATTCAGGTGCGCGATTTGCTGGCGTTACGGGGCCGTATGGAAGCGGCCCAGATAAGCCAGACATTGAACACTCCACAGCCAATGA	50	2 quartet	3542793	0.342708	0.023803	CDS	3542728	3542964	+	b3410	*feoC*
158	AAAACCGGGGACATTCTGGCTGAGGCGGCGCTTGGCTTACAGCGGGCGGG	GGAATGGGATAAAACCGGGGACATTCTGGCTGAGGCGGCGCTTGGCTTACAGCGGGCGGGCGCAGAAGGTATTGTGCTATGTACCAATAC	50	2 quartet	2980412	0.375	0.023839	CDS	2979943	2980635	-	b2840	*ygeA*
1838	ATCGGTGGCGTTTCTGGTGGGCACCACC	TGTTGCTGGGATCGGTGGCGTTTCTGGTGGGCACCACCAGCGGCC	50	2 quartet	4444219	0.44	0.024477	CDS	4444112	4447891	+	b4221	*tamB*

	AGCGGCCTGCATCTGGTATTTA	TGCATCTGGTATTTAAAGCGGCGGATCGCTGGGTGCCAGGACTGG											
73	CTGACTCTTACGACCCGTGGTTTAACCTGGCGGTGGAAGAGTGTATTTTT	CTGCTCATCTCTGACTCTTACGACCCGTGGTTTAACCTGGCGGTGGAAGAGTGTATTTTTCGCCAAATGCCCGCCACGCAGCGCGTTCTG	50	2 quartet	4624043	0.277586	0.025082	CDS	4623101	4624117	-	b4386	*lplA*
66	GGTGAGCAGGCGGTACTGGTACACATCTATTTTACGCAAGACAAAGATAT	TTATGATGCTGGTGAGCAGGCGGTACTGGTACACATCTATTTTACGCAAGACAAAGATATGGAAGACCTCCAGGAGTTTGAATCTCTGGT	50	2 quartet	4400741	0.427174	0.025186	CDS	4400672	4401952	+	b4173	*hflX*
172	CGCCTCGTGGGCACGGTTAACGCCGGCGAGGAAGTGACCTTATTACAAAC	AGATCATTATCGCCTCGTGGGCACGGTTAACGCCGGCGAGGAAGTGACCTTATTACAAACTGACGCCAACACCAATTATGCCCAGGTGAA	50	2 quartet	3201387	0.515	0.025631	CDS	3201207	3201827	+	b3055	*ygiM*
2702	TCCGGTGGGTTTGTGTGGCATGTATGCGCGTCGTGGCGAAGTTCAGGCAG	TGGCACTGGCTCCGGTGGGTTTGTGTGGCATGTATGCGCGTCGTGGCGAAGTTCAGGCAGCCAAAGCGGCGGACGCGCATGGTATTCCGT	50	G ≥ 40 %	3780102	0.265094	0.026112	CDS	3779827	3781017	+	b3605	*lldD*
89	GGGAAAAAGTGGAAGCGGCGATGGCGGAGCTGAATTACATTCCCAACCGC	GCGAAAACGCGGGAAAAAGTGGAAGCGGCGATGGCGGAGCTGAATTACATTCCCAACCGCGTGGCACAACAACTGGCGGGCAAACAGTCG	50	2 quartet	367358	0.923077	0.026248	CDS	366428	367510	-	b0345	*lacI*

848	AAGTAGATGCTCGTGTGGCCCAGGCCCAGGATGAACGCGGTATTATCATC	GTGAAAGAAAAAGTAGATGCTCGTGTGGCCCAGGCCCAGGATGAACGCGGTATTATCATCGTCTTTACCGGCAATGGAAAAGGCAAAACC	50	2 quartet	1328262	0.614286	0.026248	CDS	1327767	1328357	-	b1270	*btuR*
966	AGGTGACTCGTCTGGTTCACGGTGAAGAAGGTTTACAGGCGGCAAAACGT	CTGGCGGAGCAGGTGACTCGTCTGGTTCACGGTGAAGAAGGTTTACAGGCGGCAAAACGTATTACCGAATGCCTGTTCAGCGGTTCTTTG	50	2 quartet	1716257	0.767788	0.026594	CDS	1715948	1717222	-	b1637	*tyrS*
442	TCGGGCGCTGGCGCAGCTGCTGTGCCGTATTACGGGGGCGGAAGATGCCT	GACATCGCGATCGGGCGCTGGCGCAGCTGCTGTGCCGTATTACGGGGGCGGAAGATGCCTGTATCGTCAATAACAATGCGGCGGCGGTGT	50	G ≥ 40 %	3760808	0.585545	0.026674	CDS	3759858	3761249	-	b3591	*selA*
820	AATTAAGGGCGATCCTCGCTGGATGCAGGAACGCTCCTGGTTTGGCTATA	CAGAAAGTAAAATTAAGGGCGATCCTCGCTGGATGCAGGAACGCTCCTGGTTTGGCTATACGGAAGGGTTCCGGGAGCTGGTGCTGAAGA	50	2 quartet	2319039	0.914286	0.026901	CDS	2317027	2319876	-	b2218	*rcsC*
1468	TGGAGGATGCAGGCGTACGCTGGCTGGAACCGGCGTGGAAGAGCATTATC	TCAACCAAGCTGGAGGATGCAGGCGTACGCTGGCTGGAACCGGCGTGGAAGAGCATTATCTCCAACAAGGCACTTCTACCGCTACTGTGG	50	2 quartet	3181211	0.385762	0.027217	CDS	3180421	3181581	+	b3038	*ygiC*
1257	TGGGCCAGTGGGTA	CAGCCGCGCGTGGGCCAGTGGG	50	2 quartet	2852042	0.954545	0.027317	CDS	2851864	2852136	+	b2728	*hypC*

	CTGGTACACGTTGGCTTTGCCATGAGCGTAATTAAT	TACTGGTACACGTTGGCTTTGCCATGAGCGTAATTAATGAAGCCGAAGCACGCGACACTCTCGACGCC												
2974	GATGGCGGCGGGTTTGTCGCTGGAAGAGGATAAATTCAAACTCTTTCAAC	GCGGTCATGCGATGGCGGCGGGTTTGTCGCTGGAAGAGGATAAATTCAAACTCTTTCAACAACGGTTTGGCGAACTGGTTACTGAGTGGC	50	2 quartet	3036768	0.459211	0.027398	CDS	3036373	3038106	-	b2892	*recJ*
494	TTGAAAAACCGGCGAAAGTTGCGGCATTGATGGCGCAGTGGCTGGTTAAT	TGCGATATGGTTGAAAAACCGGCGAAAGTTGCGGCATTGATGGCGCAGTGGCTGGTTAATGGCTGGTGCCGTGAAACCATTTTCAACCTC	50	2 quartet	2940359	0.405405	0.027605	CDS	2940143	2941243	-	b2806	*rlmM*
1914	GGTCAGCTCGGTGGTACGCCGCCGGTGAAAGGCCAACAGCTTAACGCCTC	GGTTGCGGCGGGTCAGCTCGGTGGTACGCCGCCGGTGAAAGGCCAACAGCTTAACGCCTCTATTATTGCTCAGACGCGTCTGACCTCTAC	50	2 quartet	483706	0.807773	0.027803	CDS	481254	484403	-	b0462	*acrB*
619	GTTGGCGATGAAGTGGTCGCTGCGATTGAACGGCGGGCGAAAGAGGGCGA	CTGTCTGGTTGTTGGCGATGAAGTGGTCGCTGCGATTGAACGGCGGGCGAAAGAGGGCGATTTTCGTTCCAATTTGCATCGTGGCGGCGC	50	G ≥ 40 %	892591	0.68	0.027876	CDS	891967	892869	+	b0852	*rimK*
3215	TTAAACGCCGTATCCAGCCTGGTGACAAGATGGCAGGTCGTCACGGTAAC	TATCTGGCGGTTAAACGCCGTATCCAGCCTGGTGACAAGATGGCAGGTCGTCACGGTAACAAGGGTGTAATTTCTAAGATCAACCCGATC	50	2 quartet	4184459	0.546159	0.028959	CDS	4181245	4185273	+	b3987	*rpoB*

652	CTATTTACCCAGAA TCTGGCGCGGATGG CGGAGCAGGCGGG GGTTAAATT	AGACTGTCAGCTATTTACCCAG AATCTGGCGCGGATGGCGGAGC AGGCGGGGGTTAAATTCCGCTT TAATACGCCCGTTGACCAACTG CT	50	2 quartet	1238222	0.878453	0.029407	CDS	1237571	1238869	+	b1189	*dadA*
225	AGGCACCGGATCTG GCGGTGGCAGTTCA TCGCAAGGCCCGCG CCCGCAGC	GCGGCGGTAAAGGCACCGGATC TGGCGGTGGCAGTTCATCGCAA GGCCCGCGCCCGCAGCTTGGCG GTCGTGTCGTTACCATCGCAGCG G	50	2 quartet	4402262	0.256415	0.029678	CDS	4402038	4403297	+	b4174	*hflK*
1622	GTTCTACCGGTTTC ATTCCGGCGGGAAT GGACGGCAACGCTG AAGTTATC	GCACACGAAGGTTCTACCGGTT TCATTCCGGCGGGAATGGACGG CAACGCTGAAGTTATCGGCGCG TACGCATGGGCGCACGAAATGT CA	50	2 quartet	4619850	0.288315	0.030066	CDS	4619603	4620826	+	b4383	*deoB*
3591	GTTGCCCGCTGGCT GGATAACGGCGCGG TGGAGTACCTCGGG CGCAGTGA	TACCGGAGACGTTGCCCGCTGG CTGGATAACGGCGCGGTGGAGT ACCTCGGGCGCAGTGATGATCA GCTAAAAATTCGCGGGCAGCGT AT	50	2 quartet	616725	0.758621	0.03237	CDS	614157	618038	+	b0586	*entF*
490	GGTGAAACTGGGCG GTCGTCCGGAATAC CGTCAGGGCGTGGT GACCGATA	CGCGTCAGCTGGTGAAACTGGG CGGTCGTCCGGAATACCGTCAG GGCGTGGTGACCGATAATGGCA ACGTGATCCTCGACGTCCACGG CA	50	2 quartet	3058836	0.392055	0.03271	CDS	3058666	3059325	-	b2914	*rpiA*
920	GTCTGGTAGCACCT	CGTGTATTTGGTCTGGTAGCACC	50	2 quartet	3494930	0.310562	0.032939	CDS	3494011	3496554	+	b3365	*nirB*

	GGCTACAAAATGGCGCAGGTCGCCGTTGACCATATT	TGGCTACAAAATGGCGCAGGTCGCCGTTGACCATATTCTCGGTAGCGAAAACGCCTTTGAAGGTGCT												
422	AAGCGATCGGCACCACCGGTCGTGGTATCGGGCCTGCTTATGAAGATAAA	CGTGGCGCGAAAGCGATCGGCACCACCGGTCGTGGTATCGGGCCTGCTTATGAAGATAAAGTAGCACGTCGCGGTCTGCGTGTTGGCGAC	50	2 quartet	4405108	0.38373	0.033327	CDS	4404687	4405985	+	b4177	*purA*
769	TTATGGCGGCGGCGTGACAAAGCGCTGCGTACTGAAGCGCTGGCGGGAAC	ACCGGAACGGTTATGGCGGCGGCGTGACAAAGCGCTGCGTACTGAAGCGCTGGCGGGAACAGTAGCAAATAATCCTGATGATAAGCAGGC	50	G ≥ 40 %	2379871	0.6	0.034056	CDS	2379348	2380643	-	b2265	*menF*
2935	TTCTCCGGTGCTGTATAATGGCAACCTGGTGGTCGGTGACAGTGAAGGTT	GCCTGCTGACTTCTCCGGTGCTGTATAATGGCAACCTGGTGGTCGGTGACAGTGAAGGTTATCTGCACTGGATTAACGTCGAAGATGGTC	50	2 quartet	2637620	0.402326	0.03535	CDS	2637474	2638652	-	b2512	*bamB*
538	GCGGGTGCCGAGGCGTTGGACGTCGGCGTCGAACAACGTCGGCAATTAGA	TGTCTTCAGTGCGGGTGCCGAGGCGTTGGACGTCGGCGTCGAACAACGTCGGCAATTAGAAGAGCGACTGGTACTGCAACTGCGAATGAT	50	2 quartet	4161661	0.697646	0.035707	CDS	4161124	4161771	+	b3963	*fabR*
1449	GGTTATCAGCAGGCGGTTACGGTTAAACTGCTGAACCTGGAA	TAAGCCGCAGGGTTATCAGCAGGCGGTTACGGTTAAACTGCTGAACCTGGAACAGGCGGGCAAACC	50	2 quartet	2598312	0.305065	0.035748	CDS	2597831	2598865	-	b2477	*bamC*

	CAGGCGGG	GGTTGCAGACGCGGCTTCCATGCA												
1051	TCCGCTCTCCTGGGTGGTTGGCGATCAGGGCGTTTATCGCGCCAATATGC	AAGAACGTCGTCCGCTCTCCTGGGTGGTTGGCGATCAGGGCGTTTATCGCGCCAATATGCAATCAGAGCGCGAACGCAAGCGCGGTGAAC	50	2 quartet	2404022	0.443509	0.035899	CDS	2403951	2404613	-	b2287	*nuoB*
902	GACATCTCCGAGCGCGGCATGGTGCTCACCGGTGGTGGCGCACTGCTGCG	ACTGGCTTCCGACATCTCCGAGCGCGGCATGGTGCTCACCGGTGGTGGCGCACTGCTGCGTAACCTTGACCGTTTGTTAATGGAAGAAAC	50	2 quartet	3400186	0.287994	0.036446	CDS	3400044	3401087	-	b3251	*mreB*
862	GGTGAGCGGTGAGTATGCGATGATTAAGTTCGCCGCGCTGGCGGGTGCTA	GCGCGTATCAGGTGAGCGGTGAGTATGCGATGATTAAGTTCGCCGCGCTGGCGGGTGCTATAGATGAAGAGAAAGTCGTGCTCGAAAGCT	50	G ≥ 40 %	388866	0.522556	0.036805	CDS	388753	389727	-	b0369	*hemB*
2333	ACCGGCTTTGCCTGTGGCGGCTGGGCGCTGGCGTGGGCGGTATACGTCTT	GCTGATTGGCACCGGCTTTGCCTGTGGCGGCTGGGCGCTGGCGTGGGCGGTATACGTCTTTAACCGTGGGCAATACCATCCGCTGGTGCG	50	2 quartet	3143929	0.777778	0.037185	CDS	3142986	3144164	-	b2995	*hybB*
4393	CTGGTGTGCAGCAGGATAACTGGTTAGGTACAGGTTATGCTGTTGGTATC	AGCTTCCAGGCTGGTGTGCAGCAGGATAACTGGTTAGGTACAGGTTATGCTGTTGGTATCAACGGGACCAAAAACGATTACCAGACCTAT	50	2 quartet	199300	0.690179	0.037244	CDS	197928	200360	+	b0177	*bamA*
243	GGTATGGCCCAGAC	CGACGTTCTGGGTATGGCCCAG	50	2 quartet	3307670	0.612245	0.037803	CDS	3305971	3307860	-	b3162	*deaD*

	GGGGAGCGGAAAAACTGCAGCATTCTCTTTACCTCT	ACGGGGAGCGGAAAAACTGCAGCATTCTCTTTACCTCTGTTGCAGAATCTTGATCCTGAGCTGAAAGC												
140	GCGCATGGGGCCGGGGCAGGCGTTGCAATTGTTTGACGGTAGCAACCAGG	GGCGCGTACTGCGCATGGGGCCGGGGCAGGCGTTGCAATTGTTTGACGGTAGCAACCAGGTCTTTGACGCCGAAATTACCAGCGCCAGCA	50	G ≥ 40 %	3091277	0.792308	0.037837	CDS	3091134	3091865	+	b2946	*rsmE*
143	GATGGCGGGCTGCTGGCAAAAGTGCGCGACGGGGACATCATTCGTGTGAA	AGAAGCCTACGATGGCGGGCTGCTGGCAAAAGTGCGCGACGGGGACATCATTCGTGTGAATGGACAGACAGGCGAACTGACGCTGCTGGT	50	G ≥ 40 %	1932965	0.295821	0.037956	CDS	1932793	1934604	-	b1851	*edd*
848	GATAACTCCTATAAAGTGTCCGGCGGTCTGCACGGCGTTGGTGTTTCGGT	TAAATTTGACGATAACTCCTATAAAGTGTCCGGCGGTCTGCACGGCGTTGGTGTTTCGGTAGTAAACGCCCTGTCGCAAAAACTGGAGCT	50	2 quartet	3879755	0.286111	0.038294	CDS	3877705	3880119	-	b3699	*gyrB*
1271	GAGGTTAAATGCGTCGGCGTGACTGCGGGCGCATCGGCTCCGGATATTCT	GTGGGTGAAAGAGGTTAAATGCGTCGGCGTGACTGCGGGCGCATCGGCTCCGGATATTCTGGTGCAGAATGTGGTGGCACGTTTGCAGCA	50	2 quartet	27096	0.564286	0.038361	CDS	26277	27227	+	b0029	*ispH*
2143	CTGGTTGGTACTGGTATGGAACGTGCTGTTGCCGTTGACTCC	TGATAAGCCGCTGGTTGGTACTGGTATGGAACGTGCTGTTGCCGTTGACTCCGGTGTAACTGCGGTA	50	2 quartet	4183387	0.330538	0.03866	CDS	4181245	4185273	+	b3987	*rpoB*

	GGTGTAAC	GCTAAACGTGGTGGTGTCGTTCA												
1818	ACTTCTATGGCAAGGTGCCGGTTTACCGGTCGCGCCGTGGGTAGCGTTAA	TACGCAGCAAACTTCTATGGCAAGGTGCCGGTTTACCGGTCGCGCCGTGGGTAGCGTTAACCCGCGCAGAGTTTGAAAAAGGCCTGAGCG	50	2 quartet	102583	0.25337	0.038795	CDS	102233	103153	+	b0092	*ddlB*
1729	AAATCTCAGCCGGTACTGGTTCAGCCTGGTCAGACTGGCGCGATGAACAG	TATCGGCTATAAATCTCAGCCGGTACTGGTTCAGCCTGGTCAGACTGGCGCGATGAACAGCACCCTGTGGGTTGGCCCGGAAATCCAGGA	50	2 quartet	3885988	0.301601	0.03944	CDS	3885076	3886722	+	b3705	*yidC*
1256	GCGTTCAGCCTGCACACTGGAGTGGCGATGTGGATACGCTGGCAGATATG	GCAGAAGCCTGCGTTCAGCCTGCACACTGGAGTGGCGATGTGGATACGCTGGCAGATATGGTGGTGAAAACCGCTCAGCCTGGCGACCAT	50	2 quartet	4457040	0.801627	0.039637	CDS	4455785	4457158	+	b4233	*mpl*
5306	AGGATACGCGTCTGGCGTTTGGTGAACTGGCTGCATGGGTTCGCCAGCAA	TTAATCGTCAAGGATACGCGTCTGGCGTTTGGTGAACTGGCTGCATGGGTTCGCCAGCAAGTTCCGGCGCGCGTGGTTGCTCTGACGGGG	50	2 quartet	94939	0.638894	0.039827	CDS	94650	96008	+	b0086	*murF*
76	GATGAACATTGCGTCTTTGGTTGTATCGGTGGTGGTTCTTCTTATCGGGC	ATTCAGGCGTGATGAACATTGCGTCTTTGGTTGTATCGGTGGTGGTTCTTCTTATCGGGCTCATCTTGTGGTTTTTTATCAATCGTGCCA	50	2 quartet	1908473	0.35	0.040357	CDS	1908261	1908548	-	b1825	*yebO*

658	CCATCGAACTGCCGGAAGGCGTAGAGATGGTAATGCCGGGCGACAACATC	GTGACTGGTACCATCGAACTGCCGGAAGGCGTAGAGATGGTAATGCCGGGCGACAACATCAAAATGGTTGTTACCCTGATCCACCCGATC	50	2 quartet	4177013	0.891775	0.040401	CDS	4175944	4177128	+	b3980	*tufB*
339	AAACGAGGCCTGGACGTGGGAACATCAGGCGCTGGTGCGTGCGCGTGTAG	ATTATCAGAAAAACGAGGCCTGGACGTGGGAACATCAGGCGCTGGTGCGTGCGCGTGTAGTGTACGGCGATCCGCAGCTCACCGCGCACT	50	2 quartet	3197265	0.733333	0.040429	CDS	3196801	3199641	-	b3053	*glnE*
816	ACCTGGCGGTAGCTGCGGGTCATCCGCTGGCGCAGAAAGCGGCGGAAAAT	GGTTGTACCTACCTGGCGGTAGCTGCGGGTCATCCGCTGGCGCAGAAAGCGGCGGAAAATAATCCTGAACTGGCGGCCTTTATTGACGAA	50	2 quartet	673962	0.89899	0.040968	CDS	672201	674783	-	b0642	*leuS*
1034	ATATTATCGACATGGATAAGATGGAACGGCGCAAGGTCCAGCTTGATAAT	ATCTTTAACGATATTATCGACATGGATAAGATGGAACGGCGCAAGGTCCAGCTTGATAATCAACCGGTTGATTTCACCAGCTTCCTTGCC	50	2 quartet	3351967	0.590164	0.041228	CDS	3350689	3353025	-	b3210	*arcB*
148	TGGACGTATACGGTGTGACGCCTGCCCGGTGCCGGAAGGTTAATTGATGG	AACACGAAAGTGGACGTATACGGTGTGACGCCTGCCCGGTGCCGGAAGGTTAATTGATGGGGTTAGCCGCAAGGCGAAGCTCTTGATCGA	50	2 quartet	4039377	0.604282	0.041841	rRNA_CDS	4037519	4040423	+	b3854	*rrlA*
986	CAAAGTCTCTCAGGCCTTCTGGCACGAA	GTCCACGCGACAAAGTCTCTCAGGCCTTCTGGCACGAATGGCGT	50	2 quartet	4381403	0.457519	0.042188	CDS	4380510	4382318	-	b4154	*frdA*

	TGGCGTAAAGGCAACACCATCT	AAAGGCAACACCATCTCCACGCCGCGTGGCGATGTGGTTTATCTCG											
613	AATCATCGACACACCGCCGGTGCTGGTACGCGACGGTGGTGTTATCGCAT	TGGAGCGAGCAATCATCGACACACCGCCGGTGCTGGTACGCGACGGTGGTGTTATCGCATCGGGCTATAACGAAGAGCTGGATGAGTGGC	50	2 quartet	2858370	0.908046	0.043134	CDS	2857093	2859654	+	b2733	*mutS*
1463	CCTGGCGCAGGCGATTGGCCGTAACGGTCAGAACGTGCGTCTGGCTTCGC	AAGCCGGTAACCTGGCGCAGGCGATTGGCCGTAACGGTCAGAACGTGCGTCTGGCTTCGCAGCTGAGCGGTTGGGAACTCAACGTGATGA	50	2 quartet	3316539	0.445102	0.043495	CDS	3316039	3317526	-	b3169	*nusA*
108	ACCACTTGCTGAGTCGATTAATCAGGAACCTGGTTTTCTGTGGAAGGTAT	AGCAGCTTAAACCACTTGCTGAGTCGATTAATCAGGAACCTGGTTTTCTGTGGAAGGTATGGACAGAAAGTGAAAAGAACCACGAAGCCG	50	2 quartet	1746819	0.6125	0.043629	CDS	1746700	1747005	+	b1667	*ydhR*
2432	GTTATCTCTATCTCAGCCCGGAACGGCTGGCGAATGAGGGGATTTTCACC	AAAATCCTCTGTTATCTCTATCTCAGCCCGGAACGGCTGGCGAATGAGGGGATTTTCACCCAGCAGGAACTGTACGACGAACTGCTCACC	50	2 quartet	975968	0.433704	0.043745	CDS	975622	976326	+	b0923	*mukE*
597	AAAGATCCGATTCTTGGTCTGGTGGCAGGTATTCAGCTTTCC	GTTGGTATTTAAAGATCCGATTCTTGGTCTGGTGGCAGGTATTCAGCTTTCCGCGAACGATATGCTGA	50	2 quartet	604065	0.277778	0.043751	CDS	603416	604663	-	b0577	*ybdG*

	GCGAACGA	AACTGGGCGACTGGCTGGAGAT											
620	AACCCGCTGATCTACTTTGCGGTTGCAACGGTTCTGGAACTGGTGTTTGG	GAGCAACGGCAACCCGCTGATCTACTTTGCGGTTGCAACGGTTCTGGAACTGGTGTTTGGTATTCTGGCGAGCATTATCACCATGTGGTT	50	2 quartet	1911957	0.759524	0.044159	CDS	1911695	1912576	-	b1829	*htpX*
502	GTGACGGGTGCCAGTGGTGGCGTCGGCAGTACCGCCGTGGCGCTGCTGCA	GGAGATTGTCGTGACGGGTGCCAGTGGTGGCGTCGGCAGTACCGCCGTGGCGCTGCTGCATAAGTTGGGTTATCAGGTCGTTGCCGTTTC	50	2 quartet	3403985	0.803922	0.044717	CDS	3403484	3404458	+	b3253	*acuI*
96	TGATCTGGTGGGCGAAGATGTGCTGGTTTCTGGTGCAGGCCCGATTGGTA	CGCTGTCGTTTGATCTGGTGGGCGAAGATGTGCTGGTTTCTGGTGCAGGCCCGATTGGTATTATGGCAGCGGCGGTGGCGAAACACGTTG	50	2 quartet	3790817	0.70004	0.045009	CDS	3790320	3791345	-	b3616	*tdh*
261	CGTTGCGGGTGCGCAAAATTGAGGCACTGGCGGATGGCATTATGGATGCC	CCGCTGGAGTCGTTGCGGGTGCGCAAAATTGAGGCACTGGCGGATGGCATTATGGATGCCGGGCTGGTATCGGTGCGTGAACAGGCGCGT	50	2 quartet	3761647	0.392941	0.045431	CDS	3761347	3761955	-	b3592	*yibF*
300	ACTACCTGGTTGATGTGATCCTGGATGAAGCGGCTAACAAAGGTACCGGT	GAAGACGGTAACTACCTGGTTGATGTGATCCTGGATGAAGCGGCTAACAAAGGTACCGGTAAATGGACCAGCCAGAGCGCGCTGGATCTC	50	2 quartet	2100480	0.329936	0.045664	CDS	2099862	2101268	-	b2029	*gnd*
741	GTCATCTTAGCCCGGATGGCAGCGGCCC	ATTTACCATCGTCATCTTAGCCCGGATGGCAGCGGCCCGGCGTTA	50	2 quartet	2530493	0.27954	0.045709	CDS	2530247	2531233	-	b2412	*zipA*

	GGCGTTATTCAGCC TGGCGAAT	TTCAGCCTGGCGAATATGGTGA AACCGGGAACCTTTGATCCTGA A												
126	CAAACGGGACCGAG GCAACGACGGGTAC TACTGGCACCACAA CGACCACT	CCTGAAGCAACAAACGGGACCG AGGCAACGACGGGTACTACTGG CACCACAACGACCACTACCGGC GCAACCACGACTGCTACTACCA CT	50	2 quartet	1049619	0.273721	0.04583	CDS	1049439	1049744	-	b0987	*gfcA*
150	CGGCGAATCTGGCG CAGGTCTGGTAAAA GTGACCATCAACGG TGCACACA	TGGAAGTCACCGGCGAATCTGG CGCAGGTCTGGTAAAAGTGACC ATCAACGGTGCACACAACTGCC GTCGCGTAGAGATCGACCCGAG CC	50	2 quartet	494225	0.325221	0.045908	CDS	494076	494405	+	b0471	*ybaB*
1493	CTGTTGCTGCTGAT GGCTTTCGTCATCT GGTTTGGTAAGGAT CTGATGGT	GGCGCTGTTCCTGTTGCTGCTGA TGGCTTTCGTCATCTGGTTTGGT AAGGATCTGATGGTTAAAGTGA TGAGCTACCTGGTATGGCCGTT	50	2 quartet	3264536	0.295522	0.046735	CDS	3263686	3265017	-	b3116	*tdcC*
1421	CCACCGGCACCGGT ATTGGTCTGGCCGT TTCTCGTCGTCTGGC GAAAAAT	GGTAAACCTGCCACCGGCACCG GTATTGGTCTGGCCGTTTCTCGT CGTCTGGCGAAAAATATGGGCG GCGATATTACGGTTACCAGCGA A	50	2 quartet	3351580	0.560714	0.047373	CDS	3350689	3353025	-	b3210	*arcB*
1681	CCCGTTTCCACGCA CCTGGCGGTTTTGG CGTACGTTGGGAGT CTCATATC	GGCAAAATCACCCGTTTCCACG CACCTGGCGGTTTTGGCGTACGT TGGGAGTCTCATATCTACGCGG GCTACACCGTACCGCCGTACTAT	50	2 quartet	3407028	0.411714	0.047546	CDS	3405917	3407266	+	b3256	*accC*

186	TAGGCGCAGCGCACGTTCACCTGGTGTACGGTCACGGCGGCGATCTGCTA	GCGAATGAATTAGGCGCAGCGCACGTTCACCTGGTGTACGGTCACGGCGGCGATCTGCTAAAACAGGCGCTGAAAGACGACAACCTTAAC	50	2 quartet	3915015	0.281226	0.048905	CDS	3913830	3915200	-	b3730	*glmU*
282	AGGCACTGGCGGATGGCATTATGGATGCCGGGCTGGTATCGGTGCGTGAA	CGCAAAATTGAGGCACTGGCGGATGGCATTATGGATGCCGGGCTGGTATCGGTGCGTGAACAGGCGCGTCCAGCGGCGCAGCAGTCTGAA	50	2 quartet	3761626	0.687432	0.048952	CDS	3761347	3761955	-	b3592	*yibF*
141	GCTGGTGGATGGAGCTGGAAGCGCAGGAATCCCGTTTTACCTACAGTTAC	GAATTCTGGGGCTGGTGGATGGAGCTGGAAGCGCAGGAATCCCGTTTTACCTACAGTTACCAGTTTGGTCTGTTCGATAAAGCAGGCGAC	50	2 quartet	258816	0.346364	0.049033	Pseudo gene	257829	259006	+	b0240	*crl*
1877	CTGCGCTGGTGGGCGGCATGCGTGTACTGGGTGCCAACTTCGATGGCAGC	CCGGAAATGACTGCGCTGGTGGGCGGCATGCGTGTACTGGGTGCCAACTTCGATGGCAGCAAAAACGGCGTCTTCACTGACCGCGTTGGC	50	G ≥ 40 %	4135711	0.715112	0.049257	CDS	4133835	4136015	+	b3942	*katG*
752	TCGCTCAACGGATAAAAGGTACTCCGGGGATAACAGGCTGATACCGCCCA	GGAAGGGCCATCGCTCAACGGATAAAAGGTACTCCGGGGATAACAGGCTGATACCGCCCAAGAGTTCATATCGACGGCGGTGTTTGGCAC	50	2 quartet	3946170	0.619935	0.049435	rRNA_ gene	3943704	3946607	+	b3758	*rrlC*

Table S1B. Whole-genome location analysis of rG4 sites from rG4-seq in in *P. aeruginosa*.

Position	Sequence_50bp	Sequence_90bp	Length	Class	POS	RTS	P value	Region	Start	End	Strand	Locus_tag	Name
1496	GTCGAGCAACTGGTGGA TCGCGGGCTGGTCGCCT CGCCGGCGGATCTCTA	CGACAAGATCGTCGAGCA ACTGGTGGATCGCGGGCT GGTCGCCTCGCCGGCGGA TCTCTATACCCTGACCTAT GAACAGGTGTTCGAGCT	50	2 quartet	1667520	0.287181	0.001008	CDS	1666025	1668409	+	PA1529	*lig*
311	CAGCGCAACCTGGTGGG CATGGTATTGCGCCGGA TCGAGACCAACATCCC	TGCCTTCTACCAGCGCAA CCTGGTGGGCATGGTATT GCGCCGGATCGAGACCAA CATCCCGACCCTGGAAGA GCTGAAGCTCCCGGAAAT	50	2 quartet	438092	0.349026	0.001112	CDS	437782	438930	+	PA0396	*pilU*
1624	GCGGACGGCCAGGCGC TGGCGCTGCACGATCTG CCGTTGCTCGGGTTGCC	CGGGCTGGCCGCGGACGG CCAGGCGCTGGCGCTGCA CGATCTGCCGTTGCTCGG GTTGCCCATGGAAAACGC CGCGCTGGCCCTGCAGGC	50	2 quartet	3491950	0.355098	0.001199	CDS	3491415	3492704	-	PA3111	*folC*
614	GGTCGCCTGGTGGATAG CGTGCAGGCCCTGCAGC AGGCCGGCGCGCGCTA	ACAGGCCGCCGGTCGCCT GGTGGATAGCGTGCAGGC CCTGCAGCAGGCCGGCGC GCGCTACATCGTGGTCTG GCTGTTGCCCGACCTGGG	50	G ≥ 40 %	5755625	0.401009	0.001869	CDS	5754298	5756238	-	PA5112	*estA*
3156	CCGAAGGCGTGTTCCGG CGCAAGGTCGAGGAGG CCGGGCTGGCTTCCCGC	TCGCCCACCGCCGAAGGC GTGTTCCGGCGCAAGGTC GAGGAGGCCGGGCTGGCT	50	2 quartet	3337594	0.484043	0.001878	CDS	3337228	3337692	-	PA2978	*ptpA*

		TCCCGCATCCATGTCGATT CCGCCGGTACCGCCGGC												
6442	TTCGACAAGGGCGTGAT GGAGGACGGCGAAGGC CGGGTGATCGACTTCAA	CTTCCAGGTCTTCGACAA GGGCGTGATGGAGGACGG CGAAGGCCGGGTGATCGA CTTCAAGAACACCCTGAT CCTGCTCACCACCAACGC	50	2 quartet	110454	0.378196	0.002128	CDS	108221	110929	+	PA0090	*clpV1*
360	GCGAGGACGGCACCCT GCAGGCGCTGCTGGATG CCGGTGGTTTCGCCTAT	GGCGGCACCGGCGAGGAC GGCACCCTGCAGGCGCTG CTGGATGCCGGTGGTTTC GCCTATACCGGCAGCGGC CACCTGGCCAGCGCCATG	50	2 quartet	4701587	0.440719	0.003888	CDS	4700906	4701946	-	PA4201	*ddlA*
1069	CTATCTCTCCACCTCCCT GAACCCCGGTGTCGCGA GGAGCTTCGGGCAGG	ACGACGACGGCTATCTCT CCACCTCCCTGAACCCCG GTGTCGCGAGGAGCTTCG GGCAGGGCACGATATCCA CCGTGTTCGGCAGGTCCG	50	2 quartet	4303434	0.295195	0.003967	CDS	4303141	4304502	-	PA3841	*exoS*
2244	GCGCCGCGCCCTGGAGG TGCGCCGGCTGGTCCTG GAGAACCGCACCCTGC	TCGACAGCGTGCGCCGCG CCCTGGAGGTGCGCCGGC TGGTCCTGGAGAACCGCA CCCTGCGCCTGGCCCTGG CCGAACGCCACGAGCTGC	50	2 quartet	5815976	0.303763	0.004114	CDS	5815562	5816950	+	PA5166	*dctD*
33	GGAGGAGGCGGGTTTC GGACCAGGGAGGTCGA A	GGAGGAGGCGGGTTTCGG ACCAGGGAGGTCGAACAT GCACACGGGAGAAACACT CGCCGCCGC	33	2 quartet	3958721	0.78945	0.004178	CDS	3958288	3958755	-	PA3536	*PA3536*

3565	GGCAACGACCTGGCCAT CGTCAGCGGCGGCGCG GTGACCTTCGAGGCGGT	GCTGGAATCGGGCAACGA CCTGGCCATCGTCAGCGG CGGCGCGGTGACCTTCGA GGCGGTGAAGGACCTGCA CCAGGAAAGCCACGAGAA	50	G ≥ 40 %	51387	0.357721	0.004381	CDS	42914	53521	+	PA0041	*PA0041*
2002	GAAGGCCATCGCCGAG GAAGAAGGCGCGGTGG TGGTGCCGGTGTGCAAC A	TGGATGTGGTGAAGGCCA TCGCCGAGGAAGAAGGCG CGGTGGTGGTGCCGGTGT GCAACAAGATCGAAGCCG AGATCGCCGAGCTGGAAG	50	2 quartet	5241467	0.288223	0.004486	CDS	5240747	5241847	+	PA4673	*PA4673*
220	GACCTGCCCTGCGGCGC CGGACGCTTCTGGCCGC TGCTGGCGGAAAAGGA	CCTGGTGCTCGACCTGCC CTGCGGCGCCGGACGCTT CTGGCCGCTGCTGGCGGA AAAGGACAACCGGGTGAT CATCGGCGCCGACAATTC	50	2 quartet	4910571	0.348214	0.004704	CDS	4910108	4910791	-	PA4379	*PA4379*
361	GAAGGTCGGGCGCATG GCCGGCCAGTTCGCCAA GCCGCGCTCCTCCGGCG	GCCCGGTAGTGAAGGTCG GGCGCATGGCCGGCCAGT TCGCCAAGCCGCGCTCCT CCGGCGACGAAACGCAGA ACGGCGTGACCCTGCCCG	50	2 quartet	3198002	0.302941	0.004753	CDS	3197642	3198988	+	PA2843	*PA2843*
1980	TTGGCGAAGGCCTTGGG CGTGGAGCTGGTGCGCT TCGACATGTCCGAGTA	GGCGCGGCAGTTGGCGAA GGCCTTGGGCGTGGAGCT GGTGCGCTTCGACATGTC CGAGTACATGGAGCGGCA TACCGTGTCGCGGCTGAT	50	2 quartet	2962994	0.330155	0.004787	CDS	2962303	2964579	-	PA2620	*clpA*
1126	GCGCGGCGTGCCGGTGG	TGTCGCACCAGCGCGGCG	50	2 quartet	5623669	0.288997	0.005079	CDS	5623040	5624797	-	PA5005	*PA5005*

	AGAAGATGGAGCACGT<br>CTACCTCGGCCCGGAGT	TGCCGGTGGAGAAGATGG<br>AGCACGTCTACCTCGGCC<br>CGGAGTATTCCAACGAGG<br>ACGTCATCGCCGCCTGCG												
1573	GACACCCTGGAGTCGGT<br>GCGGGCGGAACTGGCG<br>GCCGACGACCAGTTGTT	CTACACCATCGACACCCT<br>GGAGTCGGTGCGGGCGGA<br>ACTGGCGGCCGACGACCA<br>GTTGTTCATGCTGATCGGC<br>TGGGATGCCTTCTGCGG	50	2 quartet	4486540	0.410626	0.005338	CDS	4486203	4486847	-	PA4006	*nadD1*
244	GGAGAAGGCCAGGCGC<br>CAACTGGTCTCGCGCAT<br>GGGGGTCGACCCCGAG<br>C	TGTCACCGAAGGAGAAGG<br>CCAGGCGCCAACTGGTCT<br>CGCGCATGGGGGTCGACC<br>CCGAGCATGGCTGGAAGG<br>CCAAGTACGAGATCCTGC	50	2 quartet	3372463	0.513955	0.005363	CDS	3370100	3372706	-	PA3011	*topA*
208	CGCGCTGATGGTGCCGG<br>CGGCGCGCCAGGCCATC<br>GAGGCTGCCGGGCTGC	AGGCGGTCAGCGCGCTGA<br>TGGTGCCGGCGGCGCGCC<br>AGGCCATCGAGGCTGCCG<br>GGCTGCTGCCGGAGGACA<br>TCGACCTGTTGCTGGTGA	50	2 quartet	1082149	0.3	0.005738	CDS	1081942	1082955	+	PA0999	*pqsD*
2531	CCGTGCTACGGCACAAG<br>CCGCATGGCGTGGTGGC<br>GGTATTCGGTCCCTAC	GACGCTACCGCCGTGCTA<br>CGGCACAAGCCGCATGGC<br>GTGGTGGCGGTATTCGGT<br>CCCTACAATTTCCCCGGCC<br>ACCTGCCCAACGGGCAT	50	2 quartet	981859	0.328691	0.005975	CDS	981422	982888	+	PA0898	*aruD*
908	GGCAACCCGCTGGCGTC<br>GGCGGTGGCCGAGGCG	CACCTACGGCGGCAACCC<br>GCTGGCGTCGGCGGTGGC	50	2 quartet	978817	0.592104	0.006241	CDS	977910	979130	+	PA0895	*aruC*

	GCGCTGGACGTGATCAA	CGAGGCGGCGCTGGACGT<br>GATCAATACCCCGGAAGT<br>GCTGGATGGCGTGAAGGC											
3665	TGGTCCAGGACGGTACC<br>TTGCGCCAGGGCGACAT<br>GGTGCTGGTCGGCATC	GCCACCGTGCTGGTCCAG<br>GACGGTACCTTGCGCCAG<br>GGCGACATGGTGCTGGTC<br>GGCATCAACTACGGTCGT<br>GTCCGCGCCATGCTCGAC	50	2 quartet	5328294	0.312651	0.006892	CDS	5327427	5329949	-	PA4744	*infB*
839	CCAGGTGCTGGCCAAGG<br>TCGGCAGCACCGGACGC<br>TCCACCGGCTACCACG	TCAAGCGCGGCCAGGTGC<br>TGGCCAAGGTCGGCAGCA<br>CCGGACGCTCCACCGGCT<br>ACCACGTACATTTCGAAG<br>TGATGAAGGATGGCCGGG	50	2 quartet	4936827	0.78183	0.00723	CDS	4936750	4937673	-	PA4404	*PA4404*
2394	TCACCGGCCTCGAGGCC<br>CTGGTGAAAGGTAACTA<br>TATCGACGTGCGCTTC	CTGGCCGGCATCACCGGC<br>CTCGAGGCCCTGGTGAAA<br>GGTAACTATATCGACGTG<br>CGCTTCGCCAAGAGCGGC<br>GCGCCGAGTCGCGAGTTC	50	2 quartet	5261543	0.258621	0.007644	CDS	5260343	5262649	-	PA4689	*PA4689*
1063	GGCCGGCAACCCCACG<br>GTCGCTTCGGCCAACCC<br>GAGCACGCTACTGCATA	TCCCGCCGCTGGCCGGCA<br>ACCCCACGGTCGCTTCGG<br>CCAACCCGAGCACGCTAC<br>TGCATATCACCCTTACCG<br>GCTGGAAAACCGCGCAGA	50	2 quartet	5119600	0.454107	0.008698	CDS	5118538	5120565	+	PA4571	*PA4571*
2844	CCCGGCGACAAGTGGC<br>AGGGAGGGGCCGGCGG<br>TGAGTGACACCCAGCAG	GATCGTCGTTCCCGGCGA<br>CAAGTGGCAGGGAGGGGC<br>CGGCGGTGAGTGACACCC	50	bulges	3258342	0.79661	0.008917	CDS	3256681	3258360	-	PA2903	*cobJ*

	G	AGCAGGTTCCGGCCATCG<br>TCATCCTCGGCCAGGGCG											
1251	GCGGGACCAACGGCAC<br>CCTGGTGTTCCGCAGGT<br>TCGCCGACTGATGCTGG	TTCGGTTTCGGCGGGACC<br>AACGGCACCCTGGTGTTC<br>CGCAGGTTCGCCGACTGA<br>TGCTGGACTGGGTCGACG<br>GCCGGCCCGCCGCCGAGC	50	2 quartet	3323568	0.779228	0.00903	CDS	3322759	3323574	-	PA2964	*pabC*
565	CGGCTCCTGGGCCGGGG<br>CCTTCGGCCATACCCAG<br>TTCATGCCATCGACCT	CCGGCATCACCGGCTCCT<br>GGGCCGGGGCCTTCGGCC<br>ATACCCAGTTCATGCCAT<br>CGACCTACGCGCGGATCG<br>CCGTGGACTTCGACGGCG	50	2 quartet	1271536	0.299411	0.009105	CDS	1270972	1272168	+	PA1171	*sltB2*
1153	ACTGGCCGAGGTGGAG<br>GTGAAGAACCGCTTCCA<br>GCGCGGCGACAGCGTC<br>G	GCCGCGACGGACTGGCCG<br>AGGTGGAGGTGAAGAACC<br>GCTTCCAGCGCGGCGACA<br>GCGTCGAACTGATGACTC<br>CGCGAGGCAACCTGAGCC	50	2 quartet	6124838	0.294723	0.009257	CDS	6123686	6125080	+	PA5440	*PA5440*
2613	AATACGGCCAGTTCGGT<br>GGCCAGCCGGTCGGCGC<br>CATCATCGCCAACTAC	TACACCGCCGAATACGGC<br>CAGTTCGGTGGCCAGCCG<br>GTCGGCGCCATCATCGCC<br>AACTACTACATGTCGCCC<br>AGTTCGCCCGACGTCAAG	50	2 quartet	1806238	0.328829	0.009493	CDS	1805753	1807228	+	PA1658	*hsiC2*
1628	GTTCAACTGGTACGCCG<br>GGTTACCGGAGGTGGAC<br>GCCAAGCTGGTCTTCG	GCAAGCCGCTGTTCAACT<br>GGTACGCCGGGTTACCGG<br>AGGTGGACGCCAAGCTGG<br>TCTTCGAGCAGCAGGTGC	50	2 quartet	1143690	0.393352	0.009544	CDS	1142061	1144862	+	PA1054	*shaA*

		AACGTGTGGTGGCCCTGG											
994	GGGCAAGTTCAACGGC<br>GCCTCCGAGGCCGTGGC<br>GGCCGCCGCGGCGGTA<br>G	TGCACGGCAAGGGCAAGT<br>TCAACGGCGCCTCCGAGG<br>CCGTGGCGGCCGCCGCGG<br>CGGTAGCCAAGAACATCG<br>CGGCGAAGAGTCCCGCGG	50	2 quartet	1588016	0.262274	0.009968	CDS	1587023	1589284	+	PA1458	*PA1458*
198	GGTGGTGAGTCGGGACT<br>TCGACCGGGACAAGCGT<br>CTACTGACAGCCCGGC	ATCCGGTACGGGTGGTGA<br>GTCGGGACTTCGACCGGG<br>ACAAGCGTCTACTGACAG<br>CCCGGCAATTCAGCGCAG<br>TCTTCGACTCTCCGACCG	50	2 quartet	6264164	0.340583	0.010552	CDS	6263805	6264212	-	PA5569	*rnpA*
68	TACCCAGATCTACGCCC<br>TGGTTCCGCCGGTATCC<br>GGTACCGGTGCCGCTA	TGACTCAAGGTACCCAGA<br>TCTACGCCCTGGTTCCGCC<br>GGTATCCGGTACCGGTGC<br>CGCTACCGTCCTGGAGAT<br>CGAAGGCGTGACCTCGT	50	2 quartet	689606	0.365471	0.011017	CDS	689537	690031	+	PA0633	*PA0633*
775	GGCCAGTGGGCCTTGCC<br>CGGCGTGCTGGTCAACG<br>GCCGCAGCGCCGACCA	GCCTTTCGCCGGCCAGTG<br>GGCCTTGCCCGGCGTGCT<br>GGTCAACGGCCGCAGCGC<br>CGACCACAGCCTCGACGA<br>CGCGGCGGTGCGCGCCCT	50	2 quartet	5516937	0.480582	0.011881	CDS	5516399	5517094	-	PA4916	*nrtR*
2163	CGGCTTCGCCCATCCTG<br>TACATCTCTACTGGGGC<br>GTTCGGCGTCCGGAGG	GCCGCGCCGCCGGCTTCG<br>CCCATCCTGTACATCTCTA<br>CTGGGGCGTTCGGCGTCC<br>GGAGGATTTCTACCGACT<br>GCCGCACTGGACGGAGT	50	2 quartet	5894562	0.35636	0.012237	CDS	5894293	5895261	-	PA5236	*PA5236*

1660	GCCGCGGCCTTCGGCCT GGCGGCCCTGGACAAG CCGGCGCAGATCGTCAT	CGACAAGCTGGCCGCGGC CTTCGGCCTGGCGGCCCT GGACAAGCCGGCGCAGAT CGTCATCTGGACCACCAC GCCCTGGACCATCCCGGC	50	2 quartet	5109076	0.373086	0.012718	CDS	5106951	5109782	-	PA4560	*ileS*
584	GGGACCGGGGCGCTGG GCGGTCAAGCCGGCGAT CCGCAGCCGCGTCGAGT	TCGACGCCAAGGGACCGG GGCGCTGGGCGGTCAAGC CGGCGATCCGCAGCCGCG TCGAGTTCCGTGCCCTGA ACCTGCTGGACAGCTACG	50	2 quartet	3760235	0.264515	0.013131	CDS	3759996	3760820	-	PA3348	*PA3348*
4130	CCCTCGAAGCCGAAGGC TTGCAGGCCGCGGTCGG CAGCCGCTGCAACGTG	GTCGGCCTGACCCTCGAA GCCGAAGGCTTGCAGGCC GCGGTCGGCAGCCGCTGC AACGTGATCAACGAAAGC GGCTACCACCCGGTGCAG	50	2 quartet	1196182	0.496769	0.0134	CDS	1196021	1197376	+	PA1104	*fliI*
338	CGCTGGTGGAGAGCGG CGAGAAGCTGCGAGTG GTCGGCAACCTGCCCTA C	GATTTCGCCTCGCTGGTG GAGAGCGGCGAGAAGCTG CGAGTGGTCGGCAACCTG CCCTACAACATCTCCACG CCACTGATCTTCCATCTG	50	G ≥ 40 %	651043	0.566231	0.013826	CDS	650575	651381	-	PA0592	*ksgA*
1021	AGCCTGCCATCGGTGCC CAGTGGCTGTTCCTCGG CAAGGGCCTCGGCGCC	TGGTGGAACCAGCCTGCC ATCGGTGCCCAGTGGCTG TTCCTCGGCAAGGGCCTC GGCGCCAGCAACCAGTTC GAGGCCGGCGGCTTCATC	50	2 quartet	6048027	0.305151	0.013861	CDS	6047364	6049049	-	PA5372	*betA*
2469	GCCGGCAGACCCGCGCC	ACGCTGGAAAGCCGGCAG	50	2 quartet	3897792	0.286667	0.013984	CDS	3897391	3898191	+	PA3483	*PA3483*

	GAGGACCTGGCCCAGG CGGTCGCCGACACCCGC	ACCCGCGCCGAGGACCTG GCCCAGGCGGTCGCCGAC ACCCGCCAGCAACGCGAG CGCCTGCAACGCGAGTAC											
2389	GCCGGCGGCGACGGCA TCATGGTGATCGCCGAC CGCTTCGAGGGCCGCTA	GGCGCAGGGCGCCGGCGG CGACGGCATCATGGTGAT CGCCGACCGCTTCGAGGG CCGCTACAAGACCGTGTC GGGGAAGATCGTCAGCCA	50	2 quartet	1910368	0.299369	0.013988	CDS	1909698	1910681	-	PA1766	*PA1766*
306	GGCGCGCTGGCCTGCTT CCAGCGGGTGGTGGTGG TCAAGCGGCTGCCGAA	CGAACAGATCGGCGCGCT GGCCTGCTTCCAGCGGGT GGTGGTGGTCAAGCGGCT GCCGAAGACCCGCTCCGG GAAGATCCTCCGCGCGGT	50	2 quartet	3999347	0.573333	0.014081	CDS	3999208	4001094	-	PA3568	*PA3568*
171	GCTTCCTCGGCCTGACC TCGGGGATGCCGGTGGA GAAGATCGTCAAATCC	ATCGCCGCCGGCTTCCTC GGCCTGACCTCGGGGATG CCGGTGGAGAAGATCGTC AAATCCTTCCAGGACGGC TTCGGCGGCGTGCTCGGC	50	2 quartet	2560932	0.278834	0.014221	CDS	2560762	2562114	+	PA2322	*PA2322*
811	GCGCAAGCCGCTGGAA CTGGCCGAGCCCGGCGA GCGCCGGCTCAAGCTGT	TGCCGCCGGCGCGCAAGC CGCTGGAACTGGCCGAGC CCGGCGAGCGCCGGCTCA AGCTGTACGTACGCACCC AGCTGCCGAACCTGATCA	50	2 quartet	416596	0.432292	0.014361	CDS	416009	417406	-	PA0372	*PA0372*
867	GCGCGTCGAGAAGGGC GCGGCCCAGGTCGCCGA	TGGCCCAGGAGCGCGTCG AGAAGGGCGCGGCCCAGG	50	G ≥ 40 %	4182539	0.270634	0.014684	CDS	4182075	4182770	-	PA3731	*PA3731*

	GTACGAGCAATACGCG<br>A	TCGCCGAGTACGAGCAAT<br>ACGCGATCAAGGCACTGG<br>AGGCCGGCAACGAGGAGC												
128	AGTCGTTGCAGGTCAAG<br>GACTCGGTGGCGCGGGT<br>CGATTGCCTGTTGTAC	GATTGGGAACAGTCGTTG<br>CAGGTCAAGGACTCGGTG<br>GCGCGGGTCGATTGCCTG<br>TTGTACGGCGCCCTGCTG<br>GTGGTGTTGCTCCGCCAC	50	2 quartet	1006988	0.471667	0.014965	CDS	1006860	1007219	+	PA0921	*PA0921*
493	CCTGTTCCGCAGCCTGG<br>TCCGCCCGGCCAGTTGG<br>CGGGTCACGCCGCTGG	CCCTGGAGACCCTGTTCC<br>GCAGCCTGGTCCGCCCGG<br>CCAGTTGGCGGGTCACGC<br>CGCTGGTGGCGAACATCG<br>AGAAGAGCGGCCTGGACG	50	2 quartet	3600946	0.576471	0.015327	CDS	3600454	3601599	+	PA3211	*PA3211*
2574	GGTTCGACGACTGGATC<br>GCCCGTCGGCGCAACGG<br>CGAGCCGGTGGCCTAC	GCCAACGAGCGGTTCGAC<br>GACTGGATCGCCCGTCGG<br>CGCAACGGCGAGCCGGTG<br>GCCTACATACTTGGCCAC<br>CAGGGCTTCTGGAGCCTG	50	2 quartet	5232277	0.435995	0.015338	CDS	5231653	5232483	-	PA4664	*prmC*
79	CGCGGGGGCTCCGAGC<br>GGCGGCAAGAGCAAGC<br>TGAAGCTGATCCTGCTG<br>A	CACCCGCCGACGCGGGGG<br>CTCCGAGCGGCGGCAAGA<br>GCAAGCTGAAGCTGATCC<br>TGCTGATCGTGGTCGGCC<br>TGCTGCTGGCGATCGGCC	50	2 quartet	1572101	0.554348	0.015882	CDS	1572023	1572544	+	PA1442	*PA1442*
555	TCGTCGCGGGGCCTGCG<br>GTGGGGGGGTGGGCAT<br>GGCCTGCGAGCGGTACT	GCGACTCCCGTCGTCGCG<br>GGGCCTGCGGTGGGGGGG<br>TGGGCATGGCCTGCGAGC	50	long loops	4059357	0.297735	0.016565	CDS	4059018	4059911	-	PA3623	*PA3623*

		GGTACTCTGATCGGCCGT<br>TTTGCCTCAAACGGAAGT												
559	GCCCATGGGGCAGCCTG<br>CGGCGAGGCGGTGCTCA<br>AGGCCGTGGCCGAGGA	CGGGCCGCGCGCCCATGG<br>GGCAGCCTGCGGCGAGGC<br>GGTGCTCAAGGCCGTGGC<br>CGAGGACTTCCAGGTCGA<br>CGAGGTGCTGGAAATTCC	50	2 quartet	4062341	0.297474	0.016902	CDS	4061362	4062429	-	PA3626	*PA3626*
738	GCTTCCCGGTCGCCTAC<br>GTCGGCGACGTGGTCGG<br>CACCGGTTCCTCGCGC	AAGGCCAAGGGCTTCCCG<br>GTCGCCTACGTCGGCGAC<br>GTGGTCGGCACCGGTTCC<br>TCGCGCAAATCCGCCACC<br>AACTCGGTGCTGTGGTTC	50	2 quartet	1936907	0.268893	0.016926	CDS	1935035	1937644	-	PA1787	*acnB*
2521	CTTTTCGTGGTGGTCGG<br>CGACCATGGCTTCGGCA<br>GCCCCGAGCAGCTCAC	CAAGGACACCCTTTTCGT<br>GGTGGTCGGCGACCATGG<br>CTTCGGCAGCCCCGAGCA<br>GCTCACCGAGATGGACCT<br>GCACCGCTTCAACGTGCC	50	2 quartet	1839908	0.392305	0.017113	CDS	1838260	1840362	+	PA1689	*PA1689*
796	AGAAGTGGAACGGCGA<br>CGTCGCGGTCGGCTGGA<br>CCCCCGACGAGGACACC	TCGCGCTGGAAGAAGTGG<br>AACGGCGACGTCGCGGTC<br>GGCTGGACCCCCGACGAG<br>GACACCCTGATCGAACTC<br>ACCGCCGGCAAGGGCGAC	50	2 quartet	4249077	0.272193	0.01713	CDS	4247703	4249874	-	PA3790	*oprC*
93	AGAACATCCAGGAAGG<br>CGCGGTCACCGAAGGCT<br>ATTCGGCCGATCGCCAG	CGTGCCCGCAAGAACATC<br>CAGGAAGGCGCGGTCACC<br>GAAGGCTATTCGGCCGAT<br>CGCCAGACCGTCCTGCGC	50	2 quartet	5477046	0.319611	0.017275	CDS	5476945	5477478	+	PA4880	*PA4880*

		CTGCTCAACGAAGCGCTC											
762	TCGCCGAGGGCCAGCG GGTCCAGGTCGGCACGC CGCTGGCGCGCTCCGGC	TCGGTACTGGTCGCCGAG GGCCAGCGGGTCCAGGTC GGCACGCCGCTGGCGCGC TCCGGCAACACCGGCAAC AGCAGCGGGCCGCACCTG	50	2 quartet	6030489	0.254605	0.017473	CDS	6030351	6031250	-	PA5363	*PA5363*
607	TGGCACGCTGATCGCCG AACGCGGCCTGAAGCTG GCGGCCGATGAGGCGA	AAAACGACGCTGGCACGC TGATCGCCGAACGCGGCC TGAAGCTGGCGGCCGATG AGGCGAACAACTCCAAGG GGCGTATCGTCGCCAAGG	50	2 quartet	2774880	0.669759	0.017566	CDS	2761921	2778804	-	PA2462	*PA2462*
212	GCGGTGGAAAGCCTGG ATGGACGCACCCGTCGC GAGTGGCGTTTTTCCTA	CTGGGTGCTGGCGGTGGA AAGCCTGGATGGACGCAC CCGTCGCGAGTGGCGTTT TTCCTACAACGCGGTGAT GGAAGCCGAGCCCCAGGC	50	2 quartet	4252798	0.507222	0.017847	CDS	4252677	4253009	-	PA3793	*PA3793*
2459	GCGCGAGGCGGTGAAG AACGGCGTGGTACAAG CCATCGCCAGCCACCAC C	GCGAAGCCCTGCGCGAGG CGGTGAAGAACGGCGTGG TACAAGCCATCGCCAGCC ACCACCAACCCCACGAGG CGGACGCCAAGAACGCGC	50	2 quartet	443769	0.403465	0.017993	CDS	443419	444690	-	PA0401	*PA0401*
488	CTCGGCCCGCGCCGTTC CCAGCAGAGCCTGCTGG CGGTGCCGGGCGAGGG	GGAAATGCTGCTCGGCCC GCGCCGTTCCCAGCAGAG CCTGCTGGCGGTGCCGGG CGAGGGTACCCAGGTGCC GATCTGGCTACTCGGTTC	50	2 quartet	2802064	0.477851	0.01803	CDS	2801550	2802551	-	PA2483	*PA2483*

868	CGAGCCACGCACCTGGT TCAAGGTAGTCCGGCGC GTCGCCGGCGCCAACT	TCCACCGGATCGAGCCAC GCACCTGGTTCAAGGTAG TCCGGCGCGTCGCCGGCG CCAACTATGGGGCGCGCT ATTGTCGCCAGCGCTTCC	50	2 quartet	2340773	0.265238	0.018329	CDS	2340414	2341640	-	PA2127	*cgrA*
2459	TCCGCCTCGGCGCCCAT CGGCTGGACCGCGGCG ACGCCGAGGCGACCCTG	CGCCAGATGGTCCGCCTC GGCGCCCATCGGCTGGAC CGCGGCGACGCCGAGGCG ACCCTGTACTGCGCAATG GCCAAGCGCTTCGCCACC	50	2 quartet	813554	0.321839	0.018333	CDS	813329	814492	-	PA0746	*PA0746*
1090	GCACGCCGGCGCCCTCG GCTGGGTGGCGATGATC TCCATCGGCTCGCTCT	TCGGCCACGTGCACGCCG GCGCCCTCGGCTGGGTGG CGATGATCTCCATCGGCT CGCTCTACCACCTGATCCC GAAAGTCTTCGGCCGTC	50	2 quartet	1691665	0.307031	0.018735	CDS	1691327	1692754	-	PA1554	*ccoN1*
428	ATCGAGCCGGAGCAGT ACAACCCGGCCGGCAA GGACGAGTGGCAGGTC CT	TTGCCTGTTCATCGAGCCG GAGCAGTACAACCCGGCC GGCAAGGACGAGTGGCAG GTCCTCAATGTCGCCAAC TTCGAATGCGTGCCCGA	50	2 quartet	5845582	0.261638	0.018814	CDS	5844468	5846009	-	PA5192	*pckA*
2623	CATACTTGGCCACCAGG GCTTCTGGAGCCTGGAC CTGGAGGTCGCGCCAC	CGGTGGCCTACATACTTG GCCACCAGGGCTTCTGGA GCCTGGACCTGGAGGTCG CGCCACACACCCTGATCC CGCGCCCGGACACCGAAC	50	2 quartet	5232228	0.44774	0.019485	CDS	5231653	5232483	-	PA4664	*prmC*
135	GGCACCACTGCCATGGC	AAAGGGCGTGGGCACCAC	50	2 quartet	2212813	0.61	0.019531	CDS	2212677	2213309	+	PA2020	*mexZ*

	CGACCTGGCGGACGCCG CCGGGGTTTCTCGCGG	TGCCATGGCCGACCTGGC GGACGCCGCCGGGGTTTC TCGCGGTGCGGTCTACGG CCACTACAAGAACAAGAT												
491	GCAGCGGCGTGGGGGA GGCCTTCCCGCCGTTGC AGGGGCAGGGCGAGAT G	GGTCCGGCCGGCAGCGGC GTGGGGGAGGCCTTCCCG CCGTTGCAGGGGCAGGGC GAGATGTACCTGAGCAAC CAGTTGCGCGCCTGGCAG	50	2 quartet	2800938	0.365623	0.019934	CDS	2800782	2801435	-	PA2482	*PA2482*
1180	GCTCCTGAACAGAAGTA AGAGAAAGGTTTCTGCC GAGGTGGTGGAATTGG	TGTACCGGCGGCTCCTGA ACAGAAGTAAGAGAAAG GTTTCTGCCGAGGTGGTG GAATTGGTAGACACGCTA CCTTGAGGTGGTAGTGGC C	50	2 quartet	5332322	0.701697	0.020231	tRNA	5332254	5332339	-	PA4746.2	*PA4746. 2*
173	CTGCTGGTGGTCGGCCT GATCGTCGCGCTGATCG AGGTGGCGCTGTTCAG	GTTCCTGGCCCTGCTGGTG GTCGGCCTGATCGTCGCG CTGATCGAGGTGGCGCTG TTCAGCTACCTCGGCCGC ATCGTCGATCTCGCCCA	50	G ≥ 40 %	3615105	0.47224	0.020708	CDS	3614930	3616762	+	PA3228	*PA3228*
815	CGGCCCGCTGGTGGAAG CCCTGGCCAACGGCGTG ACCCAGCGCGACGTAC	TGGTCGCCGCCGGCCCGC TGGTGGAAGCCCTGGCCA ACGGCGTGACCCAGCGCG ACGTACTGCGCGGCAAGA TGCACCGCCTTTCGCCGC	50	2 quartet	5255515	0.476653	0.020968	CDS	5254738	5257584	+	PA4686	*PA4686*
7294	AGTATGAGTTCGCAGCT	TGTTTCCGGTAGTATGAGT	50	G_3L_{1-7}	2762288	0.337079	0.021097	CDS	2761921	2778804	-	PA2462	*PA2462*

	TGGGGCAGTGGGGGATT TGGGTCGGCTGGGTGG	TCGCAGCTTGGGGCAGTG GGGGATTTGGGTCGGCTG GGTGGGGGAGGTAAAGGT TATGTCGATATTCTTTC												
619	GATCTGGGGCGGCCCGC CCGGTTCGCCGGAAGAG GACGGCGACCGCTACA	ATGGGCCGGAGATCTGGG GCGGCCCGCCCGGTTCGC CGGAAGAGGACGGCGACC GCTACATCGAGATCTGGA ACAACGTGTTCATGCAGT	50	2 quartet	987436	0.484501	0.021103	CDS	986818	989442	+	PA0903	*alaS*
133	GCCGCTGGGGCAGTCCA TCGACGTGTTCGAGGAC GTGCGCGGTAGCGCCG	TCGAGCGCCTGCCGCTGG GGCAGTCCATCGACGTGT TCGAGGACGTGCGCGGTA GCGCCGATATCAACGACA TCACCTCGCGGGCCATCG	50	G ≥ 40 %	4455544	0.555556	0.021612	CDS	4453289	4455676	-	PA3974	*ladS*
180	AGGCGAAAGCCGCCGT GGTGGAAGAGGAGCTG CCCTCGGTCGAAGCCAA G	CCCGGGAAGAAGGCGAA AGCCGCCGTGGTGGAAGA GGAGCTGCCCTCGGTCGA AGCCAAGCAGAAAGAGC GTGACGCCCTCGCCAAGG CG	50	G ≥ 40 %	5950172	0.425283	0.021629	CDS	5950034	5950351	-	PA5285	*sutA*
2367	GATCGAAGCGGTGGTG GTGCCGGAGACCTGGTT CTTCCGCTACCCGGAGT	TGCAGGCGCTGATCGAAG CGGTGGTGGTGCCGGAGA CCTGGTTCTTCCGCTACCC GGAGTCGTTCACCACCCT CGCCAGGCTGGCCTTCG	50	2 quartet	4149977	0.292343	0.022274	CDS	4148931	4150199	-	PA3706	*wspC*
850	CCTGGCGCTGGCGGCGA	GTGGCGCCGCCCTGGCGC	50	2 quartet	5253607	0.2875	0.022494	CDS	5252758	5254056	+	PA4684	*PA4684*

	TCCGGCGCAAGGGCCTG GACGCCGTGCCGCAGG	TGGCGGCGATCCGGCGCA AGGGCCTGGACGCCGTGC CGCAGGCGGCGATGCCGC TGTTCACCCGGCCGCAGA											
1123	TGGTGACTCCGCGCGAG CGGCGCATGCTGCAGGT GATCGAGCGGGTCACC	GCGCTGTTGCTGGTGACT CCGCGCGAGCGGCGCATG CTGCAGGTGATCGAGCGG GTCACCGGGCAGAAGGTC GGCGAAGTCCGCCTGCCG	50	G ≥ 40 %	3194465	0.311828	0.023308	CDS	3193886	3195589	-	PA2840	*PA2840*
938	GCCCGCCCGGCGCAGGC GCTGGCGCAGCTGGCCA AGGGTTTCGCCGGCGA	TGGCCTGCATGCCCGCCC GGCGCAGGCGCTGGCGCA GCTGGCCAAGGGTTTCGC CGGCGAGATCCGGGTACG CCTGGCCGACAGTGAGGC	50	2 quartet	3993474	0.338501	0.023326	CDS	3991541	3994411	-	PA3562	*fruI*
1247	GGCGGCCTGGGCAACG ACTGGACCCCGGTGCGC GCGCTGGGCTCCTACAT	GAAATTCGCCGGCGGCCT GGGCAACGACTGGACCCC GGTGCGCGCGCTGGGCTC CTACATCAAGGGCACCAA CGGCAAGTCCCAGGGCGT	50	2 quartet	1253063	0.351375	0.02335	CDS	1251418	1254309	-	PA1156	*nrdA*
2892	TGGCGGTCGGCTTCGTC GGCATGATCGGCTCCTT CATCGTGCGCAGCTAC	TGGTGGCTCATGGCGGTC GGCTTCGTCGGCATGATC GGCTCCTTCATCGTGCGC AGCTACAACCAGGACGTC GACTACTACGTCCAGCCC	50	2 quartet	1430971	0.266462	0.023691	CDS	1429082	1431058	+	PA1318	*cyoB*
431	GCGGGGCAGGGCGGCA TGGCCGCGATCCTTGGC	GGCGGTTCCGGCGGGGCA GGGCGGCATGGCCGCGAT	50	2 quartet	3326653	0.390085	0.023803	CDS	3326145	3327083	-	PA2968	*fabD*

	CTGGAAGACGCCGATGT	CCTTGGCCTGGAAGACGC CGATGTATTGGCGGCCTG TGCCGAGGCGGCCCAGGG											
189	AGCTGCTCGACGCACCG CTGGCGAAGATCGGCG GCAAGGGCCTGTTCGTC	CGCGGCGACAAGCTGCTC GACGCACCGCTGGCGAAG ATCGGCGGCAAGGGCCTG TTCGTCAAGGAACTGGAA ACCGCCCTGCTCGAAGGC	50	2 quartet	5922247	0.294727	0.024324	CDS	5921494	5922435	-	PA5260	*hemC*
1041	CGGAGTCGGTGGGCGA GACCGGGGCCGCGGTG AGTCGCCTGGCCCAGGA C	CAGCGCCTGTCGGAGTCG GTGGGCGAGACCGGGGCC GCGGTGAGTCGCCTGGCC CAGGACAGCAACGAGATC GGCGGCGTGGTCGATGTG	50	bulges	1698907	0.26125	0.024422	CDS	1698382	1699947	-	PA1561	*aer*
1850	CAGCAACGGCTCCTACC GCGACATGGCGGTGGCC GGCCTGCGCAGCCTGT	GCGCCTCGTTCAGCAACG GCTCCTACCGCGACATGG CGGTGGCCGGCCTGCGCA GCCTGTCCGAGGCCGACA AGCGGACCCAGGCGCTGA	50	2 quartet	415557	0.283753	0.024589	CDS	414529	416016	-	PA0371	*PA0371*
3008	AGGTGTCCGGCCGCCTG CAGGCGCTGATGGAGC GGGTCAACAGCATGGA G	CACGAACAGGAGGTGTCC GGCCGCCTGCAGGCGCTG ATGGAGCGGGTCAACAGC ATGGAGCAGGATGCCAAG GCGTTCCACAGCCACCTG	50	2 quartet	6178570	0.322467	0.02462	CDS	6178036	6180051	-	PA5487	*PA5487*
2069	GAACAGGCGCTGGGGC CCTGCGGCCCATGGCAG GAGCACGGCCAGACCCT	CGCCTGGCTGGAACAGGC GCTGGGGCCCTGCGGCCC ATGGCAGGAGCACGGCCA	50	2 quartet	1258355	0.308524	0.02474	CDS	1258087	1258470	-	PA1160	*PA1160*

		GACCCTCAAGTGCACCGC CCGCGGCGAGCACGGCGC												
506	GGTGGCGGTGCTGGTCG GCCTGCTGGTGTTGCAG CTGATGCCGGCGATCC	CGGTGGCGCTGGTGGCGG TGCTGGTCGGCCTGCTGG TGTTGCAGCTGATGCCGG CGATCCCGGGGTCGGCGG GCAGCGGCTCGCTGCGCG	50	2 quartet	3745444	0.625	0.025047	CDS	3744901	3746067	+	PA3336	*PA3336*
2448	TGCCGAAGAGGAAGCG CGCAAGGCCGAGGAAG CTGCGCGTGCCAAGGCT G	CCCGCCAGCGTGCCGAAG AGGAAGCGCGCAAGGCCG AGGAAGCTGCGCGTGCCA AGGCTGCCCAGGAAGCAG CGGCTACTGCCGGTGCCG	50	G ≥ 40 %	5329511	0.317639	0.025425	CDS	5327427	5329949	-	PA4744	*infB*
1170	GCGGCCTGCGACCTGGC GCGGATGGCCGGGTTCG AGCCGTCCGGGGTGAT	GCACACCGAGGCGGCCTG CGACCTGGCGCGGATGGC CGGGTTCGAGCCGTCCGG GGTGATCTGCGAGGTGAT GAACGACGACGGCAGCAT	50	2 quartet	4535323	0.314228	0.025599	CDS	4534710	4535807	-	PA4054	*ribB*
1236	GACCGGATCTTCATCGA GGCCGGCTTCGAATGGC GCGAGCCGGGCTGTTC	GGAAGGCCTGGACCGGAT CTTCATCGAGGCCGGCTT CGAATGGCGCGAGCCGGG CTGTTCCATGTGCCTGGCG ATGAACCCGGACCGGCT	50	2 quartet	3503473	0.541935	0.026474	CDS	3503292	3504716	-	PA3121	*leuC*
210	GTGAGCGCGGACTGGTG GTGCTCGGGTTCCCCTG CAACCAGTTCGGCAAG	GAAAAATACCGTGAGCGC GGACTGGTGGTGCTCGGG TTCCCCTGCAACCAGTTCG GCAAGCAGGAACCGGGCG	50	2 quartet	3180233	0.290262	0.027006	CDS	3179957	3180442	-	PA2826	*PA2826*

		ACGAGGGCGAGATTTCG											
5314	GGTCGGGCCGCGCCTGA AGGTGGTGGTGCCGGTG CTGCCGAGGATCAGCA	AGGCGGCCAAGGTCGGGC CGCGCCTGAAGGTGGTGG TGCCGGTGCTGCCGAGGA TCAGCAACCACACCGATT TCGATCCGCTGCGCCTGC	50	2 quartet	1389034	0.335649	0.027269	CDS	1388242	1389714	+	PA1277	*cobQ*
350	GGACGCTGGGTGCTGGC GGTGGCCGGTACCCACG GCAAGACCACCACCAC	CGTGCTGCAAGGACGCTG GGTGCTGGCGGTGGCCGG TACCCACGGCAAGACCAC CACCACCAGCATGCTCGC CTGGGTCCTGGAGCACGC	50	2 quartet	4499491	0.306818	0.02738	CDS	4498488	4499843	-	PA4020	*mpl*
2950	CCGGCGCTGGACCTCAC CGTGCGCCTGCGCCAGC TGGAACTGGGGGCGGT	TACCCTGAACCCGGCGCT GGACCTCACCGTGCGCCT GCGCCAGCTGGAACTGGG GGCGGTTAACCGCAGCGA GGCGGTGCTGACCCAGGC	50	2 quartet	3991462	0.315954	0.02738	CDS	3990597	3991541	-	PA3561	*fruK*
434	CAGGTGGACCTCATCGG CGAAGGCTTCGACGCCG CCATCGGCGGCGGTTT	CGACAATCGCCAGGTGGA CCTCATCGGCGAAGGCTT CGACGCCGCCATCGGCGG CGGTTTCGAACTGCCTCCC GGGGTGGTGGCGCGCAA	50	2 quartet	206515	0.5	0.027549	CDS	206022	206954	-	PA0181	*PA0181*
2687	CGCCGACATCGTCGGCC AGCTCGGCGCCATCGGT TTCTACGGCCTGCCGC	CCGCGAGCAACGCCGACA TCGTCGGCCAGCTCGGCG CCATCGGTTTCTACGGCCT GCCGCTGGACTACCTGGA AAGCTTCCTCAAGCAGG	50	2 quartet	414720	0.307292	0.027782	CDS	414529	416016	-	PA0371	*PA0371*

1461	AGATCAACGTCGGCGGGCAGATGGTCGGCCTCGGCGGGCAGATCCACAAC	AACGGCGGACAGATCAACGTCGGCGGGCAGATGGTCGGCCTCGGCGGGCAGATCCACAACTACGAGCGGGTCGAGGCGTTCAACACCTCC	50	2 quartet	3458473	0.339481	0.028023	CDS	3457969	3459933	-	PA3082	*gbt*
6244	TGGACGGCATGCTCAGCGAGTCGCTCCTGCAGCCGGTGGCGTTGGAGCGC	GCCGGCCTGCTGGACGGCATGCTCAGCGAGTCGCTCCTGCAGCCGGTGGCGTTGGAGCGCCTGGAGGTCGACAGCGTGATTCTCCAGCGG	50	G ≥ 40 %	1181857	0.54375	0.028219	CDS	1177613	1182697	+	PA1091	*fgtA*
1644	GCGGTCCGCTGGCGCGCCTGCGCGACGGCGACCGGGTGCGGGTGGATGGG	GCCATCGCCGGCGGTCCGCTGGCGCGCCTGCGCGACGGCGACCGGGTGCGGGTGGATGGGGTGAACGGCGAGTTGCGGGTGCTGGTCGAC	50	bulges	3585660	0.463335	0.028323	CDS	3585477	3587303	-	PA3194	*edd*
4547	GAGCCTGGCCGCCCAGGCCGGCCTGGAGGCCGGGCAGGAGCTGCTGGCGG	TGGCGCCGGAGAGCCTGGCCGCCCAGGCCGGCCTGGAGGCCGGGCAGGAGCTGCTGGCGGTGGATGGCGAGCCCGTCACCGGCTGGAACG	50	2 quartet	4088422	0.284211	0.029158	CDS	4087526	4088878	-	PA3649	*mucP*
3220	TCGCCGAGGGCGGCAAGATCGAGCCGCCGCCGGAACTGGGCAGCGCGCGC	TGCGTACTGCTCGCCGAGGGCGGCAAGATCGAGCCGCCGCCGGAACTGGGCAGCGCGCGCAAGCTGCCGAAGGATTTCTCCGACCTCGCC	50	G ≥ 40 %	5575330	0.407563	0.029406	CDS	5575015	5576028	-	PA4966	*PA4966*
362	ATGCTGAGCATCAGGGC	CGTCGAGGCGATGCTGAG	50	2 quartet	5868392	0.703297	0.029522	CDS	5868181	5871057	-	PA5213	*gcvP1*

	CGAGATCGGCAAGGTG<br>GAGAGCGGCGCCTGGC<br>C	CATCAGGGCCGAGATCGG<br>CAAGGTGGAGAGCGGCGC<br>CTGGCCGGCGGAGGACAA<br>CCCGCTGAAACGGGCGCC											
673	CGGCGCGCCGAAGGCA<br>CGGCGGACCCTGCGCGA<br>CCTGATCCGCGACTATC	CGGCGGAAGTCGGCGCGC<br>CGAAGGCACGGCGGACCC<br>TGCGCGACCTGATCCGCG<br>ACTATCGCCTGGCCGGCC<br>GCGAACGGCGCCTGCAGG	50	2 quartet	4300789	0.259626	0.029792	CDS	4300117	4301949	+	PA3839	*PA3839*
1319	GCCTCCGAGCACCAGGC<br>GCAGGAAATCGCCGGC<br>GCCTCGGCGGCGATCAA	CCTGGCCGAAGCCTCCGA<br>GCACCAGGCGCAGGAAAT<br>CGCCGGCGCCTCGGCGGC<br>GATCAACGAAATGGCGGT<br>GTCGATCGACCAGGTATC	50	2 quartet	452448	0.292852	0.031147	CDS	451130	453178	+	PA0411	*pilJ*
709	CCGCGTACTGAAGGGCA<br>TGCAGGAAGGCAACGC<br>GGCGATGAGCATCTCCA	GCCTGTTCGGCCGCGTAC<br>TGAAGGGCATGCAGGAAG<br>GCAACGCGGCGATGAGCA<br>TCTCCAAGGTGACCAACG<br>CCGAGGCGGTGGATCGCC	50	2 quartet	451838	0.472754	0.002165	CDS	451130	453178	+	PA0411	*pilJ*
1637	CTGGTTGGGCGGTGCTC<br>GACGTGAGGCGGTTTCA<br>GGAGTGGTTGAGCATG	AGTTGCGCGCCTGGTTGG<br>GCGGTGCTCGACGTGAGG<br>CGGTTTCAGGAGTGGTTG<br>AGCATGTCTGACGAGTGG<br>AAGTCGGAAACGCCGAAG	50	2 quartet	3330668	0.643678	0.031256	CDS	3330663	3331355	-	PA2974	*PA2974*
792	GCGAGAAGCTCAACGA<br>CCCGGCGGCGGTGGCCA	CACTACGCCAGCGAGAAG<br>CTCAACGACCCGGCGGCG	50	2 quartet	28437	0.262218	0.031631	CDS	27646	28632	+	PA0026	*plcB*

	ACCTGGTGGGGCGCTAC	GTGGCCAACCTGGTGGGG CGCTACGACCCGAGCAAG AGCATCCGCGACCTGCTC											
3123	CGGCGAGGCGCCCGGC CTGGTGGTCAGCCCGAA CCCGTTCTACCAGATCT	GCAGCGAGCCCGGCGAGG CGCCCGGCCTGGTGGTCA GCCCGAACCCGTTCTACC AGATCTACGAAGGCGCGG CGCTGCTCGCCGGAGCCG	50	2 quartet	4099246	0.56379	0.031975	CDS	4098844	4100052	+	PA3659	*PA3659*
163	GGCGCCGGCGGCGGAG CCCTGGCGGCCGGCGCC ATCGGCCTGCTGCTGGG	CCTGCTTTCCGGCGCCGG CGGCGGAGCCCTGGCGGC CGGCGCCATCGGCCTGCT GCTGGGAAACAAGAAGGC GCGCAAGTTCGGCGGCAA	50	2 quartet	4157655	0.36036	0.032015	CDS	4157126	4157821	-	PA3712	*PA3712*
755	CACGAAGAGGAACTGG CCAGGCGCGAACAGGA CGCCCGCGGGCAACTGG A	CGCCCGGCAGCACGAAGA GGAACTGGCCAGGCGCGA ACAGGACGCCCGCGGGCA ACTGGACATCCTGCGCAG CGAAGTGCTCAGCCTGCA	50	2 quartet	3469673	0.616667	0.032167	CDS	3468988	3470427	-	PA3091	*PA3091*
603	ACGCCGATGCGCTGGCC GCCACCCGGCGCGCCCT GGATGGCGCCCAGGAA	GCCGATGCCGACGCCGAT GCGCTGGCCGCCACCCGG CGCGCCCTGGATGGCGCC CAGGAACATGCCCTGGCC ATCGAGAGCGGCGTCGCC	50	2 quartet	100726	0.544256	0.032364	CDS	100124	101158	+	PA0082	*tssA1*
1039	ACCCATTCGGCCGCGGA ACTCCCGGTCCGGGCCA GCCGGATACGCCTGGC	AATGAGCGATACCCATTC GGCCGCGGAACTCCCGGT CCGGGCCAGCCGGATACG	50	2 quartet	4990343	0.347308	0.032668	CDS	4990285	4990824	+	PA4458	*PA4458*

		CCTGGCGATCTTCGACGT CGACGGCGTGCTGACCGA											
160	GCCCGCCGGAGTTGCGC GAGGCGGTAGGTAGGC ATATCGCCGCCGGGCAC	GATTTCGACGGCCCGCCG GAGTTGCGCGAGGCGGTA GGTAGGCATATCGCCGCC GGGCACAACCAGTACGCG CCGATGACCGGCTTGCCG	50	2 quartet	4257227	0.280228	0.033188	CDS	4256240	4257388	-	PA3798	*PA3798*
672	AAGCCGTGGAGAACGG CGAGGTCGACATGGCGC TGATCAACAACTACTAC	ACCGCCATGAAAGCCGTG GAGAACGGCGAGGTCGAC ATGGCGCTGATCAACAAC TACTACTGGTACACCCTG AAGAAGGAAAAGGGCGA G	50	2 quartet	5258367	0.29378	0.033245	CDS	5257696	5258703	+	PA4687	*hitA*
1306	CCTGGTCTACCCGGACC GGCCGATCGTCGCGGTG TGCGGCGATGGCGGCT	TGGCCGCGCACCTGGTCT ACCCGGACCGGCCGATCG TCGCGGTGTGCGGCGATG GCGGCTTCATGATGAACA GCCAGGAACTGGAAACCG	50	2 quartet	4676620	0.325832	0.033419	CDS	4676282	4677925	-	PA4180	*PA4180*
958	CTTCTACCGCCAGGCCC AGGCCGATGGCGTCGA ACTGGTCGTCGGTCCAC	CGCTAGATGACTTCTACC GCCAGGCCCAGGCCGATG GCGTCGAACTGGTCGTCG GTCCACTGGAGAAGCCGC TGGTCAAGCAACTCGCTA	50	2 quartet	4958666	0.269928	0.033828	CDS	4957709	4959523	+	PA4423	*PA4423*
372	TGCTCGGCCCGGTCGGC GGCGGTAAATCCTCCCT GGCGGAAAAACTCAAG	ATCCTTTACCTGCTCGGCC CGGTCGGCGGCGGTAAAT CCTCCCTGGCGGAAAAAC	50	2 quartet	648281	0.459459	0.033877	CDS	646730	648652	-	PA0588	*PA0588*

		TCAAGCAACTGATGGAGA AGGTGCCCTTCTACGCG												
601	CCTGCTGCTGAGCGCGG TGGGACCGGCCTGGGTG TTCCTGTTCAACAGCT	CCCTCGGCGGCCTGCTGC TGAGCGCGGTGGGACCGG CCTGGGTGTTCCTGTTCAA CAGCTTCTGCTACATGGC CCTGATCTGGGCGATCT	50	2 quartet	3876796	0.381637	0.033984	CDS	3876196	3877911	+	PA3465	*PA3465*
281	GTCGGCATGGACGTGCG CTGGGACGGCGCTACCA TGAGCGTCGACGATAT	GTTCGTCCGGGTCGGCAT GGACGTGCGCTGGGACGG CGCTACCATGAGCGTCGA CGATATGATCAACGAAGG CGTGCGTCGCGCCTACAA	50	2 quartet	4861933	0.313364	0.0342	CDS	4861653	4863176	+	PA4333	*PA4333*
4273	TCGCGCGTCTGCACGGG CTGAGCGAGGCCGGAG TGGTCTACGCTCAACAG	TGCCAGGCCGTCGCGCGT CTGCACGGGCTGAGCGAG GCCGGAGTGGTCTACGCT CAACAGAACCCGGCGGTG ATCGACCAGGGCGTGTTC	50	2 quartet	983601	0.458057	0.034718	CDS	982885	984231	+	PA0899	*aruB*
3551	CCGCGTCGCGACATGGA CCCTCAGGCGCTGGAGG AACTGGCGCAGTCGAT	CAAGTACCAGCCGCGTCG CGACATGGACCCTCAGGC GCTGGAGGAACTGGCGCA GTCGATCAAGGCCCAGGG CGTGATGCAGCCCATCGT	50	2 quartet	6255645	0.397485	0.035005	CDS	6254972	6255844	-	PA5562	*spoOJ*
154	CGATCGCAGCGGCGGCC TGAAGCTGGCCCAGGCG TTGACCGCCGCGATGG	TGCTGGAGCGCGATCGCA GCGGCGGCCTGAAGCTGG CCCAGGCGTTGACCGCCG CGATGGAAGGGGTCGGTC	50	2 quartet	1672638	0.439286	0.035081	CDS	1672485	1673081	+	PA1534	*recR*

		ATTGCCGGCAGTGCCGTA											
398	GCCAGGGCACTGTCCGG CCGGGTTAGCCGGGGTG GCGTGGTGGCGTTCAT	GCTGCGCCTGGCCAGGGC ACTGTCCGGCCGGGTTAG CCGGGGTGGCGTGGTGGC GTTCATGAGTTCGCAGAT GGCCAGCCTGGCGCTGGG	50	2 quartet	4558699	0.498747	0.036251	CDS	4558302	4558991	+	PA4079	*PA4079*
169	CGGCCAGGGCGCCGGC GCCGGCAGCGCCGGTG GATCGCCTGTGGCAGAC C	CAGGTCGCGGCGGCCAGG GCGCCGGCGCCGGCAGCG CCGGTGGATCGCCTGTGG CAGACCGCCCAGGACCTG CTGCGCGGCAGCGGGCGG	50	2 quartet	4540963	0.346552	0.036296	CDS	4540783	4541139	-	PA4062	*PA4062*
652	GCCTGGCGGTGCTGGCG CTCGCCGCGCTGTTCGC CGCCTGGCGCTTCGAG	GCCCGCCTGGGCCTGGCG GTGCTGGCGCTCGCCGCG CTGTTCGCCGCCTGGCGCT TCGAGGTATCGCTGGTGC TGGGCATGGCCTGCCTG	50	G ≥ 40 %	4066756	0.690141	0.036543	CDS	4066103	4067329	+	PA3631	*PA3631*
1255	CGAGCACAATTTCGCCA ACGGCTGGGTCGGCAA GGTGCAACTCGATCACA	TCGCCAACCTCGAGCACA ATTTCGCCAACGGCTGGG TCGGCAAGGTGCAACTCG ATCACAAGATCAACGGCT ACCACGCGCCCCTCGGCG	50	2 quartet	2656441	0.276598	0.036655	CDS	2655187	2657634	+	PA2398	*fpvA*
114	GGCGGCCGGAAGACAA GCACCAGGGCGGCCTGT GGGAGTTTCCCGGGGGC	CTGATCGCCCGGCGGCCG GAAGACAAGCACCAGGGC GGCCTGTGGGAGTTTCCC GGGGGCAAGGTGGAGGA CGGCGAGCCGGTGCGCGC	50	2 quartet	4931582	0.496464	0.036697	CDS	4930748	4931695	-	PA4400	*mutT*

		G											
167	CTTCCGAACAGCTGGCC<br>ATGGTCCTCCAGGTCCC<br>CATGGCCTACCTCTAT	AACATGCGGACTTCCGAA<br>CAGCTGGCCATGGTCCTC<br>CAGGTCCCCATGGCCTAC<br>CTCTATTGCCCCGAGGAC<br>GAGCTGGCCGAGCTCATC	50	2 quartet	3138044	0.652826	0.037617	CDS	3137850	3138194	+	PA2780	*bswR*
548	TGGGTTGGGCCGGGTGG<br>CCTACGCCGACGCCCGC<br>CAGCACCGCGACATCC	GGGCGGCCCGTGGGTTGG<br>GCCGGGTGGCCTACGCCG<br>ACGCCCGCCAGCACCGCG<br>ACATCCTGCAATCGCTGG<br>TCCGCGCGCTGAACGGCT	50	2 quartet	4868285	0.833333	0.037681	CDS	4867767	4868846	-	PA4339	*PA4339*
975	GCGGCGCCCTGCAGGGC<br>GGCCTGCCGGTGCTGTC<br>GGTGGCCACCGGCTCC	GAACTCTGCCGCGGCGCC<br>CTGCAGGGCGGCCTGCCG<br>GTGCTGTCGGTGGCCACC<br>GGCTCCTACGACACCGCG<br>ACCAACCTGAACCGGATG	50	2 quartet	910558	0.258893	0.038278	CDS	909418	911532	-	PA0835	*pta*
2442	AGCAGCTGGCGGTAAAT<br>TCGAGGACTAAATGGCT<br>AAGCAAGGTGCTCTCT	CGGCTATCGAAGCAGCTG<br>GCGGTAAATTCGAGGACT<br>AAATGGCTAAGCAAGGTG<br>CTCTCTCTGCGCTAAGCA<br>ACGGCGGTCTGTCCGAGC	50	2 quartet	4758431	0.348665	0.038671	CDS	4757124	4758452	-	PA4243	*secY*
1272	ACCCCGCCGTCCTGGCT<br>GGAGGGCGGCCAGGCG<br>CGTTTCCTGCTCGGCAC	CTTCCTGCTGACCCCGCCG<br>TCCTGGCTGGAGGGCGGC<br>CAGGCGCGTTTCCTGCTC<br>GGCACCGACGAACTGGGC<br>CGCGACCTGCTCTCGCG	50	2 quartet	5043340	0.550866	0.038764	CDS	5043090	5044001	+	PA4504	*dppC*

207	ACCGTGAGCGCGGACTG GTGGTGCTCGGGTTCCC CTGCAACCAGTTCGGC	TGGGAAAAATACCGTGAG CGCGGACTGGTGGTGCTC GGGTTCCCCTGCAACCAG TTCGGCAAGCAGGAACCG GGCGACGAGGGCGAGATT	50	2 quartet	3180236	0.298201	0.03884	CDS	3179957	3180442	-	PA2826	*PA2826*
4146	GGGCGGCTCGGTTTCAA CACCTGGCTGGGCGATC CCGGCCAGGACGCCAG	GCAGGGCGGCGGGCGGCT CGGTTTCAACACCTGGCT GGGCGATCCCGGCCAGGA CGCCAGGGATCTACTGCT GGCCCGCCAGTACGCCAC	50	2 quartet	108158	0.638462	0.039635	CDS	107182	108228	+	PA0089	*tssG1*
7477	GTTCAAGGTCGAGGAG GTTGGCGACAAGCCGCT TTCCAGCGACGAACTGT	GGGCGTTCGAGTTCAAGG TCGAGGAGGTTGGCGACA AGCCGCTTTCCAGCGACG AACTGTATCTCGACGAAG TGACCCTGAACGTTCCGG	50	2 quartet	5020755	0.26159	0.039764	CDS	5020402	5024952	-	PA4489	*magD*
1042	GACCCAGTTGATCAGCC TTTCGGTACCGGTTCTG GTGGGACTCTATCCGC	ACCAGGGACTGACCCAGT TGATCAGCCTTTCGGTACC GGTTCTGGTGGGACTCTA TCCGCTCGCCATCGTGCTG ATCGCCCTGAGCCTGT	50	2 quartet	1733589	0.328107	0.039973	CDS	1732545	1733858	+	PA1590	*braB*
3166	CAGCGGCCTGCGCGATC TCGGCGAAGGCAACTG GCCGCAGGCACTGCGTC	TGGCCGAGCACAGCGGCC TGCGCGATCTCGGCGAAG GCAACTGGCCGCAGGCAC TGCGTCACTTGCGGCGGG CGGCGGAGATGGGCGAGC	50	2 quartet	5919270	0.307143	0.040736	CDS	5918350	5919588	-	PA5257	*PA5257*
2488	ACGTGGCGCGCCTGATC	AAGAACGCCGACGTGGCG	50	2 quartet	4485625	0.30631	0.042003	CDS	4485349	4485816	-	PA4004	*PA4004*

	CGTCAGGAAGGCGAGG CCATGCTGGCGCGCGTG	CGCCTGATCCGTCAGGAA GGCGAGGCCATGCTGGCG CGCGTGCAGCCGGGGGAA CGGGTGGTGACCCTGGAG												
400	GCGGGTAGCCGCCGTGC TGCGTCGCCACGGGGGA ACGCGTCCCGCGCAGC	AACTGAAGGCGCGGGTAG CCGCCGTGCTGCGTCGCC ACGGGGGAACGCGTCCCG CGCAGCATGAGGTGCAGA CTTTCAACGACCTCAGCT	50	G ≥ 40 %	4513485	0.432579	0.042753	CDS	4513168	4513884	-	PA4032	*PA4032*
2080	CCTGTTCAAGATCACCA GCGAGGGTGGCGTGGCT GCTGGCGTACGCCGTA	GCGACATCGGCCTGTTCA AGATCACCAGCGAGGGTG GCGTGGCTGCTGGCGTAC GCCGTATCGAGGCGGTCA CCGGCGCGGCGGCGCTGG	50	2 quartet	988897	0.285985	0.043493	CDS	986818	989442	+	PA0903	*alaS*
346	CGCCGCCGGCATCCCGG TGTTCGCCTGGAAGGGC GAGACCGAGGAAGAGT	CCGCCATCGCCGCCGCCG GCATCCCGGTGTTCGCCT GGAAGGGCGAGACCGAG GAAGAGTACGAATGGTGC ATCGAGCAGACCATCCTC A	50	2 quartet	483770	0.334484	0.043535	CDS	482706	484115	-	PA0432	*sahH*
1070	CGCCGCGCTGGTGGCCC TGGGCGTGGAGAATGG CCATGACTGACATGCAC	TGGCCGCGGCCGCCGCGC TGGTGGCCCTGGGCGTGG AGAATGGCCATGACTGAC ATGCACGACGACATCCCC GCCGGCAGCCGTTGCGGC	50	2 quartet	839421	0.765366	0.04414	CDS	839407	840324	+	PA0771	*era*
2836	CTGGGTGGTCCTGGCCG	TGCTGTCCGCCTGGGTGG	50	2 quartet	1606513	0.306336	0.044192	CDS	1605088	1607061	+	PA1480	*ccmF*

	GTTTCCGCGACTTCCTC GACAAGACCCGGCACA	TCCTGGCCGGTTTCCGCG ACTTCCTCGACAAGACCC GGCACAAGGGCGTGCTGG CCGGCGCCCGCAGCCTGA												
460	CGAAGGCGTGGCGGTG CTGGAGAACGGCAACG TCGCGGTGACCGACGAG C	TGAGCAATCCCGAAGGCG TGGCGGTGCTGGAGAACG GCAACGTCGCGGTGACCG ACGAGCGCCGCAATACCC TGACCATCTTCCATGTCG	50	2 quartet	359390	0.942308	0.044272	CDS	358931	359920	+	PA0319	*PA0319*
1183	GCGCGCCCCGGGCTGGG ACTTCAAGCGGGTCAAC GGCGGCCTGCTGGTAC	GGCCAGCCGAGCGCGCCC CGGGCTGGGACTTCAAGC GGGTCAACGGCGGCCTGC TGGTACAGAGCCGCGACA TCGGCATGATCAAGGCCG	50	2 quartet	5450228	0.494274	0.044344	CDS	5449046	5450653	+	PA4854	*purH*
203	GAAGATGATCGAAGAG CTGCAAGTCGGCGCCCG CGAGGCGGTGGCCACC A	AGCAGATCCAGAAGATGA TCGAAGAGCTGCAAGTCG GCGCCCGCGAGGCGGTGG CCACCATGACCGAGAGCC AGCGCTACAGCCTGGAGA	50	2 quartet	4836860	0.474201	0.0448	CDS	4835264	4837153	+	PA4309	*pctA*
3142	ATCCGTCGCGCCAAGGT CGCCGCGGGCGAGGCC GGCGGCATCACCCAGCA	GCTCGACTACATCCGTCG CGCCAAGGTCGCCGCGGG CGAGGCCGGCGGCATCAC CCAGCATATCGGTGCCTA CCACGTCGAAACCGAGCG	50	2 quartet	5328817	0.294525	0.045944	CDS	5327427	5329949	-	PA4744	*infB*
1928	TTGGCACAGGCGGTTTC CCGGCGTGGTCGCCAAG	CGCTGAGCTGTTGGCACA GGCGGTTTCCCGGCGTGG	50	2 quartet	3104825	0.863289	0.04602	CDS	3104279	3104830	-	PA2743	*infC*

	ACTCGGAGTAATCATT	TCGCCAAGACTCGGAGTA ATCATTATTAAGCGTGAA ATGAGACAGGATAAGCGA												
1242	CCGCTGGTGCCCATCGG CCTGGGCGCCGGGCGCA TCGGCAACTTCATCAA	CTTCATCGCCCCGCTGGTG CCCATCGGCCTGGGCGCC GGGCGCATCGGCAACTTC ATCAACTCGGAACTGTGG GGCAAGGTCAGCGATGT	50	2 quartet	384160	0.273227	0.046218	CDS	383727	384527	+	PA0341	*lgt*
368	TGGAACCTCGGCACCTG GTACTTCGGCATCCCGG CCTCCAGCTCGCACAC	CGCCATCGCCTGGAACCT CGGCACCTGGTACTTCGG CATCCCGGCCTCCAGCTC GCACACCCTGATCGGCTC GATCCTCGGCGTCGGCCT	50	2 quartet	4815410	0.312859	0.046732	CDS	4815043	4816512	+	PA4292	*PA4292*
1878	CGCCCTGCCGCCGGCGA CCTCGGAACTGGAGGGC ATGCAGCGCCTGCTGT	CGGCGCTGGACGCCCTGC CGCCGGCGACCTCGGAAC TGGAGGGCATGCAGCGCC TGCTGTATCGCCACGCCT GCGACGCCGACCTGGTGC	50	2 quartet	974944	0.531046	0.04715	CDS	974482	975594	+	PA0891	*PA0891*
4834	CCTCGGCGCCGGGTTCA TCGGCGGGATAGTCGCC GGCTTCATCGCCGGCT	TCGCCGGGACCCTCGGCG CCGGGTTCATCGGCGGGA TAGTCGCCGGCTTCATCG CCGGCTATGCGGCGCGGG CCATCAGCCACGGGCTGA	50	2 quartet	3989578	0.337963	0.047577	CDS	3988838	3990595	-	PA3560	*fruA*
484	CTTCCATGGCACCCTGG ATACGCCGGACCCGGCG CATGCCAGGAACATCA	CCTTCGTGTCCTTCCATGG CACCCTGGATACGCCGGA CCCGGCGCATGCCAGGAA	50	2 quartet	1739991	0.394231	0.04771	CDS	1739508	1740233	+	PA1597	*PA1597*

		CATCAAGGGCGCGGTACT GGTCCTCGACGGTGCCT											
13233	GGCGGACGTGATGCAG GACGCCGAAGGGCGTTT CTGGCTGCTTGAAGTCA	GCTGGGGGCGGGCGGACG TGATGCAGGACGCCGAAG GGCGTTTCTGGCTGCTTGA AGTCAACACCGCACCGGG CATGACCGACCACAGCC	50	G ≥ 40 %	4942796	0.437477	0.047753	CDS	4942677	4943636	-	PA4410	*ddlB*
1379	CCAAGATCGTGGTCTCC GGCGGCCGCGGCATGC AGAACGGCGACAACTTC	CTGACCGCTGCCAAGATC GTGGTCTCCGGCGGCCGC GGCATGCAGAACGGCGAC AACTTCAAGATCCTCTAC GCCCTGGCCGACAAGCTG	50	2 quartet	3311092	0.317717	0.049085	CDS	3310792	3311721	-	PA2951	*etfA*
948	GTGGCGGCGTGATCTAT GGCGCTCGTGATCCGGC GGACATCGAGGCGATC	CTGATCTATGGTGGCGGC GTGATCTATGGCGCTCGT GATCCGGCGGACATCGAG GCGATCATCCGGCCGAAG ATGCTCAAGACCTTCCCG	50	G ≥ 40 %	3134657	0.447566	0.049241	CDS	3133710	3134993	+	PA2776	*pauB3*
602	CACCGCGCCGCGGAAAT GGCCGGGTTGGCGGTCG GCGACAGCAGTTGGCT	CTACGTCAGCCACCGCGC CGCGGAAATGGCCGGGTT GGCGGTCGGCGACAGCAG TTGGCTCAGCGCCCACCT CGGCAACGGCAGCTCGAC	50	2 quartet	912178	0.260064	0.049549	CDS	911595	912779	-	PA0836	*ackA*
247	CCGCGGCGTGGTGCAAC GCAGCCAGGCGGCCGC CGAAGCGACCGAGGAA T	AGCGCACCCGCCGCGGCG TGGTGCAACGCAGCCAGG CGGCCGCCGAAGCGACCG AGGAATACGTCGAGGACC	50	G ≥ 40 %	3115550	0.319191	0.049623	CDS	3115304	3115633	+	PA2754	*PA2754*

		ATCCCTGGCAGACCATCG											
1483	CTCGGCCAATCCGGAGA AGGCGGAAGAGGGCGG CAGCTTCCTCGACAACA	GGCTGTTCCGCTCGGCCA ATCCGGAGAAGGCGGAAG AGGGCGGCAGCTTCCTCG ACAACATCAACGCCGACT CCCTGCAGGTACTGGCCG	50	2 quartet	1944549	0.441603	0.049953	CDS	1943067	1944737	+	PA1794	*glnS*

Table S1C Comparison between this study and the Guo et al. Science 2017.

	**Difference/Similarity**	**This study**	**Guo et al. Science 2017 (1)**
Experiments	Similarities	Similar in preparing library and the test condition is K^+	Similar in preparing library and the test condition is K^+
	Differences	-	DMS and NAI experiments *in vivo*
Analysis	Similarities	RT reads >10	RT reads >10
	Differences	Ten-order filter (RTS score > 0.25, P > 0.05)	Fold enrichment >20
		Pattern mapping (multiple type of rG4)	The RT stops at G (similar to canonical rG4)
rG4 sites *in vitro*	Differences	168	14
Verification	Differences	-	Ectopic expression G3A2
		QUMA-1 staining	-
		Thioflavin T (ThT) ligand enhanced fluorescence assay	
		Circular Dichroism	-
		Point mutation and *lux* reporter assays	-
		Phenotypic experiments	-
