# Supplementary material for: RNA G-Quadruplex Structures Mediate Gene Regulation in Bacteria
Source: mBio. 2020 Jan 21;11(1):e02926-19. doi: 10.1128/mBio.02926-19 (PMC6974567; doi:10.1128/mBio.02926-19)
Supplement: TABLE S2 [file mBio.02926-19-st002.pdf]

Table S2. Strains, plasmids and primers used in this study

Strain(s) or Plasmid	Genotype or phenotype	Source or Reference
***E. coli* strains**		Lab stock
E. coli K12 MG1655 Wild type		Lab stock
DH5α	*endA hsdR*17 *supE*44 thi-1 *recA*1 *gyrA relA*1 D (*lacZYA-argF*) U169 *deoR* (ϕ80*dlacD* (*lacZ*) M15)	Invitrogen
***P. aeruginosa* strains**		
PAO1 Wild type		Lab stock
**Other strains**		
Klebsiella pneumonia ATCC13883		Prof. Chen Sheng 's Laboratory
Vibrio parahaemolyticus vp001		Prof. Chen Sheng 's Laboratory
Acinetobacter ATCC25922		Prof. Chen Sheng 's Laboratory
Salmonella typhimurium PY1		Prof. Chen Sheng 's Laboratory
Staphylococcus aureus		Prof. Chen Sheng 's Laboratory
Enterococcus faecalis (ATCC 29212)		Prof. Chen Sheng 's Laboratory
Bacillus cereus		Prof. Chen Sheng 's Laboratory
P. syringae		Lab stock

Plasmids		Lab stock
pMS402	Expression reporter plasmid carrying the promoterless *luxCDABE*; Kan^r,Tmp^r	(2)
pKD-*hemL*-ORF-WT-*lux*	pMS402 containing *hemL* promoter region and ORF, Kan^r,Tmp^r	This study
pKD-*hemL*-ORF-△rG4-*lux*	pMS402 containing *hemL* promoter region and ORF with mutated rG4 sites, Kan^r,Tmp^r	This study
pKD-*bswR*-ORF-WT-*lux*	pMS402 containing *bswR* promoter region and ORF, Kan^r,Tmp^r	This study
pKD-*bswR*-ORF-△rG4-*lux*	pMS402 containing *bswR* promoter region and ORF with mutated rG4 sites, Kan^r,Tmp^r	This study
pAK1900	*E. coli / P. aeruginosa* shuttle cloning vector, Amp^r, Cb^r	(3)
pAK1900-*bswR*-WT	over-expressed *bswR* vector, Amp^r, Cb^r	This study
pAK1900-*bswR*-△rG4	over-expressed *bswR* vector with mutated *rG4 sites*, Amp^r, Cb^r	This study
pAK1900- *bswR*-FLAG	over-expressed *bswR* FLAG vector, Amp^r, Cb^r	This study
pAK1900- *bswR*-△rG4-FLAG	over-expressed *bswR* FLAG vector with mutated *rG4 sites*, Amp^r, Cb^r	This study
pAK1900- *hemL* - FLAG	over-expressed *hemL* FLAG vector, Amp^r, Cb^r	This study
pAK1900- *hemL* -△rG4-FLAG	over-expressed *hemL* FLAG vector with mutated *rG4 sites*, Amp^r, Cb^r	This study
**Primer name**	**Purpose**	**Sequence (5'-3')**
PKD- *hemL* P+ORF -F1	For constructing the pKD-*hemL*-ORF-*lux*	TCGTCTTCACCTCGAGGGGATCC TCGCGCTCAAGAAGTTGG
hemL - mut -R1	For constructing pKD-*hemL*-ORF-△rG4-*lux*	TACCGGAAAGCGTACCtGCtTGATAGACtGGACCCGTCGGGGCCA

hemL - mut -F2	For constructing pKD-*hemL*-ORF-△rG4-*lux*	TGGCCCCGACGGGTCCaGTCTATCAaGCaGGTACGCTTTCCGGTA
PKD- *hemL* P+ORF -R2	For constructing the pKD-*hemL*-ORF-*lux*	GCGGCCGCAACTAGAggatcc CAACTTCGCAAACACCCG
bswR –P+ORF-F1 (*XhoI*)	For constructing the pKD- *bswR* -ORF-*lux*	TTctcgagGGAATGACCTCTGCGTGA
bswR – mut-R1	For constructing pKD- *bswR* -ORF-△rG4-*lux*	AGGTAGGCCATGGGGACTTGGAGGACCATGGCTAGCTGTTCGGAAGTC
bswR –mut-F2	For constructing pKD- *bswR* -ORF-△rG4-*lux*	GACTTCCGAACAGCTAGCCATGGTCCTCCAAGTCCCCATGGCCTACCT
bswR –P+ORF -R2 (*BamHI*)	For constructing the pKD- *bswR* -ORF-*lux*	TTggatccCAGTTCACTCCTTGTGCG
pAK1900- *bswR*-F	For overexpressing *bswR*	ACACTATAGAATACTCAAGCTT AACGGGACGGGAGTTGTA
pAK1900- *bswR*-R	For overexpressing *bswR*	GAGCTCGGTACCCGGGGATCC TCACAGTTCACTCCTTGT
pAK1900- *bswR* -F (*Hind*III)	For overexpressing BswR-FLAG	ACACTATAGAATACTCAAGCTT AACGGGACGGGAGTTGTA
pAK1900- *bswR* -R(FLAG) (*BamHI*)	For overexpressing BswR-FLAG	GAGCTCGGTACCCGGGGATCCTTA CTT ATC GTC GTC ATC CTT GTA ATCCAGTTCACTCCTTGTGCG
pAK1900- *bswR* -R (to 3'UTR) (*Bam*HI)	Insertion of HemL-rG4 to 3' UTR	GAGCTCGGTACCCGGGGATCC GGCCATGGGGACCTGGAGGACCATGGCCAGCTGTTCGGA TTACTTATCGTCGTCATCCTT
BswR-RT-F	For RT-qPCR	CCTACCTCTATTGCCCCGAG
BswR-RT-R	For RT-qPCR	CTGGCGGCTGGTTGATCC
pAK1900- *hemL* -F (*Hind*III)	For overexpressing HemL-FLAG	ACACTATAGAATACTCAAGCTT TCTACGAATCAGGAACCC
pAK1900- *hemL* -R (FLAG) (*BamHI*)	For overexpressing HemL-FLAG	GAGCTCGGTACCCGGGGATCCTTA CTT ATC GTC GTC ATC CTT GTA ATC CAACTTCGCAAACACCCG

pAK1900-*hemL*-R(to3'UTR) (*Bam*HI)	Insertion of BswR-rG4 to 3' UTR	GAGCTCGGTACCCGGGGATCC<br>CGTACCCGCCTGATAGACCGGACCCGTCGGGGCCAGCGCATCTTACTTATCGTCGTCATCCTT
HemL-RT-F	For RT-qPCR	CCTGTCTGAATGAAGTCGCG
HemL-RT-R	For RT-qPCR	GGAATTCCGGCTTCTTCTGC
HemLcodon-mut1-F	For codon mutation	CTTTACAGCGCAGCaCGCGAGCTGATC
Hemcodon-mut1-R	For codon mutation	GATCAGCTCGCGtGCTGCGCTGTAAAG
HemLcodon-mut2-F	For codon mutation	GAAGTGAAAATGGCaCAACTGGTGACCG
Hemcodon-mut2-R	For codon mutation	CGGTCACCAGTTGtGCCATTTTCACTTC
HemLcodon-mut3-F	For codon mutation	TCCGGCACTGAAGCaACCATGAGCGCC
Hemcodon-mut3-R	For codon mutation	GGCGCTCATGGTtGCTTCAGTGCCGGA
HemLcodon-mut4-F	For codon mutation	GGTTCCTGGGGGCCaATGGTGCTGGGC
Hemcodon-mut4-R	For codon mutation	GCCCAGCACCATtGGCCCCCAGGAACC
HemLcodon-mut5-F	For codon mutation	TTTGAGCAATACCCaCAAGAGATTGCC
Hemcodon-mut5-R	For codon mutation	GGCAATCTCTTGtGGGTATTGCTCAAA
HemLcodon-mut6-F	For codon mutation	AACTGTGTTCCaCCaCTGCCAGAGTTC
Hemcodon-mut6-R	For codon mutation	GAACTCTGGCAGtGGtGGAACACAGTT
HemLcodon-mut7-F	For codon mutation	CTCACGTTAGGCCAaCCAAACTCGCCG

Hemcodon-mut7-R	For codon mutation	CGGCGAGTTTGGtTGGCCTAACGTGAG
HemLcodon-mut8-F	For codon mutation	CTAGCTGGCGCACAaGATTATTACGGC
HemLcodon-mut8-R	For codon mutation	GCCGTAATAATCtTGTGCGCCAGCTAG
HemLcodon-mut9-F	For codon mutation	AATGAAGTCGCGCAaCCGGGCGTTCAC
HemLcodon-mut9-R	For codon mutation	GTGAACGCCCGGtTGCGCGACTTCATT
bswRcodon-mut1-F	For codon mutation	GCGGGATACTCGCAaAAACAGTTGGGC
bswRcodon-mut1-R	For codon mutation	GCCCAACTGTTTtTGCGAGTATCCCGC
bswRcodon-mut2-F	For codon mutation	TACTCGCAGAAACAaTTGGGCATGCTG
bswRcodon-mut2-R	For codon mutation	CAGCATGCCCAAtTGTTTCTGCGAGTA
bswRcodon-mut3-F	For codon mutation	CAGTTGGGCATGCTaGTGGGAATGGAC
bswRcodon-mut3-R	For codon mutation	GTCCATTCCCACtAGCATGCCCAACTG
bswRcodon-mut4-F	For codon mutation	CCCGAGGACGAGCTaGCCGAGCTCATC
bswRcodon-mut4-R	For codon mutation	GATGAGCTCGGCtAGCTCGTCCTCGGG
HemL-20-WT-rG4<br>5’ UTR synthetic nucleotide fragment	Insertion of rG4 sequence to 5’ UTR at -20 from ATG	CGAATCAGTTGTCTGCGTCGCAGGCGCGCGGCCTGCGGTGTTTCTAAAGAGGGAGTATCAGTTTTC<br>ATCCAATGATCACTTATTGGTCATACAAATAAGATGACGGCATTTTACTCGCACGTTCCATGAAAATC<br>CTTTGCAAAGCGTAATGTTTCAAATGCACAAAACTTTTATACTCTAAGAAATCAGGATTGCACCAGT<br>ACAAGCAGCCTGATGTTTGACGAGTATTTAACTTGTTATGAATAACATAGAATAGCAGCCATTCACT<br>TTTTGATGCGCTGGCCCCGACGGGTCCGGTCTATCAGGCGGGTACGCTTCTACGAATCAGGAACCC

		TCC
HemL-20-WT-rG4<br>5’ UTR synthetic nucleotide fragment	Insertion of rG4 sequence to 5’ UTR at -20 from ATG	CGAATCAGTTGTCTGCGTCGCAGGCGCGCGGCCTGCGGTGTTTCTAAAGAGGGAGTATCAGTTTTC<br>ATCCAATGATCACTTATTGGTCATACAAATAAGATGACGGCATTTTACTCGCACGTTCCATGAAAATC<br>CTTTGCAAAGCGTAATGTTTCAAATGCACAAAACTTTTATACTCTAAGAAATCAGGATTGCACCAGT<br>ACAAGCAGCCTGATGTTTGACGAGTATTTAACTTGTTATGAATAACATAGAATAGCAGCCATTCACT<br>TTTTGATGCGCTGGCCCCGACGGGTCCaGTCTATCAaGCaGGTACGCTTCTACGAATCAGGAACCCT<br>CC
BswR-20-WT-rG4<br>5’ UTR synthetic nucleotide fragment	Insertion of rG4 sequence to 5’ UTR at -20 from ATG	CCAGGCGACGAAACGGCGTCCGCCTTTATGCAATTCGCATATACTCCGGCTCGCCGCGAGAAAACC<br>GGAGCATGCGCGATCTCGGCGACATCGACGCATATCCCCGCCGCAGCCTCGTACACGGCGTCGAAA<br>CCGCGCGCCGCCCCGGACGACAGAGCCTCCGGCACGGCGAAAACTAAGGATTCCTGAGCTTCCAT<br>CTTGTCCAGATTTGCCCAACCTTGAGCGAGCGGGCCAGACTTCCGAACAGCTGGCCATGGTCCTCC<br>AGGTCCCCATGGCCTAACGGGACGGGAGTTGTATGG
BswR-20-WT-rG4<br>5’ UTR synthetic nucleotide fragment	Insertion of rG4 sequence to 5’ UTR at -20 from ATG	CCCAGGCGACGAAACGGCGTCCGCCTTTATGCAATTCGCATATACTCCGGCTCGCCGCGAGAAAAC<br>CGGAGCATGCGCGATCTCGGCGACATCGACGCATATCCCCGCCGCAGCCTCGTACACGGCGTCGAA<br>ACCGCGCGCCGCCCCGGACGACAGAGCCTCCGGCACGGCGAAAACTAAGGATTCCTGAGCTTCCA<br>TCTTGTCCAGATTTGCCCAACCTTGAGCGAGCGGGCCAGACTTCCGAACAGCTaGCCATGGTCCTC<br>CAaGTCCCCATGGCCTAACGGGACGGGAGTTGTATGG
