# Supplementary material for: RNA G-Quadruplex Structures Mediate Gene Regulation in Bacteria
Source: mBio. 2020 Jan 21;11(1):e02926-19. doi: 10.1128/mBio.02926-19 (PMC6974567; doi:10.1128/mBio.02926-19)
Supplement: TEXT S1 [file mBio.02926-19-s0001.pdf]

## Supplementary References


1. Guo JU, Bartel DP. 2016. RNA G-quadruplexes are globally unfolded in eukaryotic cells and depleted in bacteria. Science 353.
2. Duan K, Dammel C, Stein J, Rabin H, Surette MG. 2003. Modulation of *Pseudomonas aeruginosa* gene expression by host microflora through interspecies communication. Mol Microbiol 50:1477-91.
3. Jansons I, Touchie G, Sharp R, Almquist K, Farinha MA, Lam JS, Kropinski AM. 1994. Deletion and transposon mutagenesis and sequence analysis of the pRO1600 OriR region found in the broad-host-range plasmids of the pQF series. Plasmid 31:265-274.
